# Supplementary material for: Functional decline of the precuneus associated with mild cognitive impairment: Magnetoencephalographic observations
Source: PLoS One. 2020 Sep 28;15(9):e0239577. doi: 10.1371/journal.pone.0239577 (PMC7521706; doi:10.1371/journal.pone.0239577)
Supplement: S1 Data — (PDF) [file pone.0239577.s001.pdf]

				Accuracy of each recall number						
	Group	id	MoCA	1	2	3	4	5	6	7
High	High	E51	27	75	45	25	25	25	45	75
High	High	E52	27	60	55	45	35	35	55	65
High	High	E53	26	80	55	65	55	30	50	80
High	High	E57	26	75	50	25	50	35	40	90
High	High	E63	27	90	70	60	35	30	40	85
High	High	E67	30	60	35	55	20	25	40	85
High	High	E70	26	85	65	45	15	25	50	75
Low	Low	E8	25	80	50	55	60	30	40	100
Low	Low	E11	19	25	25	25	20	30	45	75
Low	Low	E55	23	40	55	30	25	50	40	85
Low	Low	E56	24	25	35	45	25	10	15	55
Low	Low	E58	23	20	45	25	25	40	25	20
Low	Low	E59	25	60	50	65	20	35	65	95
Low	Low	E60	22	60	30	20	20	25	15	40
Low	Low	E61	23	70	65	50	20	20	60	75
Low	Low	E62	20	55	45	45	20	35	40	80
Low	Low	E64	24	70	55	50	40	40	40	70
Low	Low	E66	25	80	70	30	35	20	35	55
Low	Low	E68	20	40	15	35	40	10	45	95
Low	Low	E69	24	45	35	20	40	25	25	55
