# Supplementary material for: Functional decline of the precuneus associated with mild cognitive impairment: Magnetoencephalographic observations
Source: PLoS One. 2020 Sep 28;15(9):e0239577. doi: 10.1371/journal.pone.0239577 (PMC7521706; doi:10.1371/journal.pone.0239577)
Supplement: S2 Data — (PDF) [file pone.0239577.s002.pdf]

| Time (s) | −1.001 | −0.999 | −0.997 | −0.996 | −0.994 | −0.992 | −0.991 | −0.989 | −0.987 | −0.986 | −0.984 | −0.982 | −0.981 | −0.979 | −0.977 |
|---|---|---|---|---|---|---|---|---|---|---|---|---|---|---|---|
| L_PCu | | | | | | | | | | | | | | | |
| high | −0.582 | −0.588 | −0.593 | −0.598 | −0.602 | −0.606 | −0.609 | −0.611 | −0.613 | −0.613 | −0.613 | −0.612 | −0.610 | −0.607 | −0.603 |
| low | −0.730 | −0.730 | −0.729 | −0.728 | −0.725 | −0.722 | −0.719 | −0.715 | −0.711 | −0.706 | −0.700 | −0.693 | −0.685 | −0.675 | −0.665 |
| R_Pcu | | | | | | | | | | | | | | | |
| high | −2.069 | −2.129 | −2.188 | −2.245 | −2.301 | −2.354 | −2.406 | −2.455 | −2.502 | −2.547 | −2.589 | −2.629 | −2.666 | −2.700 | −2.731 |
| low | −0.318 | −0.312 | −0.306 | −0.301 | −0.296 | −0.291 | −0.285 | −0.280 | −0.275 | −0.270 | −0.264 | −0.258 | −0.253 | −0.246 | −0.239 |

| | | | | | | | | | | | | | | | | |
|---|---|---|---|---|---|---|---|---|---|---|---|---|---|---|---|---|
| −0.976 | −0.974 | −0.972 | −0.971 | −0.969 | −0.967 | −0.966 | −0.964 | −0.962 | −0.961 | −0.959 | −0.957 | −0.956 | −0.954 | −0.952 | −0.951 | −0.949 |
| | | | | | | | | | | | | | | | | |
| −0.597 | −0.591 | −0.583 | −0.574 | −0.562 | −0.551 | −0.537 | −0.524 | −0.509 | −0.493 | −0.477 | −0.461 | −0.443 | −0.426 | −0.408 | −0.389 | −0.370 |
| −0.654 | −0.642 | −0.629 | −0.616 | −0.602 | −0.588 | −0.572 | −0.557 | −0.541 | −0.525 | −0.507 | −0.491 | −0.473 | −0.456 | −0.438 | −0.422 | −0.404 |
| | | | | | | | | | | | | | | | | |
| −2.759 | −2.785 | −2.807 | −2.827 | −2.842 | −2.855 | −2.863 | −2.868 | −2.869 | −2.866 | −2.860 | −2.850 | −2.837 | −2.821 | −2.801 | −2.779 | −2.753 |
| −0.230 | −0.222 | −0.211 | −0.202 | −0.190 | −0.180 | −0.167 | −0.156 | −0.143 | −0.131 | −0.117 | −0.105 | −0.091 | −0.078 | −0.064 | −0.051 | −0.036 |

| | | | | | | | | | | | | | | | | |
|---|---|---|---|---|---|---|---|---|---|---|---|---|---|---|---|---|
| −0.947 | −0.946 | −0.944 | −0.942 | −0.941 | −0.939 | −0.937 | −0.936 | −0.934 | −0.932 | −0.931 | −0.929 | −0.927 | −0.926 | −0.924 | −0.922 | −0.921 |
| | | | | | | | | | | | | | | | | |
| −0.350 | −0.330 | −0.309 | −0.288 | −0.266 | −0.245 | −0.223 | −0.202 | −0.181 | −0.161 | −0.141 | −0.122 | −0.103 | −0.085 | −0.067 | −0.051 | −0.037 |
| −0.387 | −0.370 | −0.354 | −0.337 | −0.322 | −0.307 | −0.293 | −0.279 | −0.266 | −0.253 | −0.241 | −0.230 | −0.218 | −0.208 | −0.198 | −0.188 | −0.178 |
| | | | | | | | | | | | | | | | | |
| −2.724 | −2.693 | −2.659 | −2.622 | −2.584 | −2.543 | −2.500 | −2.456 | −2.409 | −2.361 | −2.311 | −2.261 | −2.208 | −2.155 | −2.099 | −2.044 | −1.987 |
| −0.023 | −0.008 | 0.006 | 0.021 | 0.035 | 0.049 | 0.064 | 0.078 | 0.092 | 0.106 | 0.120 | 0.134 | 0.148 | 0.160 | 0.174 | 0.186 | 0.199 |

| | | | | | | | | | | | | | | | | |
|---|---|---|---|---|---|---|---|---|---|---|---|---|---|---|---|---|
| −0.919 | −0.917 | −0.916 | −0.914 | −0.912 | −0.911 | −0.909 | −0.907 | −0.906 | −0.904 | −0.902 | −0.901 | −0.899 | −0.897 | −0.896 | −0.894 | −0.892 |
| | | | | | | | | | | | | | | | | |
| −0.024 | −0.013 | −0.003 | 0.004 | 0.010 | 0.014 | 0.017 | 0.017 | 0.017 | 0.013 | 0.009 | 0.002 | −0.005 | −0.016 | −0.028 | −0.043 | −0.059 |
| −0.170 | −0.160 | −0.151 | −0.142 | −0.133 | −0.124 | −0.116 | −0.106 | −0.098 | −0.089 | −0.080 | −0.070 | −0.061 | −0.051 | −0.042 | −0.031 | −0.021 |
| | | | | | | | | | | | | | | | | |
| −1.930 | −1.872 | −1.815 | −1.756 | −1.698 | −1.639 | −1.581 | −1.522 | −1.464 | −1.407 | −1.349 | −1.293 | −1.237 | −1.183 | −1.128 | −1.076 | −1.024 |
| 0.211 | 0.223 | 0.234 | 0.246 | 0.256 | 0.268 | 0.279 | 0.291 | 0.301 | 0.313 | 0.325 | 0.337 | 0.349 | 0.362 | 0.374 | 0.387 | 0.399 |

| | | | | | | | | | | | | | | | | |
|---|---|---|---|---|---|---|---|---|---|---|---|---|---|---|---|---|
| −0.891 | −0.889 | −0.887 | −0.886 | −0.884 | −0.882 | −0.881 | −0.879 | −0.877 | −0.876 | −0.874 | −0.872 | −0.871 | −0.869 | −0.867 | −0.866 | −0.864 |
| −0.077 | −0.097 | −0.119 | −0.142 | −0.167 | −0.194 | −0.222 | −0.253 | −0.285 | −0.318 | −0.353 | −0.390 | −0.426 | −0.465 | −0.503 | −0.543 | −0.582 |
| −0.009 | 0.002 | 0.014 | 0.026 | 0.038 | 0.052 | 0.064 | 0.079 | 0.092 | 0.107 | 0.121 | 0.136 | 0.150 | 0.165 | 0.179 | 0.195 | 0.210 |
| −0.974 | −0.924 | −0.875 | −0.827 | −0.781 | −0.735 | −0.691 | −0.647 | −0.605 | −0.564 | −0.523 | −0.484 | −0.445 | −0.407 | −0.370 | −0.335 | −0.301 |
| 0.412 | 0.424 | 0.436 | 0.447 | 0.458 | 0.468 | 0.478 | 0.488 | 0.496 | 0.505 | 0.513 | 0.522 | 0.529 | 0.537 | 0.543 | 0.550 | 0.556 |

| | | | | | | | | | | | | | | | | |
|---|---|---|---|---|---|---|---|---|---|---|---|---|---|---|---|---|
| −0.862 | −0.861 | −0.859 | −0.857 | −0.856 | −0.854 | −0.852 | −0.851 | −0.849 | −0.847 | −0.846 | −0.844 | −0.842 | −0.841 | −0.839 | −0.837 | −0.836 |
| | | | | | | | | | | | | | | | | |
| −0.622 | −0.660 | −0.699 | −0.736 | −0.774 | −0.810 | −0.848 | −0.883 | −0.919 | −0.953 | −0.987 | −1.019 | −1.050 | −1.080 | −1.110 | −1.138 | −1.165 |
| 0.226 | 0.241 | 0.257 | 0.273 | 0.289 | 0.306 | 0.323 | 0.340 | 0.357 | 0.375 | 0.393 | 0.412 | 0.430 | 0.449 | 0.467 | 0.485 | 0.503 |
| | | | | | | | | | | | | | | | | |
| −0.268 | −0.235 | −0.205 | −0.175 | −0.146 | −0.118 | −0.092 | −0.065 | −0.040 | −0.015 | 0.008 | 0.031 | 0.052 | 0.072 | 0.092 | 0.110 | 0.127 |
| 0.563 | 0.570 | 0.576 | 0.583 | 0.589 | 0.596 | 0.604 | 0.611 | 0.619 | 0.628 | 0.637 | 0.645 | 0.655 | 0.664 | 0.674 | 0.684 | 0.694 |

| | | | | | | | | | | | | | | | | |
|---|---|---|---|---|---|---|---|---|---|---|---|---|---|---|---|---|
| −0.834 | −0.832 | −0.831 | −0.829 | −0.827 | −0.826 | −0.824 | −0.822 | −0.821 | −0.819 | −0.817 | −0.816 | −0.814 | −0.813 | −0.811 | −0.809 | −0.808 |
| | | | | | | | | | | | | | | | | |
| −1.190 | −1.214 | −1.237 | −1.258 | −1.278 | −1.295 | −1.312 | −1.326 | −1.339 | −1.350 | −1.360 | −1.368 | −1.375 | −1.380 | −1.384 | −1.385 | −1.386 |
| 0.522 | 0.540 | 0.558 | 0.576 | 0.595 | 0.613 | 0.631 | 0.649 | 0.667 | 0.685 | 0.702 | 0.719 | 0.736 | 0.751 | 0.767 | 0.781 | 0.795 |
| | | | | | | | | | | | | | | | | |
| 0.143 | 0.158 | 0.172 | 0.186 | 0.199 | 0.212 | 0.224 | 0.236 | 0.247 | 0.257 | 0.267 | 0.276 | 0.284 | 0.293 | 0.300 | 0.307 | 0.313 |
| 0.704 | 0.715 | 0.725 | 0.736 | 0.746 | 0.757 | 0.768 | 0.779 | 0.790 | 0.801 | 0.812 | 0.824 | 0.835 | 0.846 | 0.857 | 0.868 | 0.878 |

| | | | | | | | | | | | | | | | | |
|---|---|---|---|---|---|---|---|---|---|---|---|---|---|---|---|---|
| −0.806 | −0.804 | −0.803 | −0.801 | −0.799 | −0.798 | −0.796 | −0.794 | −0.793 | −0.791 | −0.789 | −0.788 | −0.786 | −0.784 | −0.783 | −0.781 | −0.779 |
| −1.384 | −1.380 | −1.374 | −1.367 | −1.357 | −1.346 | −1.332 | −1.317 | −1.300 | −1.282 | −1.262 | −1.241 | −1.219 | −1.196 | −1.172 | −1.147 | −1.120 |
| 0.808 | 0.819 | 0.830 | 0.839 | 0.847 | 0.852 | 0.857 | 0.860 | 0.862 | 0.861 | 0.860 | 0.856 | 0.851 | 0.843 | 0.835 | 0.823 | 0.810 |
| 0.320 | 0.327 | 0.333 | 0.339 | 0.346 | 0.351 | 0.357 | 0.363 | 0.369 | 0.375 | 0.381 | 0.388 | 0.395 | 0.402 | 0.410 | 0.419 | 0.428 |
| 0.888 | 0.898 | 0.907 | 0.916 | 0.924 | 0.931 | 0.937 | 0.942 | 0.947 | 0.950 | 0.952 | 0.953 | 0.952 | 0.951 | 0.948 | 0.945 | 0.940 |

| | | | | | | | | | | | | | | | | |
|---|---|---|---|---|---|---|---|---|---|---|---|---|---|---|---|---|
| −0.778 | −0.776 | −0.774 | −0.773 | −0.771 | −0.769 | −0.768 | −0.766 | −0.764 | −0.763 | −0.761 | −0.759 | −0.758 | −0.756 | −0.754 | −0.753 | −0.751 |
| | | | | | | | | | | | | | | | | |
| −1.094 | −1.066 | −1.037 | −1.008 | −0.978 | −0.948 | −0.918 | −0.889 | −0.859 | −0.829 | −0.800 | −0.771 | −0.742 | −0.713 | −0.685 | −0.657 | −0.628 |
| 0.795 | 0.778 | 0.760 | 0.739 | 0.717 | 0.693 | 0.668 | 0.641 | 0.614 | 0.585 | 0.555 | 0.525 | 0.494 | 0.462 | 0.430 | 0.397 | 0.363 |
| | | | | | | | | | | | | | | | | |
| 0.437 | 0.447 | 0.457 | 0.468 | 0.479 | 0.490 | 0.502 | 0.515 | 0.528 | 0.541 | 0.555 | 0.570 | 0.585 | 0.600 | 0.616 | 0.632 | 0.649 |
| 0.933 | 0.926 | 0.917 | 0.906 | 0.894 | 0.879 | 0.864 | 0.847 | 0.828 | 0.808 | 0.786 | 0.763 | 0.739 | 0.713 | 0.686 | 0.658 | 0.628 |

| | | | | | | | | | | | | | | | | |
|---|---|---|---|---|---|---|---|---|---|---|---|---|---|---|---|---|
| −0.749 | −0.748 | −0.746 | −0.744 | −0.743 | −0.741 | −0.739 | −0.738 | −0.736 | −0.734 | −0.733 | −0.731 | −0.729 | −0.728 | −0.726 | −0.724 | −0.723 |
| −0.600 | −0.572 | −0.544 | −0.516 | −0.489 | −0.461 | −0.434 | −0.407 | −0.379 | −0.351 | −0.324 | −0.296 | −0.268 | −0.240 | −0.213 | −0.184 | −0.157 |
| 0.329 | 0.295 | 0.261 | 0.227 | 0.193 | 0.160 | 0.127 | 0.094 | 0.061 | 0.029 | −0.003 | −0.035 | −0.066 | −0.097 | −0.128 | −0.158 | −0.188 |
| 0.665 | 0.681 | 0.698 | 0.714 | 0.730 | 0.746 | 0.762 | 0.777 | 0.792 | 0.806 | 0.820 | 0.833 | 0.845 | 0.856 | 0.866 | 0.876 | 0.884 |
| 0.598 | 0.566 | 0.534 | 0.500 | 0.466 | 0.431 | 0.394 | 0.357 | 0.320 | 0.281 | 0.243 | 0.204 | 0.165 | 0.126 | 0.087 | 0.048 | 0.011 |

| | | | | | | | | | | | | | | | | |
|---|---|---|---|---|---|---|---|---|---|---|---|---|---|---|---|---|
| −0.721 | −0.719 | −0.718 | −0.716 | −0.714 | −0.713 | −0.711 | −0.709 | −0.708 | −0.706 | −0.704 | −0.703 | −0.701 | −0.699 | −0.698 | −0.696 | −0.694 |
| | | | | | | | | | | | | | | | | |
| −0.130 | −0.103 | −0.076 | −0.050 | −0.025 | −0.001 | 0.023 | 0.045 | 0.066 | 0.085 | 0.103 | 0.119 | 0.134 | 0.148 | 0.160 | 0.172 | 0.181 |
| −0.217 | −0.245 | −0.273 | −0.299 | −0.325 | −0.349 | −0.373 | −0.395 | −0.417 | −0.437 | −0.457 | −0.476 | −0.493 | −0.509 | −0.524 | −0.538 | −0.551 |
| | | | | | | | | | | | | | | | | |
| 0.892 | 0.898 | 0.905 | 0.909 | 0.914 | 0.917 | 0.921 | 0.922 | 0.924 | 0.924 | 0.922 | 0.920 | 0.916 | 0.911 | 0.904 | 0.896 | 0.886 |
| −0.028 | −0.065 | −0.102 | −0.138 | −0.174 | −0.209 | −0.244 | −0.278 | −0.313 | −0.346 | −0.379 | −0.412 | −0.444 | −0.476 | −0.507 | −0.538 | −0.567 |

| | | | | | | | | | | | | | | | | |
|---|---|---|---|---|---|---|---|---|---|---|---|---|---|---|---|---|
| −0.693 | −0.691 | −0.689 | −0.688 | −0.686 | −0.684 | −0.683 | −0.681 | −0.679 | −0.678 | −0.676 | −0.674 | −0.673 | −0.671 | −0.669 | −0.668 | −0.666 |
| 0.190 | 0.197 | 0.203 | 0.208 | 0.212 | 0.213 | 0.213 | 0.210 | 0.207 | 0.200 | 0.192 | 0.182 | 0.171 | 0.156 | 0.142 | 0.125 | 0.107 |
| −0.563 | −0.573 | −0.583 | −0.591 | −0.599 | −0.605 | −0.610 | −0.614 | −0.617 | −0.618 | −0.619 | −0.619 | −0.618 | −0.616 | −0.614 | −0.610 | −0.606 |
| 0.874 | 0.860 | 0.845 | 0.827 | 0.808 | 0.787 | 0.765 | 0.740 | 0.715 | 0.686 | 0.657 | 0.626 | 0.594 | 0.559 | 0.524 | 0.487 | 0.451 |
| −0.596 | −0.625 | −0.652 | −0.679 | −0.705 | −0.729 | −0.752 | −0.773 | −0.793 | −0.812 | −0.829 | −0.844 | −0.858 | −0.869 | −0.880 | −0.888 | −0.895 |

| | | | | | | | | | | | | | | | | |
|---|---|---|---|---|---|---|---|---|---|---|---|---|---|---|---|---|
| −0.664 | −0.663 | −0.661 | −0.659 | −0.658 | −0.656 | −0.654 | −0.653 | −0.651 | −0.649 | −0.648 | −0.646 | −0.644 | −0.643 | −0.641 | −0.639 | −0.638 |
| | | | | | | | | | | | | | | | | |
| 0.088 | 0.069 | 0.047 | 0.025 | 0.001 | −0.024 | −0.050 | −0.077 | −0.105 | −0.134 | −0.164 | −0.194 | −0.226 | −0.258 | −0.290 | −0.323 | −0.355 |
| −0.600 | −0.594 | −0.586 | −0.577 | −0.567 | −0.555 | −0.542 | −0.528 | −0.513 | −0.497 | −0.479 | −0.460 | −0.441 | −0.420 | −0.399 | −0.376 | −0.354 |
| | | | | | | | | | | | | | | | | |
| 0.412 | 0.374 | 0.335 | 0.295 | 0.254 | 0.212 | 0.169 | 0.125 | 0.081 | 0.037 | −0.008 | −0.054 | −0.099 | −0.145 | −0.191 | −0.238 | −0.283 |
| −0.900 | −0.903 | −0.904 | −0.905 | −0.903 | −0.900 | −0.896 | −0.890 | −0.882 | −0.874 | −0.864 | −0.853 | −0.841 | −0.828 | −0.814 | −0.799 | −0.784 |

| | | | | | | | | | | | | | | | | |
|---|---|---|---|---|---|---|---|---|---|---|---|---|---|---|---|---|
| −0.636 | −0.634 | −0.633 | −0.631 | −0.629 | −0.628 | −0.626 | −0.624 | −0.623 | −0.621 | −0.619 | −0.618 | −0.616 | −0.614 | −0.613 | −0.611 | −0.609 |
| | | | | | | | | | | | | | | | | |
| −0.387 | −0.418 | −0.448 | −0.477 | −0.506 | −0.532 | −0.559 | −0.583 | −0.606 | −0.627 | −0.647 | −0.664 | −0.680 | −0.693 | −0.706 | −0.715 | −0.723 |
| −0.330 | −0.306 | −0.281 | −0.257 | −0.231 | −0.206 | −0.180 | −0.155 | −0.129 | −0.104 | −0.079 | −0.054 | −0.029 | −0.006 | 0.018 | 0.041 | 0.064 |
| | | | | | | | | | | | | | | | | |
| −0.329 | −0.373 | −0.418 | −0.460 | −0.501 | −0.540 | −0.578 | −0.612 | −0.645 | −0.674 | −0.700 | −0.722 | −0.742 | −0.758 | −0.772 | −0.781 | −0.789 |
| −0.768 | −0.752 | −0.734 | −0.717 | −0.699 | −0.682 | −0.664 | −0.646 | −0.627 | −0.609 | −0.590 | −0.572 | −0.554 | −0.537 | −0.519 | −0.502 | −0.485 |

| | | | | | | | | | | | | | | | | |
|---|---|---|---|---|---|---|---|---|---|---|---|---|---|---|---|---|
| −0.608 | −0.606 | −0.604 | −0.603 | −0.601 | −0.599 | −0.598 | −0.596 | −0.594 | −0.593 | −0.591 | −0.589 | −0.588 | −0.586 | −0.584 | −0.583 | −0.581 |
| −0.728 | −0.733 | −0.735 | −0.737 | −0.736 | −0.735 | −0.731 | −0.727 | −0.720 | −0.713 | −0.704 | −0.693 | −0.682 | −0.669 | −0.655 | −0.639 | −0.623 |
| 0.087 | 0.109 | 0.130 | 0.151 | 0.171 | 0.191 | 0.210 | 0.228 | 0.245 | 0.262 | 0.278 | 0.293 | 0.307 | 0.320 | 0.333 | 0.345 | 0.356 |
| −0.794 | −0.796 | −0.795 | −0.792 | −0.787 | −0.780 | −0.772 | −0.761 | −0.750 | −0.737 | −0.723 | −0.708 | −0.692 | −0.675 | −0.657 | −0.638 | −0.618 |
| −0.468 | −0.452 | −0.436 | −0.420 | −0.404 | −0.389 | −0.374 | −0.359 | −0.344 | −0.330 | −0.315 | −0.301 | −0.287 | −0.273 | −0.259 | −0.245 | −0.231 |

| | | | | | | | | | | | | | | | | |
|---|---|---|---|---|---|---|---|---|---|---|---|---|---|---|---|---|
| −0.579 | −0.578 | −0.576 | −0.574 | −0.573 | −0.571 | −0.569 | −0.568 | −0.566 | −0.564 | −0.563 | −0.561 | −0.559 | −0.558 | −0.556 | −0.554 | −0.553 |
| | | | | | | | | | | | | | | | | |
| −0.606 | −0.587 | −0.568 | −0.548 | −0.526 | −0.504 | −0.481 | −0.457 | −0.432 | −0.406 | −0.378 | −0.350 | −0.321 | −0.291 | −0.260 | −0.229 | −0.198 |
| 0.366 | 0.376 | 0.384 | 0.392 | 0.399 | 0.405 | 0.410 | 0.414 | 0.417 | 0.419 | 0.420 | 0.420 | 0.419 | 0.417 | 0.414 | 0.410 | 0.406 |
| | | | | | | | | | | | | | | | | |
| −0.597 | −0.576 | −0.553 | −0.530 | −0.505 | −0.481 | −0.455 | −0.428 | −0.400 | −0.371 | −0.342 | −0.312 | −0.282 | −0.251 | −0.221 | −0.189 | −0.157 |
| −0.216 | −0.200 | −0.185 | −0.169 | −0.152 | −0.134 | −0.117 | −0.098 | −0.080 | −0.061 | −0.042 | −0.023 | −0.004 | 0.016 | 0.034 | 0.053 | 0.072 |

| | | | | | | | | | | | | | | | | |
|---|---|---|---|---|---|---|---|---|---|---|---|---|---|---|---|---|
| −0.551 | −0.549 | −0.548 | −0.546 | −0.544 | −0.543 | −0.541 | −0.539 | −0.538 | −0.536 | −0.534 | −0.533 | −0.531 | −0.529 | −0.528 | −0.526 | −0.524 |
| | | | | | | | | | | | | | | | | |
| −0.166 | −0.134 | −0.102 | −0.070 | −0.037 | −0.004 | 0.029 | 0.062 | 0.095 | 0.129 | 0.162 | 0.196 | 0.230 | 0.265 | 0.299 | 0.333 | 0.367 |
| 0.400 | 0.395 | 0.389 | 0.382 | 0.375 | 0.369 | 0.362 | 0.355 | 0.348 | 0.342 | 0.335 | 0.329 | 0.322 | 0.316 | 0.308 | 0.302 | 0.294 |
| | | | | | | | | | | | | | | | | |
| −0.124 | −0.091 | −0.057 | −0.023 | 0.012 | 0.048 | 0.084 | 0.120 | 0.157 | 0.193 | 0.229 | 0.265 | 0.301 | 0.337 | 0.373 | 0.409 | 0.444 |
| 0.090 | 0.108 | 0.126 | 0.144 | 0.161 | 0.177 | 0.193 | 0.210 | 0.225 | 0.240 | 0.255 | 0.270 | 0.284 | 0.298 | 0.312 | 0.326 | 0.338 |

| | | | | | | | | | | | | | | | | |
|---|---|---|---|---|---|---|---|---|---|---|---|---|---|---|---|---|
| −0.523 | −0.521 | −0.519 | −0.518 | −0.516 | −0.514 | −0.513 | −0.511 | −0.509 | −0.508 | −0.506 | −0.504 | −0.503 | −0.501 | −0.499 | −0.498 | −0.496 |
| | | | | | | | | | | | | | | | | |
| 0.402 | 0.435 | 0.469 | 0.502 | 0.534 | 0.566 | 0.597 | 0.627 | 0.657 | 0.686 | 0.714 | 0.741 | 0.767 | 0.792 | 0.815 | 0.838 | 0.859 |
| 0.288 | 0.281 | 0.274 | 0.266 | 0.259 | 0.252 | 0.245 | 0.237 | 0.230 | 0.222 | 0.214 | 0.206 | 0.198 | 0.190 | 0.181 | 0.173 | 0.164 |
| | | | | | | | | | | | | | | | | |
| 0.479 | 0.513 | 0.547 | 0.580 | 0.611 | 0.642 | 0.672 | 0.701 | 0.728 | 0.755 | 0.780 | 0.805 | 0.829 | 0.851 | 0.872 | 0.892 | 0.911 |
| 0.352 | 0.365 | 0.378 | 0.390 | 0.404 | 0.416 | 0.430 | 0.442 | 0.456 | 0.469 | 0.482 | 0.496 | 0.509 | 0.523 | 0.536 | 0.550 | 0.564 |

| | | | | | | | | | | | | | | | | |
|---|---|---|---|---|---|---|---|---|---|---|---|---|---|---|---|---|
| −0.494 | −0.493 | −0.491 | −0.489 | −0.488 | −0.486 | −0.485 | −0.483 | −0.481 | −0.480 | −0.478 | −0.476 | −0.475 | −0.473 | −0.471 | −0.470 | −0.468 |
| 0.879 | 0.897 | 0.914 | 0.930 | 0.944 | 0.956 | 0.967 | 0.977 | 0.985 | 0.991 | 0.996 | 0.999 | 1.001 | 1.001 | 1.000 | 0.997 | 0.993 |
| 0.156 | 0.148 | 0.140 | 0.132 | 0.124 | 0.116 | 0.108 | 0.099 | 0.091 | 0.083 | 0.074 | 0.065 | 0.057 | 0.048 | 0.040 | 0.032 | 0.024 |
| 0.928 | 0.943 | 0.957 | 0.970 | 0.982 | 0.992 | 1.001 | 1.008 | 1.014 | 1.018 | 1.021 | 1.023 | 1.023 | 1.022 | 1.019 | 1.016 | 1.011 |
| 0.577 | 0.590 | 0.603 | 0.614 | 0.626 | 0.636 | 0.645 | 0.653 | 0.660 | 0.665 | 0.669 | 0.672 | 0.675 | 0.676 | 0.676 | 0.675 | 0.674 |

| | | | | | | | | | | | | | | | | |
|---|---|---|---|---|---|---|---|---|---|---|---|---|---|---|---|---|
| −0.466 | −0.465 | −0.463 | −0.461 | −0.460 | −0.458 | −0.456 | −0.455 | −0.453 | −0.451 | −0.450 | −0.448 | −0.446 | −0.445 | −0.443 | −0.441 | −0.440 |
| | | | | | | | | | | | | | | | | |
| 0.987 | 0.981 | 0.973 | 0.964 | 0.955 | 0.945 | 0.933 | 0.922 | 0.909 | 0.896 | 0.882 | 0.869 | 0.855 | 0.841 | 0.828 | 0.815 | 0.801 |
| 0.016 | 0.009 | 0.002 | −0.005 | −0.012 | −0.018 | −0.025 | −0.031 | −0.036 | −0.042 | −0.047 | −0.052 | −0.056 | −0.060 | −0.063 | −0.065 | −0.067 |
| | | | | | | | | | | | | | | | | |
| 1.005 | 0.999 | 0.992 | 0.984 | 0.975 | 0.966 | 0.956 | 0.945 | 0.934 | 0.922 | 0.910 | 0.899 | 0.887 | 0.875 | 0.864 | 0.853 | 0.842 |
| 0.671 | 0.667 | 0.663 | 0.658 | 0.653 | 0.647 | 0.642 | 0.635 | 0.629 | 0.622 | 0.616 | 0.609 | 0.602 | 0.596 | 0.590 | 0.584 | 0.579 |

| | | | | | | | | | | | | | | | | |
|---|---|---|---|---|---|---|---|---|---|---|---|---|---|---|---|---|
| −0.438 | −0.436 | −0.435 | −0.433 | −0.431 | −0.430 | −0.428 | −0.426 | −0.425 | −0.423 | −0.421 | −0.420 | −0.418 | −0.416 | −0.415 | −0.413 | −0.411 |
| 0.789 | 0.777 | 0.765 | 0.753 | 0.741 | 0.731 | 0.720 | 0.710 | 0.701 | 0.693 | 0.684 | 0.677 | 0.670 | 0.665 | 0.659 | 0.655 | 0.651 |
| −0.068 | −0.069 | −0.069 | −0.069 | −0.067 | −0.066 | −0.063 | −0.060 | −0.057 | −0.054 | −0.050 | −0.046 | −0.042 | −0.037 | −0.033 | −0.028 | −0.023 |
| 0.831 | 0.820 | 0.810 | 0.799 | 0.789 | 0.779 | 0.769 | 0.759 | 0.749 | 0.740 | 0.730 | 0.722 | 0.713 | 0.705 | 0.697 | 0.691 | 0.684 |
| 0.573 | 0.569 | 0.565 | 0.561 | 0.557 | 0.554 | 0.551 | 0.548 | 0.546 | 0.544 | 0.542 | 0.540 | 0.539 | 0.538 | 0.537 | 0.536 | 0.535 |

| | | | | | | | | | | | | | | | | |
|---|---|---|---|---|---|---|---|---|---|---|---|---|---|---|---|---|
| −0.410 | −0.408 | −0.406 | −0.405 | −0.403 | −0.401 | −0.400 | −0.398 | −0.396 | −0.395 | −0.393 | −0.391 | −0.390 | −0.388 | −0.386 | −0.385 | −0.383 |
| | | | | | | | | | | | | | | | | |
| 0.648 | 0.645 | 0.643 | 0.641 | 0.641 | 0.640 | 0.640 | 0.640 | 0.641 | 0.642 | 0.644 | 0.645 | 0.648 | 0.650 | 0.654 | 0.658 | 0.664 |
| −0.018 | −0.013 | −0.009 | −0.004 | 0.000 | 0.004 | 0.008 | 0.012 | 0.015 | 0.018 | 0.021 | 0.023 | 0.026 | 0.029 | 0.032 | 0.035 | 0.039 |
| | | | | | | | | | | | | | | | | |
| 0.679 | 0.675 | 0.671 | 0.667 | 0.664 | 0.662 | 0.661 | 0.660 | 0.660 | 0.660 | 0.661 | 0.663 | 0.666 | 0.670 | 0.673 | 0.678 | 0.684 |
| 0.535 | 0.535 | 0.536 | 0.537 | 0.538 | 0.540 | 0.543 | 0.545 | 0.548 | 0.551 | 0.555 | 0.559 | 0.563 | 0.567 | 0.573 | 0.577 | 0.583 |

| | | | | | | | | | | | | | | | | |
|---|---|---|---|---|---|---|---|---|---|---|---|---|---|---|---|---|
| −0.381 | −0.380 | −0.378 | −0.376 | −0.375 | −0.373 | −0.371 | −0.370 | −0.368 | −0.366 | −0.365 | −0.363 | −0.361 | −0.360 | −0.358 | −0.356 | −0.355 |
| | | | | | | | | | | | | | | | | |
| 0.670 | 0.678 | 0.688 | 0.698 | 0.711 | 0.723 | 0.737 | 0.751 | 0.767 | 0.781 | 0.797 | 0.811 | 0.827 | 0.841 | 0.856 | 0.869 | 0.883 |
| 0.043 | 0.047 | 0.051 | 0.055 | 0.059 | 0.063 | 0.067 | 0.071 | 0.074 | 0.077 | 0.080 | 0.083 | 0.085 | 0.087 | 0.088 | 0.089 | 0.090 |
| | | | | | | | | | | | | | | | | |
| 0.690 | 0.697 | 0.704 | 0.712 | 0.721 | 0.730 | 0.739 | 0.749 | 0.759 | 0.769 | 0.780 | 0.790 | 0.801 | 0.811 | 0.822 | 0.832 | 0.842 |
| 0.588 | 0.594 | 0.599 | 0.604 | 0.609 | 0.614 | 0.618 | 0.621 | 0.624 | 0.626 | 0.627 | 0.628 | 0.628 | 0.627 | 0.626 | 0.623 | 0.620 |

| | | | | | | | | | | | | | | | | |
|---|---|---|---|---|---|---|---|---|---|---|---|---|---|---|---|---|
| −0.353 | −0.351 | −0.350 | −0.348 | −0.346 | −0.345 | −0.343 | −0.341 | −0.340 | −0.338 | −0.336 | −0.335 | −0.333 | −0.331 | −0.330 | −0.328 | −0.326 |
| | | | | | | | | | | | | | | | | |
| 0.896 | 0.908 | 0.918 | 0.928 | 0.936 | 0.944 | 0.949 | 0.954 | 0.956 | 0.958 | 0.957 | 0.955 | 0.952 | 0.948 | 0.942 | 0.935 | 0.927 |
| 0.091 | 0.091 | 0.091 | 0.090 | 0.089 | 0.088 | 0.087 | 0.085 | 0.083 | 0.082 | 0.080 | 0.079 | 0.077 | 0.076 | 0.075 | 0.075 | 0.074 |
| | | | | | | | | | | | | | | | | |
| 0.852 | 0.863 | 0.873 | 0.883 | 0.892 | 0.902 | 0.910 | 0.918 | 0.925 | 0.932 | 0.937 | 0.942 | 0.947 | 0.950 | 0.953 | 0.954 | 0.955 |
| 0.616 | 0.611 | 0.605 | 0.598 | 0.590 | 0.582 | 0.572 | 0.562 | 0.551 | 0.539 | 0.527 | 0.515 | 0.501 | 0.488 | 0.474 | 0.460 | 0.446 |

| | | | | | | | | | | | | | | | | |
|---|---|---|---|---|---|---|---|---|---|---|---|---|---|---|---|---|
| −0.325 | −0.323 | −0.321 | −0.320 | −0.318 | −0.316 | −0.315 | −0.313 | −0.311 | −0.310 | −0.308 | −0.306 | −0.305 | −0.303 | −0.301 | −0.300 | −0.298 |
| 0.917 | 0.907 | 0.895 | 0.883 | 0.870 | 0.857 | 0.843 | 0.829 | 0.815 | 0.801 | 0.786 | 0.772 | 0.757 | 0.743 | 0.727 | 0.713 | 0.698 |
| 0.074 | 0.073 | 0.073 | 0.073 | 0.073 | 0.072 | 0.072 | 0.071 | 0.070 | 0.068 | 0.066 | 0.064 | 0.061 | 0.058 | 0.054 | 0.050 | 0.046 |
| 0.956 | 0.956 | 0.955 | 0.954 | 0.951 | 0.948 | 0.945 | 0.941 | 0.936 | 0.932 | 0.927 | 0.922 | 0.917 | 0.911 | 0.905 | 0.900 | 0.894 |
| 0.431 | 0.416 | 0.400 | 0.385 | 0.368 | 0.352 | 0.334 | 0.317 | 0.298 | 0.280 | 0.260 | 0.240 | 0.219 | 0.198 | 0.175 | 0.153 | 0.129 |

| | | | | | | | | | | | | | | | | |
|---|---|---|---|---|---|---|---|---|---|---|---|---|---|---|---|---|
| −0.296 | −0.295 | −0.293 | −0.291 | −0.290 | −0.288 | −0.286 | −0.285 | −0.283 | −0.281 | −0.280 | −0.278 | −0.276 | −0.275 | −0.273 | −0.271 | −0.270 |
| 0.684 | 0.670 | 0.656 | 0.643 | 0.629 | 0.615 | 0.601 | 0.587 | 0.573 | 0.558 | 0.544 | 0.529 | 0.514 | 0.500 | 0.486 | 0.471 | 0.458 |
| 0.041 | 0.036 | 0.031 | 0.026 | 0.021 | 0.015 | 0.010 | 0.005 | 0.001 | −0.003 | −0.007 | −0.010 | −0.013 | −0.015 | −0.017 | −0.019 | −0.021 |
| 0.888 | 0.881 | 0.874 | 0.866 | 0.858 | 0.849 | 0.839 | 0.829 | 0.817 | 0.805 | 0.792 | 0.778 | 0.763 | 0.748 | 0.731 | 0.713 | 0.694 |
| 0.106 | 0.081 | 0.057 | 0.031 | 0.006 | −0.020 | −0.045 | −0.071 | −0.097 | −0.124 | −0.150 | −0.176 | −0.202 | −0.228 | −0.254 | −0.279 | −0.304 |

| | | | | | | | | | | | | | | | | |
|---|---|---|---|---|---|---|---|---|---|---|---|---|---|---|---|---|
| −0.268 | −0.266 | −0.265 | −0.263 | −0.261 | −0.260 | −0.258 | −0.256 | −0.255 | −0.253 | −0.251 | −0.250 | −0.248 | −0.246 | −0.245 | −0.243 | −0.241 |
| | | | | | | | | | | | | | | | | |
| 0.444 | 0.432 | 0.419 | 0.406 | 0.394 | 0.382 | 0.369 | 0.357 | 0.344 | 0.331 | 0.318 | 0.305 | 0.291 | 0.277 | 0.263 | 0.248 | 0.233 |
| −0.022 | −0.024 | −0.024 | −0.025 | −0.025 | −0.025 | −0.026 | −0.026 | −0.026 | −0.027 | −0.028 | −0.030 | −0.032 | −0.035 | −0.038 | −0.042 | −0.047 |
| | | | | | | | | | | | | | | | | |
| 0.673 | 0.652 | 0.630 | 0.608 | 0.584 | 0.559 | 0.534 | 0.508 | 0.482 | 0.455 | 0.428 | 0.400 | 0.372 | 0.343 | 0.314 | 0.285 | 0.256 |
| −0.329 | −0.354 | −0.378 | −0.403 | −0.426 | −0.450 | −0.474 | −0.497 | −0.520 | −0.544 | −0.566 | −0.590 | −0.612 | −0.635 | −0.658 | −0.680 | −0.702 |

| | | | | | | | | | | | | | | | | |
|---|---|---|---|---|---|---|---|---|---|---|---|---|---|---|---|---|
| −0.240 | −0.238 | −0.236 | −0.235 | −0.233 | −0.231 | −0.230 | −0.228 | −0.226 | −0.225 | −0.223 | −0.221 | −0.220 | −0.218 | −0.216 | −0.215 | −0.213 |
| | | | | | | | | | | | | | | | | |
| 0.218 | 0.203 | 0.187 | 0.171 | 0.155 | 0.140 | 0.123 | 0.107 | 0.090 | 0.074 | 0.057 | 0.041 | 0.024 | 0.008 | −0.009 | −0.025 | −0.040 |
| −0.052 | −0.057 | −0.063 | −0.068 | −0.075 | −0.081 | −0.088 | −0.095 | −0.102 | −0.110 | −0.119 | −0.128 | −0.137 | −0.147 | −0.157 | −0.168 | −0.179 |
| | | | | | | | | | | | | | | | | |
| 0.227 | 0.198 | 0.169 | 0.140 | 0.111 | 0.083 | 0.054 | 0.026 | −0.003 | −0.030 | −0.058 | −0.085 | −0.111 | −0.136 | −0.161 | −0.185 | −0.208 |
| −0.723 | −0.744 | −0.764 | −0.784 | −0.803 | −0.822 | −0.840 | −0.858 | −0.876 | −0.893 | −0.909 | −0.925 | −0.940 | −0.956 | −0.970 | −0.984 | −0.998 |

| | | | | | | | | | | | | | | | | |
|---|---|---|---|---|---|---|---|---|---|---|---|---|---|---|---|---|
| −0.211 | −0.210 | −0.208 | −0.206 | −0.205 | −0.203 | −0.201 | −0.200 | −0.198 | −0.196 | −0.195 | −0.193 | −0.191 | −0.190 | −0.188 | −0.186 | −0.185 |
| | | | | | | | | | | | | | | | | |
| −0.055 | −0.070 | −0.084 | −0.097 | −0.110 | −0.122 | −0.133 | −0.144 | −0.155 | −0.164 | −0.174 | −0.182 | −0.190 | −0.197 | −0.204 | −0.210 | −0.216 |
| −0.191 | −0.203 | −0.216 | −0.230 | −0.243 | −0.258 | −0.272 | −0.286 | −0.301 | −0.316 | −0.330 | −0.345 | −0.360 | −0.374 | −0.389 | −0.403 | −0.417 |
| | | | | | | | | | | | | | | | | |
| −0.231 | −0.252 | −0.273 | −0.293 | −0.312 | −0.329 | −0.346 | −0.362 | −0.376 | −0.389 | −0.401 | −0.413 | −0.422 | −0.431 | −0.439 | −0.447 | −0.453 |
| −1.010 | −1.022 | −1.034 | −1.044 | −1.054 | −1.064 | −1.073 | −1.082 | −1.090 | −1.098 | −1.106 | −1.113 | −1.119 | −1.125 | −1.131 | −1.136 | −1.140 |

| | | | | | | | | | | | | | | | | |
|---|---|---|---|---|---|---|---|---|---|---|---|---|---|---|---|---|
| −0.183 | −0.181 | −0.180 | −0.178 | −0.176 | −0.175 | −0.173 | −0.171 | −0.170 | −0.168 | −0.166 | −0.165 | −0.163 | −0.162 | −0.160 | −0.158 | −0.157 |
| | | | | | | | | | | | | | | | | |
| −0.221 | −0.227 | −0.233 | −0.239 | −0.244 | −0.251 | −0.257 | −0.265 | −0.272 | −0.280 | −0.289 | −0.298 | −0.307 | −0.316 | −0.326 | −0.335 | −0.345 |
| −0.432 | −0.445 | −0.459 | −0.473 | −0.486 | −0.499 | −0.512 | −0.524 | −0.536 | −0.548 | −0.559 | −0.569 | −0.579 | −0.589 | −0.598 | −0.606 | −0.614 |
| | | | | | | | | | | | | | | | | |
| −0.459 | −0.465 | −0.470 | −0.475 | −0.480 | −0.484 | −0.489 | −0.493 | −0.497 | −0.501 | −0.504 | −0.508 | −0.511 | −0.514 | −0.518 | −0.521 | −0.525 |
| −1.144 | −1.148 | −1.151 | −1.154 | −1.157 | −1.159 | −1.162 | −1.164 | −1.166 | −1.167 | −1.169 | −1.170 | −1.170 | −1.170 | −1.170 | −1.169 | −1.168 |

| | | | | | | | | | | | | | | | | |
|---|---|---|---|---|---|---|---|---|---|---|---|---|---|---|---|---|
| −0.155 | −0.153 | −0.152 | −0.150 | −0.148 | −0.147 | −0.145 | −0.143 | −0.142 | −0.140 | −0.138 | −0.137 | −0.135 | −0.133 | −0.132 | −0.130 | −0.128 |
| | | | | | | | | | | | | | | | | |
| −0.355 | −0.364 | −0.374 | −0.384 | −0.394 | −0.405 | −0.415 | −0.427 | −0.437 | −0.449 | −0.460 | −0.472 | −0.483 | −0.494 | −0.505 | −0.516 | −0.526 |
| −0.621 | −0.627 | −0.633 | −0.638 | −0.642 | −0.645 | −0.648 | −0.650 | −0.651 | −0.651 | −0.651 | −0.650 | −0.648 | −0.645 | −0.642 | −0.638 | −0.633 |
| | | | | | | | | | | | | | | | | |
| −0.529 | −0.532 | −0.535 | −0.538 | −0.540 | −0.541 | −0.542 | −0.543 | −0.544 | −0.544 | −0.545 | −0.545 | −0.545 | −0.545 | −0.544 | −0.544 | −0.543 |
| −1.166 | −1.164 | −1.161 | −1.157 | −1.153 | −1.149 | −1.143 | −1.137 | −1.131 | −1.124 | −1.116 | −1.107 | −1.098 | −1.087 | −1.077 | −1.066 | −1.054 |

| | | | | | | | | | | | | | | | | |
|---|---|---|---|---|---|---|---|---|---|---|---|---|---|---|---|---|
| −0.127 | −0.125 | −0.123 | −0.122 | −0.120 | −0.118 | −0.117 | −0.115 | −0.113 | −0.112 | −0.110 | −0.108 | −0.107 | −0.105 | −0.103 | −0.102 | −0.100 |
| | | | | | | | | | | | | | | | | |
| −0.536 | −0.545 | −0.554 | −0.562 | −0.570 | −0.577 | −0.583 | −0.587 | −0.591 | −0.593 | −0.594 | −0.593 | −0.591 | −0.587 | −0.581 | −0.574 | −0.565 |
| −0.628 | −0.622 | −0.615 | −0.608 | −0.600 | −0.591 | −0.582 | −0.571 | −0.561 | −0.549 | −0.537 | −0.524 | −0.510 | −0.496 | −0.481 | −0.464 | −0.447 |
| | | | | | | | | | | | | | | | | |
| −0.542 | −0.541 | −0.540 | −0.538 | −0.535 | −0.532 | −0.528 | −0.523 | −0.518 | −0.511 | −0.504 | −0.496 | −0.488 | −0.479 | −0.469 | −0.457 | −0.445 |
| −1.042 | −1.029 | −1.015 | −1.001 | −0.986 | −0.970 | −0.954 | −0.938 | −0.921 | −0.903 | −0.885 | −0.867 | −0.847 | −0.828 | −0.807 | −0.786 | −0.765 |

| | | | | | | | | | | | | | | | | |
|---|---|---|---|---|---|---|---|---|---|---|---|---|---|---|---|---|
| −0.098 | −0.097 | −0.095 | −0.093 | −0.092 | −0.090 | −0.088 | −0.087 | −0.085 | −0.083 | −0.082 | −0.080 | −0.078 | −0.077 | −0.075 | −0.073 | −0.072 |
| | | | | | | | | | | | | | | | | |
| −0.554 | −0.542 | −0.528 | −0.513 | −0.496 | −0.477 | −0.456 | −0.434 | −0.410 | −0.384 | −0.356 | −0.327 | −0.297 | −0.264 | −0.230 | −0.195 | −0.158 |
| −0.429 | −0.410 | −0.391 | −0.370 | −0.349 | −0.328 | −0.306 | −0.283 | −0.261 | −0.237 | −0.214 | −0.189 | −0.165 | −0.140 | −0.115 | −0.088 | −0.062 |
| | | | | | | | | | | | | | | | | |
| −0.432 | −0.418 | −0.402 | −0.385 | −0.368 | −0.349 | −0.329 | −0.308 | −0.287 | −0.265 | −0.242 | −0.218 | −0.194 | −0.169 | −0.143 | −0.116 | −0.089 |
| −0.742 | −0.720 | −0.697 | −0.673 | −0.649 | −0.625 | −0.600 | −0.575 | −0.550 | −0.524 | −0.497 | −0.470 | −0.443 | −0.415 | −0.387 | −0.358 | −0.329 |

| | | | | | | | | | | | | | | | | |
|---|---|---|---|---|---|---|---|---|---|---|---|---|---|---|---|---|
| −0.070 | −0.068 | −0.067 | −0.065 | −0.063 | −0.062 | −0.060 | −0.058 | −0.057 | −0.055 | −0.053 | −0.052 | −0.050 | −0.048 | −0.047 | −0.045 | −0.043 |
| | | | | | | | | | | | | | | | | |
| −0.120 | −0.080 | −0.038 | 0.004 | 0.048 | 0.093 | 0.139 | 0.186 | 0.233 | 0.281 | 0.330 | 0.379 | 0.429 | 0.478 | 0.529 | 0.579 | 0.630 |
| −0.034 | −0.006 | 0.024 | 0.054 | 0.085 | 0.116 | 0.148 | 0.180 | 0.213 | 0.246 | 0.280 | 0.314 | 0.348 | 0.382 | 0.416 | 0.450 | 0.484 |
| | | | | | | | | | | | | | | | | |
| −0.061 | −0.032 | −0.001 | 0.029 | 0.062 | 0.094 | 0.128 | 0.163 | 0.199 | 0.234 | 0.271 | 0.307 | 0.344 | 0.381 | 0.417 | 0.454 | 0.491 |
| −0.298 | −0.268 | −0.236 | −0.204 | −0.171 | −0.137 | −0.104 | −0.069 | −0.035 | 0.000 | 0.035 | 0.070 | 0.105 | 0.140 | 0.174 | 0.209 | 0.243 |

| | | | | | | | | | | | | | | | | |
|---|---|---|---|---|---|---|---|---|---|---|---|---|---|---|---|---|
| −0.042 | −0.040 | −0.038 | −0.037 | −0.035 | −0.033 | −0.032 | −0.030 | −0.028 | −0.027 | −0.025 | −0.023 | −0.022 | −0.020 | −0.018 | −0.017 | −0.015 |
| 0.681 | 0.732 | 0.783 | 0.835 | 0.886 | 0.937 | 0.988 | 1.038 | 1.089 | 1.138 | 1.188 | 1.237 | 1.285 | 1.332 | 1.378 | 1.423 | 1.467 |
| 0.518 | 0.551 | 0.583 | 0.615 | 0.646 | 0.677 | 0.707 | 0.736 | 0.764 | 0.792 | 0.818 | 0.844 | 0.868 | 0.891 | 0.912 | 0.931 | 0.949 |
| 0.528 | 0.565 | 0.601 | 0.638 | 0.675 | 0.712 | 0.749 | 0.787 | 0.825 | 0.863 | 0.901 | 0.939 | 0.976 | 1.013 | 1.049 | 1.083 | 1.117 |
| 0.276 | 0.309 | 0.341 | 0.371 | 0.401 | 0.430 | 0.458 | 0.484 | 0.509 | 0.533 | 0.556 | 0.578 | 0.598 | 0.618 | 0.635 | 0.652 | 0.666 |

| | | | | | | | | | | | | | | | | |
|---|---|---|---|---|---|---|---|---|---|---|---|---|---|---|---|---|
| −0.013 | −0.012 | −0.010 | −0.008 | −0.007 | −0.005 | −0.003 | −0.002 | 0.000 | 0.002 | 0.003 | 0.005 | 0.007 | 0.008 | 0.010 | 0.012 | 0.013 |
| 1.509 | 1.549 | 1.589 | 1.627 | 1.663 | 1.698 | 1.731 | 1.763 | 1.792 | 1.821 | 1.848 | 1.873 | 1.898 | 1.922 | 1.946 | 1.968 | 1.990 |
| 0.964 | 0.978 | 0.990 | 1.001 | 1.009 | 1.016 | 1.021 | 1.024 | 1.025 | 1.024 | 1.021 | 1.017 | 1.011 | 1.004 | 0.995 | 0.985 | 0.974 |
| 1.149 | 1.180 | 1.209 | 1.237 | 1.263 | 1.288 | 1.312 | 1.335 | 1.356 | 1.377 | 1.397 | 1.417 | 1.436 | 1.454 | 1.472 | 1.489 | 1.506 |
| 0.680 | 0.692 | 0.702 | 0.711 | 0.718 | 0.724 | 0.728 | 0.731 | 0.733 | 0.732 | 0.731 | 0.727 | 0.723 | 0.717 | 0.710 | 0.702 | 0.693 |

| | | | | | | | | | | | | | | | | |
|---|---|---|---|---|---|---|---|---|---|---|---|---|---|---|---|---|
| 0.015 | 0.017 | 0.018 | 0.020 | 0.022 | 0.023 | 0.025 | 0.027 | 0.028 | 0.030 | 0.032 | 0.033 | 0.035 | 0.037 | 0.038 | 0.040 | 0.042 |
| | | | | | | | | | | | | | | | | |
| 2.011 | 2.032 | 2.052 | 2.072 | 2.090 | 2.107 | 2.123 | 2.139 | 2.154 | 2.168 | 2.181 | 2.194 | 2.206 | 2.218 | 2.229 | 2.240 | 2.250 |
| 0.962 | 0.948 | 0.935 | 0.920 | 0.905 | 0.890 | 0.875 | 0.860 | 0.846 | 0.831 | 0.817 | 0.803 | 0.789 | 0.775 | 0.761 | 0.747 | 0.734 |
| | | | | | | | | | | | | | | | | |
| 1.521 | 1.536 | 1.549 | 1.561 | 1.572 | 1.582 | 1.590 | 1.597 | 1.603 | 1.608 | 1.612 | 1.615 | 1.618 | 1.620 | 1.622 | 1.623 | 1.625 |
| 0.683 | 0.672 | 0.660 | 0.648 | 0.635 | 0.621 | 0.608 | 0.594 | 0.580 | 0.565 | 0.552 | 0.538 | 0.525 | 0.513 | 0.501 | 0.490 | 0.479 |

| | | | | | | | | | | | | | | | | |
|---|---|---|---|---|---|---|---|---|---|---|---|---|---|---|---|---|
| 0.043 | 0.045 | 0.047 | 0.048 | 0.050 | 0.052 | 0.053 | 0.055 | 0.057 | 0.058 | 0.060 | 0.062 | 0.063 | 0.065 | 0.067 | 0.068 | 0.070 |
| | | | | | | | | | | | | | | | | |
| 2.259 | 2.267 | 2.275 | 2.283 | 2.290 | 2.297 | 2.303 | 2.310 | 2.315 | 2.320 | 2.325 | 2.330 | 2.333 | 2.337 | 2.340 | 2.343 | 2.345 |
| 0.720 | 0.707 | 0.695 | 0.682 | 0.670 | 0.658 | 0.648 | 0.638 | 0.629 | 0.621 | 0.614 | 0.608 | 0.604 | 0.601 | 0.600 | 0.600 | 0.601 |
| | | | | | | | | | | | | | | | | |
| 1.626 | 1.628 | 1.629 | 1.629 | 1.629 | 1.628 | 1.627 | 1.624 | 1.621 | 1.617 | 1.612 | 1.607 | 1.600 | 1.593 | 1.585 | 1.576 | 1.565 |
| 0.470 | 0.461 | 0.453 | 0.446 | 0.439 | 0.434 | 0.430 | 0.426 | 0.424 | 0.423 | 0.423 | 0.425 | 0.428 | 0.433 | 0.440 | 0.448 | 0.458 |

| | | | | | | | | | | | | | | | | |
|---|---|---|---|---|---|---|---|---|---|---|---|---|---|---|---|---|
| 0.072 | 0.073 | 0.075 | 0.077 | 0.078 | 0.080 | 0.082 | 0.083 | 0.085 | 0.087 | 0.088 | 0.090 | 0.092 | 0.093 | 0.095 | 0.097 | 0.098 |
| | | | | | | | | | | | | | | | | |
| 2.347 | 2.347 | 2.348 | 2.348 | 2.348 | 2.347 | 2.346 | 2.345 | 2.344 | 2.343 | 2.342 | 2.341 | 2.340 | 2.340 | 2.339 | 2.339 | 2.338 |
| 0.604 | 0.608 | 0.613 | 0.620 | 0.627 | 0.637 | 0.647 | 0.659 | 0.672 | 0.686 | 0.702 | 0.718 | 0.735 | 0.754 | 0.773 | 0.794 | 0.815 |
| | | | | | | | | | | | | | | | | |
| 1.554 | 1.541 | 1.527 | 1.511 | 1.494 | 1.477 | 1.458 | 1.439 | 1.420 | 1.400 | 1.380 | 1.361 | 1.341 | 1.321 | 1.302 | 1.282 | 1.263 |
| 0.469 | 0.482 | 0.496 | 0.510 | 0.526 | 0.543 | 0.561 | 0.581 | 0.600 | 0.621 | 0.643 | 0.665 | 0.688 | 0.711 | 0.735 | 0.759 | 0.784 |

| | | | | | | | | | | | | | | | | |
|---|---|---|---|---|---|---|---|---|---|---|---|---|---|---|---|---|
| 0.100 | 0.102 | 0.103 | 0.105 | 0.107 | 0.108 | 0.110 | 0.112 | 0.113 | 0.115 | 0.117 | 0.118 | 0.120 | 0.122 | 0.123 | 0.125 | 0.127 |
| | | | | | | | | | | | | | | | | |
| 2.338 | 2.338 | 2.338 | 2.338 | 2.339 | 2.339 | 2.340 | 2.340 | 2.342 | 2.343 | 2.345 | 2.346 | 2.348 | 2.349 | 2.350 | 2.351 | 2.352 |
| 0.837 | 0.861 | 0.884 | 0.909 | 0.934 | 0.960 | 0.986 | 1.013 | 1.040 | 1.068 | 1.096 | 1.124 | 1.153 | 1.183 | 1.212 | 1.241 | 1.271 |
| | | | | | | | | | | | | | | | | |
| 1.243 | 1.224 | 1.206 | 1.188 | 1.171 | 1.155 | 1.140 | 1.126 | 1.113 | 1.101 | 1.090 | 1.079 | 1.070 | 1.061 | 1.054 | 1.048 | 1.042 |
| 0.808 | 0.833 | 0.859 | 0.884 | 0.909 | 0.934 | 0.960 | 0.986 | 1.012 | 1.039 | 1.066 | 1.093 | 1.120 | 1.147 | 1.174 | 1.201 | 1.228 |

| | | | | | | | | | | | | | | | | |
|---|---|---|---|---|---|---|---|---|---|---|---|---|---|---|---|---|
| 0.128 | 0.130 | 0.132 | 0.133 | 0.135 | 0.137 | 0.138 | 0.140 | 0.142 | 0.143 | 0.145 | 0.147 | 0.148 | 0.150 | 0.152 | 0.153 | 0.155 |
| 2.351 | 2.350 | 2.349 | 2.347 | 2.344 | 2.341 | 2.337 | 2.332 | 2.327 | 2.321 | 2.315 | 2.307 | 2.300 | 2.291 | 2.281 | 2.269 | 2.257 |
| 1.300 | 1.328 | 1.357 | 1.385 | 1.412 | 1.439 | 1.466 | 1.491 | 1.515 | 1.539 | 1.561 | 1.582 | 1.602 | 1.621 | 1.639 | 1.655 | 1.669 |
| 1.038 | 1.035 | 1.032 | 1.030 | 1.028 | 1.027 | 1.026 | 1.026 | 1.026 | 1.026 | 1.026 | 1.025 | 1.024 | 1.022 | 1.020 | 1.017 | 1.012 |
| 1.255 | 1.282 | 1.309 | 1.335 | 1.361 | 1.387 | 1.413 | 1.438 | 1.463 | 1.488 | 1.512 | 1.535 | 1.558 | 1.580 | 1.602 | 1.622 | 1.642 |

| | | | | | | | | | | | | | | | | |
|---|---|---|---|---|---|---|---|---|---|---|---|---|---|---|---|---|
| 0.157 | 0.158 | 0.160 | 0.162 | 0.163 | 0.165 | 0.166 | 0.168 | 0.170 | 0.171 | 0.173 | 0.175 | 0.176 | 0.178 | 0.180 | 0.181 | 0.183 |
| 2.243 | 2.228 | 2.212 | 2.194 | 2.175 | 2.154 | 2.132 | 2.108 | 2.083 | 2.056 | 2.027 | 1.997 | 1.965 | 1.932 | 1.897 | 1.860 | 1.821 |
| 1.683 | 1.694 | 1.703 | 1.710 | 1.716 | 1.719 | 1.719 | 1.718 | 1.714 | 1.707 | 1.699 | 1.687 | 1.673 | 1.656 | 1.637 | 1.615 | 1.590 |
| 1.007 | 1.001 | 0.994 | 0.985 | 0.976 | 0.965 | 0.953 | 0.940 | 0.926 | 0.910 | 0.894 | 0.876 | 0.857 | 0.837 | 0.816 | 0.794 | 0.770 |
| 1.661 | 1.679 | 1.696 | 1.712 | 1.728 | 1.742 | 1.756 | 1.768 | 1.779 | 1.789 | 1.797 | 1.804 | 1.808 | 1.811 | 1.812 | 1.810 | 1.807 |

| | | | | | | | | | | | | | | | | |
|---|---|---|---|---|---|---|---|---|---|---|---|---|---|---|---|---|
| 0.185 | 0.186 | 0.188 | 0.190 | 0.191 | 0.193 | 0.195 | 0.196 | 0.198 | 0.200 | 0.201 | 0.203 | 0.205 | 0.206 | 0.208 | 0.210 | 0.211 |
| | | | | | | | | | | | | | | | | |
| 1.781 | 1.739 | 1.695 | 1.650 | 1.602 | 1.554 | 1.504 | 1.453 | 1.400 | 1.345 | 1.289 | 1.232 | 1.172 | 1.112 | 1.049 | 0.985 | 0.920 |
| 1.563 | 1.533 | 1.501 | 1.466 | 1.429 | 1.390 | 1.349 | 1.305 | 1.259 | 1.210 | 1.160 | 1.107 | 1.053 | 0.997 | 0.938 | 0.878 | 0.816 |
| | | | | | | | | | | | | | | | | |
| 0.744 | 0.717 | 0.688 | 0.657 | 0.626 | 0.592 | 0.557 | 0.521 | 0.483 | 0.442 | 0.401 | 0.358 | 0.313 | 0.267 | 0.220 | 0.171 | 0.121 |
| 1.801 | 1.793 | 1.782 | 1.769 | 1.754 | 1.735 | 1.715 | 1.692 | 1.666 | 1.638 | 1.608 | 1.575 | 1.540 | 1.503 | 1.463 | 1.421 | 1.376 |

| | | | | | | | | | | | | | | | | |
|---|---|---|---|---|---|---|---|---|---|---|---|---|---|---|---|---|
| 0.213 | 0.215 | 0.216 | 0.218 | 0.220 | 0.221 | 0.223 | 0.225 | 0.226 | 0.228 | 0.230 | 0.231 | 0.233 | 0.235 | 0.236 | 0.238 | 0.240 |
| | | | | | | | | | | | | | | | | |
| 0.853 | 0.785 | 0.716 | 0.645 | 0.575 | 0.503 | 0.431 | 0.358 | 0.285 | 0.212 | 0.138 | 0.064 | −0.010 | −0.084 | −0.159 | −0.233 | −0.307 |
| 0.753 | 0.688 | 0.621 | 0.553 | 0.483 | 0.411 | 0.337 | 0.262 | 0.186 | 0.108 | 0.029 | −0.052 | −0.134 | −0.217 | −0.301 | −0.386 | −0.472 |
| | | | | | | | | | | | | | | | | |
| 0.070 | 0.018 | −0.035 | −0.089 | −0.145 | −0.201 | −0.258 | −0.316 | −0.374 | −0.433 | −0.492 | −0.552 | −0.613 | −0.674 | −0.735 | −0.798 | −0.860 |
| 1.329 | 1.280 | 1.229 | 1.176 | 1.122 | 1.065 | 1.007 | 0.948 | 0.887 | 0.824 | 0.760 | 0.694 | 0.628 | 0.560 | 0.491 | 0.422 | 0.351 |

| | | | | | | | | | | | | | | | | |
|---|---|---|---|---|---|---|---|---|---|---|---|---|---|---|---|---|
| 0.241 | 0.243 | 0.245 | 0.246 | 0.248 | 0.250 | 0.251 | 0.253 | 0.255 | 0.256 | 0.258 | 0.260 | 0.261 | 0.263 | 0.265 | 0.266 | 0.268 |
| −0.381 | −0.455 | −0.529 | −0.603 | −0.677 | −0.752 | −0.827 | −0.902 | −0.976 | −1.051 | −1.125 | −1.199 | −1.272 | −1.346 | −1.418 | −1.490 | −1.561 |
| −0.559 | −0.646 | −0.734 | −0.823 | −0.913 | −1.003 | −1.094 | −1.186 | −1.278 | −1.371 | −1.463 | −1.556 | −1.649 | −1.743 | −1.836 | −1.930 | −2.024 |
| −0.923 | −0.987 | −1.051 | −1.115 | −1.180 | −1.245 | −1.311 | −1.376 | −1.442 | −1.507 | −1.572 | −1.636 | −1.701 | −1.766 | −1.830 | −1.895 | −1.959 |
| 0.280 | 0.208 | 0.136 | 0.063 | −0.010 | −0.084 | −0.158 | −0.232 | −0.306 | −0.380 | −0.455 | −0.529 | −0.604 | −0.678 | −0.752 | −0.826 | −0.900 |

| | | | | | | | | | | | | | | | | |
|---|---|---|---|---|---|---|---|---|---|---|---|---|---|---|---|---|
| 0.270 | 0.271 | 0.273 | 0.275 | 0.276 | 0.278 | 0.280 | 0.281 | 0.283 | 0.285 | 0.286 | 0.288 | 0.290 | 0.291 | 0.293 | 0.295 | 0.296 |
| | | | | | | | | | | | | | | | | |
| −1.631 | −1.701 | −1.770 | −1.838 | −1.905 | −1.971 | −2.036 | −2.101 | −2.164 | −2.227 | −2.289 | −2.350 | −2.410 | −2.470 | −2.529 | −2.587 | −2.644 |
| −2.119 | −2.214 | −2.308 | −2.403 | −2.498 | −2.591 | −2.685 | −2.777 | −2.869 | −2.960 | −3.050 | −3.138 | −3.226 | −3.312 | −3.397 | −3.480 | −3.562 |
| | | | | | | | | | | | | | | | | |
| −2.024 | −2.088 | −2.153 | −2.217 | −2.282 | −2.347 | −2.412 | −2.477 | −2.542 | −2.607 | −2.672 | −2.737 | −2.803 | −2.869 | −2.936 | −3.002 | −3.069 |
| −0.974 | −1.046 | −1.119 | −1.190 | −1.261 | −1.330 | −1.398 | −1.465 | −1.530 | −1.593 | −1.656 | −1.716 | −1.776 | −1.833 | −1.890 | −1.944 | −1.998 |

| | | | | | | | | | | | | | | | | |
|---|---|---|---|---|---|---|---|---|---|---|---|---|---|---|---|---|
| 0.298 | 0.300 | 0.301 | 0.303 | 0.305 | 0.306 | 0.308 | 0.310 | 0.311 | 0.313 | 0.315 | 0.316 | 0.318 | 0.320 | 0.321 | 0.323 | 0.325 |
| −2.701 | −2.757 | −2.812 | −2.866 | −2.919 | −2.970 | −3.020 | −3.068 | −3.114 | −3.158 | −3.201 | −3.242 | −3.283 | −3.322 | −3.360 | −3.396 | −3.432 |
| −3.643 | −3.721 | −3.798 | −3.874 | −3.947 | −4.019 | −4.089 | −4.158 | −4.225 | −4.290 | −4.354 | −4.417 | −4.479 | −4.539 | −4.598 | −4.655 | −4.712 |
| −3.136 | −3.203 | −3.271 | −3.338 | −3.406 | −3.473 | −3.541 | −3.607 | −3.673 | −3.738 | −3.802 | −3.865 | −3.927 | −3.988 | −4.048 | −4.106 | −4.164 |
| −2.049 | −2.099 | −2.147 | −2.194 | −2.239 | −2.283 | −2.326 | −2.367 | −2.407 | −2.445 | −2.483 | −2.519 | −2.555 | −2.589 | −2.622 | −2.654 | −2.686 |

| | | | | | | | | | | | | | | | | |
|---|---|---|---|---|---|---|---|---|---|---|---|---|---|---|---|---|
| 0.326 | 0.328 | 0.330 | 0.331 | 0.333 | 0.335 | 0.336 | 0.338 | 0.340 | 0.341 | 0.343 | 0.345 | 0.346 | 0.348 | 0.350 | 0.351 | 0.353 |
| −3.467 | −3.501 | −3.533 | −3.565 | −3.595 | −3.625 | −3.653 | −3.681 | −3.707 | −3.733 | −3.757 | −3.781 | −3.804 | −3.827 | −3.850 | −3.873 | −3.896 |
| −4.768 | −4.822 | −4.876 | −4.929 | −4.981 | −5.032 | −5.083 | −5.132 | −5.181 | −5.229 | −5.276 | −5.322 | −5.367 | −5.412 | −5.455 | −5.497 | −5.539 |
| −4.219 | −4.273 | −4.326 | −4.376 | −4.425 | −4.471 | −4.517 | −4.560 | −4.602 | −4.643 | −4.683 | −4.722 | −4.760 | −4.798 | −4.836 | −4.873 | −4.909 |
| −2.716 | −2.746 | −2.774 | −2.802 | −2.829 | −2.855 | −2.880 | −2.905 | −2.929 | −2.952 | −2.975 | −2.997 | −3.019 | −3.040 | −3.061 | −3.082 | −3.102 |

| | | | | | | | | | | | | | | | | |
|---|---|---|---|---|---|---|---|---|---|---|---|---|---|---|---|---|
| 0.355 | 0.356 | 0.358 | 0.360 | 0.361 | 0.363 | 0.365 | 0.366 | 0.368 | 0.370 | 0.371 | 0.373 | 0.375 | 0.376 | 0.378 | 0.380 | 0.381 |
| −3.920 | −3.945 | −3.969 | −3.995 | −4.021 | −4.049 | −4.077 | −4.106 | −4.136 | −4.168 | −4.199 | −4.232 | −4.264 | −4.299 | −4.333 | −4.368 | −4.404 |
| −5.579 | −5.618 | −5.655 | −5.691 | −5.726 | −5.759 | −5.791 | −5.821 | −5.850 | −5.877 | −5.902 | −5.926 | −5.948 | −5.969 | −5.988 | −6.007 | −6.024 |
| −4.946 | −4.982 | −5.018 | −5.055 | −5.092 | −5.130 | −5.169 | −5.208 | −5.248 | −5.290 | −5.332 | −5.375 | −5.419 | −5.464 | −5.510 | −5.557 | −5.605 |
| −3.123 | −3.144 | −3.164 | −3.185 | −3.205 | −3.226 | −3.247 | −3.268 | −3.289 | −3.310 | −3.331 | −3.351 | −3.371 | −3.391 | −3.411 | −3.430 | −3.449 |

| | | | | | | | | | | | | | | | | |
|---|---|---|---|---|---|---|---|---|---|---|---|---|---|---|---|---|
| 0.383 | 0.385 | 0.386 | 0.388 | 0.390 | 0.391 | 0.393 | 0.395 | 0.396 | 0.398 | 0.400 | 0.401 | 0.403 | 0.405 | 0.406 | 0.408 | 0.410 |
| | | | | | | | | | | | | | | | | |
| −4.441 | −4.478 | −4.516 | −4.555 | −4.595 | −4.636 | −4.678 | −4.721 | −4.766 | −4.811 | −4.858 | −4.906 | −4.956 | −5.006 | −5.057 | −5.110 | −5.163 |
| −6.041 | −6.056 | −6.070 | −6.083 | −6.095 | −6.106 | −6.115 | −6.123 | −6.130 | −6.136 | −6.140 | −6.144 | −6.146 | −6.147 | −6.147 | −6.146 | −6.144 |
| | | | | | | | | | | | | | | | | |
| −5.654 | −5.704 | −5.755 | −5.807 | −5.860 | −5.913 | −5.968 | −6.023 | −6.079 | −6.136 | −6.194 | −6.253 | −6.311 | −6.371 | −6.430 | −6.490 | −6.551 |
| −3.467 | −3.484 | −3.501 | −3.518 | −3.534 | −3.550 | −3.565 | −3.580 | −3.594 | −3.608 | −3.622 | −3.635 | −3.648 | −3.661 | −3.673 | −3.684 | −3.694 |

| | | | | | | | | | | | | | | | | |
|---|---|---|---|---|---|---|---|---|---|---|---|---|---|---|---|---|
| 0.411 | 0.413 | 0.415 | 0.416 | 0.418 | 0.420 | 0.421 | 0.423 | 0.425 | 0.426 | 0.428 | 0.430 | 0.431 | 0.433 | 0.435 | 0.436 | 0.438 |
| | | | | | | | | | | | | | | | | |
| −5.217 | −5.272 | −5.328 | −5.384 | −5.441 | −5.499 | −5.558 | −5.617 | −5.677 | −5.737 | −5.798 | −5.859 | −5.921 | −5.983 | −6.045 | −6.107 | −6.169 |
| −6.142 | −6.138 | −6.134 | −6.128 | −6.123 | −6.115 | −6.108 | −6.100 | −6.091 | −6.081 | −6.072 | −6.061 | −6.051 | −6.040 | −6.030 | −6.018 | −6.007 |
| | | | | | | | | | | | | | | | | |
| −6.612 | −6.673 | −6.734 | −6.796 | −6.858 | −6.920 | −6.983 | −7.046 | −7.109 | −7.172 | −7.235 | −7.297 | −7.360 | −7.422 | −7.484 | −7.546 | −7.607 |
| −3.704 | −3.712 | −3.720 | −3.726 | −3.732 | −3.736 | −3.740 | −3.742 | −3.743 | −3.743 | −3.742 | −3.739 | −3.735 | −3.730 | −3.723 | −3.716 | −3.707 |

| | | | | | | | | | | | | | | | | |
|---|---|---|---|---|---|---|---|---|---|---|---|---|---|---|---|---|
| 0.440 | 0.441 | 0.443 | 0.445 | 0.446 | 0.448 | 0.450 | 0.451 | 0.453 | 0.455 | 0.456 | 0.458 | 0.460 | 0.461 | 0.463 | 0.465 | 0.466 |
| | | | | | | | | | | | | | | | | |
| −6.231 | −6.292 | −6.352 | −6.412 | −6.471 | −6.530 | −6.588 | −6.646 | −6.703 | −6.760 | −6.817 | −6.873 | −6.928 | −6.982 | −7.036 | −7.089 | −7.142 |
| −5.995 | −5.983 | −5.971 | −5.958 | −5.945 | −5.932 | −5.918 | −5.904 | −5.889 | −5.874 | −5.859 | −5.844 | −5.828 | −5.812 | −5.796 | −5.779 | −5.762 |
| | | | | | | | | | | | | | | | | |
| −7.669 | −7.729 | −7.790 | −7.851 | −7.911 | −7.972 | −8.032 | −8.092 | −8.152 | −8.212 | −8.272 | −8.331 | −8.391 | −8.449 | −8.507 | −8.564 | −8.620 |
| −3.697 | −3.686 | −3.674 | −3.661 | −3.647 | −3.632 | −3.617 | −3.601 | −3.584 | −3.566 | −3.547 | −3.528 | −3.508 | −3.488 | −3.467 | −3.445 | −3.422 |

| | | | | | | | | | | | | | | | | |
|---|---|---|---|---|---|---|---|---|---|---|---|---|---|---|---|---|
| 0.468 | 0.470 | 0.471 | 0.473 | 0.475 | 0.476 | 0.478 | 0.480 | 0.481 | 0.483 | 0.485 | 0.486 | 0.488 | 0.489 | 0.491 | 0.493 | 0.494 |
| | | | | | | | | | | | | | | | | |
| −7.194 | −7.245 | −7.296 | −7.345 | −7.392 | −7.439 | −7.483 | −7.526 | −7.567 | −7.607 | −7.646 | −7.684 | −7.722 | −7.758 | −7.793 | −7.828 | −7.862 |
| −5.744 | −5.726 | −5.708 | −5.690 | −5.671 | −5.653 | −5.634 | −5.615 | −5.597 | −5.578 | −5.560 | −5.542 | −5.524 | −5.506 | −5.488 | −5.471 | −5.453 |
| | | | | | | | | | | | | | | | | |
| −8.674 | −8.727 | −8.778 | −8.828 | −8.876 | −8.923 | −8.968 | −9.011 | −9.053 | −9.093 | −9.132 | −9.168 | −9.203 | −9.236 | −9.268 | −9.299 | −9.329 |
| −3.399 | −3.376 | −3.352 | −3.327 | −3.303 | −3.278 | −3.252 | −3.227 | −3.201 | −3.176 | −3.150 | −3.125 | −3.099 | −3.074 | −3.048 | −3.023 | −2.998 |

| | | | | | | | | | | | | | | | | |
|---|---|---|---|---|---|---|---|---|---|---|---|---|---|---|---|---|
| 0.496 | 0.498 | 0.499 | 0.501 | 0.503 | 0.504 | 0.506 | 0.508 | 0.509 | 0.511 | 0.513 | 0.514 | 0.516 | 0.518 | 0.519 | 0.521 | 0.523 |
| −7.895 | −7.928 | −7.959 | −7.990 | −8.019 | −8.048 | −8.075 | −8.101 | −8.127 | −8.151 | −8.173 | −8.195 | −8.215 | −8.234 | −8.252 | −8.269 | −8.285 |
| −5.436 | −5.419 | −5.402 | −5.385 | −5.368 | −5.352 | −5.335 | −5.319 | −5.303 | −5.288 | −5.273 | −5.259 | −5.245 | −5.231 | −5.218 | −5.205 | −5.192 |
| −9.357 | −9.384 | −9.409 | −9.433 | −9.456 | −9.476 | −9.495 | −9.512 | −9.527 | −9.541 | −9.554 | −9.565 | −9.574 | −9.582 | −9.589 | −9.594 | −9.598 |
| −2.972 | −2.947 | −2.922 | −2.898 | −2.874 | −2.850 | −2.826 | −2.803 | −2.780 | −2.756 | −2.734 | −2.711 | −2.688 | −2.666 | −2.643 | −2.621 | −2.599 |

| | | | | | | | | | | | | | | | | |
|---|---|---|---|---|---|---|---|---|---|---|---|---|---|---|---|---|
| 0.524 | 0.526 | 0.528 | 0.529 | 0.531 | 0.533 | 0.534 | 0.536 | 0.538 | 0.539 | 0.541 | 0.543 | 0.544 | 0.546 | 0.548 | 0.549 | 0.551 |
| −8.300 | −8.314 | −8.327 | −8.340 | −8.351 | −8.362 | −8.371 | −8.380 | −8.389 | −8.397 | −8.404 | −8.410 | −8.416 | −8.421 | −8.425 | −8.429 | −8.433 |
| −5.179 | −5.167 | −5.155 | −5.144 | −5.132 | −5.120 | −5.109 | −5.097 | −5.085 | −5.073 | −5.061 | −5.049 | −5.036 | −5.023 | −5.010 | −4.997 | −4.983 |
| −9.600 | −9.602 | −9.602 | −9.601 | −9.599 | −9.596 | −9.592 | −9.587 | −9.581 | −9.574 | −9.565 | −9.556 | −9.546 | −9.536 | −9.526 | −9.515 | −9.504 |
| −2.578 | −2.557 | −2.536 | −2.516 | −2.496 | −2.476 | −2.457 | −2.439 | −2.420 | −2.403 | −2.385 | −2.368 | −2.350 | −2.333 | −2.316 | −2.299 | −2.281 |

| | | | | | | | | | | | | | | | | |
|---|---|---|---|---|---|---|---|---|---|---|---|---|---|---|---|---|
| 0.553 | 0.554 | 0.556 | 0.558 | 0.559 | 0.561 | 0.563 | 0.564 | 0.566 | 0.568 | 0.569 | 0.571 | 0.573 | 0.574 | 0.576 | 0.578 | 0.579 |
| −8.437 | −8.441 | −8.445 | −8.450 | −8.454 | −8.458 | −8.462 | −8.467 | −8.471 | −8.475 | −8.479 | −8.482 | −8.485 | −8.488 | −8.490 | −8.492 | −8.494 |
| −4.969 | −4.954 | −4.939 | −4.924 | −4.907 | −4.890 | −4.873 | −4.855 | −4.836 | −4.817 | −4.798 | −4.778 | −4.757 | −4.736 | −4.714 | −4.691 | −4.668 |
| −9.492 | −9.481 | −9.470 | −9.458 | −9.446 | −9.435 | −9.423 | −9.410 | −9.398 | −9.385 | −9.371 | −9.357 | −9.343 | −9.329 | −9.314 | −9.299 | −9.284 |
| −2.264 | −2.246 | −2.229 | −2.211 | −2.193 | −2.176 | −2.158 | −2.141 | −2.124 | −2.106 | −2.090 | −2.073 | −2.057 | −2.041 | −2.025 | −2.010 | −1.995 |

| | | | | | | | | | | | | | | | | |
|---|---|---|---|---|---|---|---|---|---|---|---|---|---|---|---|---|
| 0.581 | 0.583 | 0.584 | 0.586 | 0.588 | 0.589 | 0.591 | 0.593 | 0.594 | 0.596 | 0.598 | 0.599 | 0.601 | 0.603 | 0.604 | 0.606 | 0.608 |
| | | | | | | | | | | | | | | | | |
| −8.496 | −8.497 | −8.498 | −8.500 | −8.500 | −8.501 | −8.502 | −8.503 | −8.504 | −8.505 | −8.506 | −8.507 | −8.508 | −8.509 | −8.510 | −8.511 | −8.511 |
| −4.644 | −4.619 | −4.594 | −4.567 | −4.540 | −4.512 | −4.482 | −4.452 | −4.421 | −4.389 | −4.356 | −4.322 | −4.288 | −4.252 | −4.216 | −4.179 | −4.142 |
| | | | | | | | | | | | | | | | | |
| −9.269 | −9.253 | −9.238 | −9.222 | −9.207 | −9.191 | −9.176 | −9.160 | −9.145 | −9.131 | −9.117 | −9.105 | −9.093 | −9.082 | −9.073 | −9.065 | −9.058 |
| −1.980 | −1.966 | −1.953 | −1.940 | −1.927 | −1.915 | −1.903 | −1.890 | −1.878 | −1.866 | −1.853 | −1.840 | −1.827 | −1.813 | −1.798 | −1.783 | −1.767 |

| | | | | | | | | | | | | | | | | |
|---|---|---|---|---|---|---|---|---|---|---|---|---|---|---|---|---|
| 0.609 | 0.611 | 0.613 | 0.614 | 0.616 | 0.618 | 0.619 | 0.621 | 0.623 | 0.624 | 0.626 | 0.628 | 0.629 | 0.631 | 0.633 | 0.634 | 0.636 |
| | | | | | | | | | | | | | | | | |
| −8.511 | −8.511 | −8.510 | −8.510 | −8.509 | −8.508 | −8.507 | −8.505 | −8.503 | −8.501 | −8.499 | −8.496 | −8.493 | −8.490 | −8.486 | −8.482 | −8.477 |
| −4.104 | −4.065 | −4.025 | −3.985 | −3.944 | −3.903 | −3.861 | −3.818 | −3.774 | −3.730 | −3.685 | −3.640 | −3.594 | −3.548 | −3.501 | −3.455 | −3.407 |
| | | | | | | | | | | | | | | | | |
| −9.053 | −9.048 | −9.046 | −9.044 | −9.043 | −9.044 | −9.045 | −9.048 | −9.051 | −9.056 | −9.060 | −9.066 | −9.071 | −9.076 | −9.082 | −9.087 | −9.093 |
| −1.751 | −1.734 | −1.716 | −1.697 | −1.678 | −1.658 | −1.637 | −1.616 | −1.593 | −1.571 | −1.548 | −1.526 | −1.502 | −1.478 | −1.454 | −1.429 | −1.404 |

| | | | | | | | | | | | | | | | | |
|---|---|---|---|---|---|---|---|---|---|---|---|---|---|---|---|---|
| 0.638 | 0.639 | 0.641 | 0.643 | 0.644 | 0.646 | 0.648 | 0.649 | 0.651 | 0.653 | 0.654 | 0.656 | 0.658 | 0.659 | 0.661 | 0.663 | 0.664 |
| −8.473 | −8.468 | −8.463 | −8.458 | −8.452 | −8.446 | −8.439 | −8.432 | −8.425 | −8.417 | −8.409 | −8.401 | −8.393 | −8.384 | −8.374 | −8.364 | −8.353 |
| −3.360 | −3.313 | −3.265 | −3.217 | −3.168 | −3.119 | −3.069 | −3.018 | −2.967 | −2.915 | −2.863 | −2.809 | −2.755 | −2.700 | −2.643 | −2.586 | −2.527 |
| −9.098 | −9.104 | −9.109 | −9.114 | −9.120 | −9.125 | −9.130 | −9.134 | −9.139 | −9.143 | −9.148 | −9.152 | −9.156 | −9.160 | −9.164 | −9.168 | −9.171 |
| −1.379 | −1.354 | −1.329 | −1.303 | −1.277 | −1.251 | −1.224 | −1.197 | −1.170 | −1.142 | −1.114 | −1.085 | −1.056 | −1.026 | −0.996 | −0.965 | −0.933 |

| | | | | | | | | | | | | | | | | |
|---|---|---|---|---|---|---|---|---|---|---|---|---|---|---|---|---|
| 0.666 | 0.668 | 0.669 | 0.671 | 0.673 | 0.674 | 0.676 | 0.678 | 0.679 | 0.681 | 0.683 | 0.684 | 0.686 | 0.688 | 0.689 | 0.691 | 0.693 |
| −8.342 | −8.330 | −8.317 | −8.304 | −8.289 | −8.274 | −8.257 | −8.239 | −8.221 | −8.200 | −8.178 | −8.155 | −8.130 | −8.104 | −8.077 | −8.049 | −8.019 |
| −2.467 | −2.406 | −2.343 | −2.280 | −2.216 | −2.150 | −2.084 | −2.017 | −1.950 | −1.881 | −1.812 | −1.741 | −1.670 | −1.598 | −1.526 | −1.452 | −1.378 |
| −9.173 | −9.174 | −9.175 | −9.175 | −9.173 | −9.170 | −9.166 | −9.159 | −9.151 | −9.140 | −9.128 | −9.114 | −9.098 | −9.080 | −9.060 | −9.038 | −9.015 |
| −0.901 | −0.868 | −0.835 | −0.801 | −0.766 | −0.731 | −0.695 | −0.658 | −0.622 | −0.584 | −0.546 | −0.507 | −0.468 | −0.429 | −0.388 | −0.347 | −0.306 |

| | | | | | | | | | | | | | | | | |
|---|---|---|---|---|---|---|---|---|---|---|---|---|---|---|---|---|
| 0.694 | 0.696 | 0.698 | 0.699 | 0.701 | 0.703 | 0.704 | 0.706 | 0.708 | 0.709 | 0.711 | 0.713 | 0.714 | 0.716 | 0.718 | 0.719 | 0.721 |
| | | | | | | | | | | | | | | | | |
| −7.988 | −7.956 | −7.923 | −7.888 | −7.852 | −7.813 | −7.774 | −7.732 | −7.688 | −7.643 | −7.596 | −7.547 | −7.496 | −7.444 | −7.390 | −7.334 | −7.277 |
| −1.304 | −1.229 | −1.154 | −1.079 | −1.003 | −0.928 | −0.852 | −0.776 | −0.699 | −0.623 | −0.547 | −0.471 | −0.395 | −0.318 | −0.243 | −0.167 | −0.092 |
| | | | | | | | | | | | | | | | | |
| −8.990 | −8.964 | −8.936 | −8.905 | −8.873 | −8.839 | −8.803 | −8.764 | −8.724 | −8.681 | −8.636 | −8.588 | −8.539 | −8.487 | −8.433 | −8.378 | −8.320 |
| −0.264 | −0.222 | −0.179 | −0.136 | −0.092 | −0.047 | −0.003 | 0.042 | 0.087 | 0.133 | 0.178 | 0.224 | 0.270 | 0.316 | 0.362 | 0.408 | 0.454 |

| | | | | | | | | | | | | | | | | |
|---|---|---|---|---|---|---|---|---|---|---|---|---|---|---|---|---|
| 0.723 | 0.724 | 0.726 | 0.728 | 0.729 | 0.731 | 0.733 | 0.734 | 0.736 | 0.738 | 0.739 | 0.741 | 0.743 | 0.744 | 0.746 | 0.748 | 0.749 |
| −7.218 | −7.157 | −7.095 | −7.031 | −6.965 | −6.898 | −6.829 | −6.759 | −6.687 | −6.615 | −6.542 | −6.468 | −6.393 | −6.318 | −6.243 | −6.168 | −6.093 |
| −0.016 | 0.059 | 0.134 | 0.208 | 0.283 | 0.357 | 0.431 | 0.505 | 0.578 | 0.651 | 0.723 | 0.794 | 0.865 | 0.935 | 1.004 | 1.072 | 1.139 |
| −8.260 | −8.198 | −8.134 | −8.070 | −8.003 | −7.936 | −7.867 | −7.798 | −7.726 | −7.654 | −7.581 | −7.506 | −7.431 | −7.355 | −7.277 | −7.199 | −7.120 |
| 0.500 | 0.546 | 0.592 | 0.638 | 0.684 | 0.730 | 0.776 | 0.821 | 0.868 | 0.914 | 0.961 | 1.008 | 1.055 | 1.103 | 1.151 | 1.200 | 1.250 |

| | | | | | | | | | | | | | | | | |
|---|---|---|---|---|---|---|---|---|---|---|---|---|---|---|---|---|
| 0.751 | 0.753 | 0.754 | 0.756 | 0.758 | 0.759 | 0.761 | 0.763 | 0.764 | 0.766 | 0.768 | 0.769 | 0.771 | 0.773 | 0.774 | 0.776 | 0.778 |
| | | | | | | | | | | | | | | | | |
| −6.019 | −5.945 | −5.872 | −5.799 | −5.729 | −5.659 | −5.590 | −5.523 | −5.457 | −5.393 | −5.330 | −5.268 | −5.207 | −5.148 | −5.090 | −5.033 | −4.978 |
| 1.204 | 1.269 | 1.332 | 1.393 | 1.453 | 1.512 | 1.570 | 1.626 | 1.680 | 1.734 | 1.786 | 1.837 | 1.886 | 1.934 | 1.980 | 2.025 | 2.069 |
| | | | | | | | | | | | | | | | | |
| −7.040 | −6.959 | −6.879 | −6.799 | −6.720 | −6.641 | −6.563 | −6.485 | −6.408 | −6.333 | −6.258 | −6.185 | −6.113 | −6.042 | −5.974 | −5.907 | −5.841 |
| 1.299 | 1.350 | 1.400 | 1.451 | 1.501 | 1.552 | 1.603 | 1.654 | 1.705 | 1.756 | 1.807 | 1.858 | 1.910 | 1.961 | 2.012 | 2.062 | 2.113 |

| | | | | | | | | | | | | | | | | |
|---|---|---|---|---|---|---|---|---|---|---|---|---|---|---|---|---|
| 0.779 | 0.781 | 0.783 | 0.784 | 0.786 | 0.788 | 0.789 | 0.791 | 0.793 | 0.794 | 0.796 | 0.798 | 0.799 | 0.801 | 0.803 | 0.804 | 0.806 |
| −4.924 | −4.871 | −4.820 | −4.771 | −4.723 | −4.677 | −4.632 | −4.589 | −4.547 | −4.506 | −4.468 | −4.430 | −4.395 | −4.361 | −4.330 | −4.300 | −4.272 |
| 2.111 | 2.152 | 2.192 | 2.230 | 2.268 | 2.304 | 2.340 | 2.374 | 2.407 | 2.439 | 2.470 | 2.499 | 2.527 | 2.554 | 2.579 | 2.603 | 2.625 |
| −5.778 | −5.716 | −5.656 | −5.597 | −5.540 | −5.485 | −5.432 | −5.381 | −5.332 | −5.284 | −5.238 | −5.195 | −5.154 | −5.115 | −5.078 | −5.044 | −5.013 |
| 2.162 | 2.212 | 2.260 | 2.308 | 2.356 | 2.402 | 2.447 | 2.491 | 2.534 | 2.576 | 2.616 | 2.654 | 2.691 | 2.726 | 2.759 | 2.791 | 2.820 |

| | | | | | | | | | | | | | | | | |
|---|---|---|---|---|---|---|---|---|---|---|---|---|---|---|---|---|
| 0.808 | 0.809 | 0.811 | 0.813 | 0.814 | 0.816 | 0.817 | 0.819 | 0.821 | 0.822 | 0.824 | 0.826 | 0.827 | 0.829 | 0.831 | 0.832 | 0.834 |
| −4.246 | −4.223 | −4.201 | −4.182 | −4.166 | −4.151 | −4.139 | −4.130 | −4.123 | −4.119 | −4.117 | −4.117 | −4.119 | −4.123 | −4.129 | −4.137 | −4.148 |
| 2.646 | 2.666 | 2.684 | 2.701 | 2.716 | 2.730 | 2.742 | 2.752 | 2.760 | 2.767 | 2.772 | 2.775 | 2.777 | 2.777 | 2.775 | 2.771 | 2.766 |
| −4.984 | −4.959 | −4.936 | −4.916 | −4.899 | −4.885 | −4.874 | −4.866 | −4.862 | −4.860 | −4.861 | −4.865 | −4.872 | −4.882 | −4.895 | −4.910 | −4.927 |
| 2.847 | 2.873 | 2.897 | 2.919 | 2.939 | 2.956 | 2.972 | 2.986 | 2.997 | 3.007 | 3.014 | 3.019 | 3.022 | 3.023 | 3.022 | 3.019 | 3.014 |

| | | | | | | | | | | | | | | | | |
|---|---|---|---|---|---|---|---|---|---|---|---|---|---|---|---|---|
| 0.836 | 0.837 | 0.839 | 0.841 | 0.842 | 0.844 | 0.846 | 0.847 | 0.849 | 0.851 | 0.852 | 0.854 | 0.856 | 0.857 | 0.859 | 0.861 | 0.862 |
| −4.160 | −4.175 | −4.192 | −4.211 | −4.232 | −4.256 | −4.281 | −4.309 | −4.339 | −4.370 | −4.404 | −4.439 | −4.476 | −4.514 | −4.554 | −4.595 | −4.637 |
| 2.759 | 2.750 | 2.739 | 2.726 | 2.712 | 2.697 | 2.680 | 2.661 | 2.641 | 2.620 | 2.597 | 2.572 | 2.546 | 2.518 | 2.488 | 2.457 | 2.424 |
| −4.948 | −4.970 | −4.996 | −5.023 | −5.054 | −5.086 | −5.121 | −5.157 | −5.196 | −5.236 | −5.278 | −5.322 | −5.368 | −5.416 | −5.466 | −5.518 | −5.572 |
| 3.007 | 2.998 | 2.988 | 2.975 | 2.960 | 2.943 | 2.924 | 2.902 | 2.879 | 2.854 | 2.826 | 2.797 | 2.766 | 2.733 | 2.698 | 2.662 | 2.624 |

| | | | | | | | | | | | | | | | | |
|---|---|---|---|---|---|---|---|---|---|---|---|---|---|---|---|---|
| 0.864 | 0.866 | 0.867 | 0.869 | 0.871 | 0.872 | 0.874 | 0.876 | 0.877 | 0.879 | 0.881 | 0.882 | 0.884 | 0.886 | 0.887 | 0.889 | 0.891 |
| −4.682 | −4.728 | −4.776 | −4.825 | −4.875 | −4.928 | −4.981 | −5.036 | −5.093 | −5.150 | −5.209 | −5.268 | −5.329 | −5.390 | −5.452 | −5.514 | −5.578 |
| 2.389 | 2.353 | 2.314 | 2.275 | 2.233 | 2.189 | 2.144 | 2.098 | 2.050 | 2.000 | 1.949 | 1.897 | 1.844 | 1.790 | 1.735 | 1.679 | 1.622 |
| −5.629 | −5.687 | −5.748 | −5.811 | −5.877 | −5.945 | −6.016 | −6.088 | −6.164 | −6.240 | −6.320 | −6.401 | −6.484 | −6.568 | −6.655 | −6.742 | −6.832 |
| 2.584 | 2.543 | 2.500 | 2.456 | 2.411 | 2.364 | 2.316 | 2.266 | 2.215 | 2.163 | 2.109 | 2.054 | 1.998 | 1.941 | 1.883 | 1.824 | 1.765 |

| | | | | | | | | | | | | | | | | |
|---|---|---|---|---|---|---|---|---|---|---|---|---|---|---|---|---|
| 0.892 | 0.894 | 0.896 | 0.897 | 0.899 | 0.901 | 0.902 | 0.904 | 0.906 | 0.907 | 0.909 | 0.911 | 0.912 | 0.914 | 0.916 | 0.917 | 0.919 |
| | | | | | | | | | | | | | | | | |
| −5.642 | −5.707 | −5.772 | −5.838 | −5.905 | −5.971 | −6.038 | −6.105 | −6.172 | −6.238 | −6.304 | −6.368 | −6.433 | −6.496 | −6.558 | −6.619 | −6.680 |
| 1.566 | 1.508 | 1.450 | 1.393 | 1.334 | 1.276 | 1.218 | 1.159 | 1.100 | 1.041 | 0.982 | 0.923 | 0.863 | 0.804 | 0.744 | 0.685 | 0.625 |
| | | | | | | | | | | | | | | | | |
| −6.923 | −7.015 | −7.109 | −7.204 | −7.300 | −7.396 | −7.494 | −7.591 | −7.689 | −7.787 | −7.884 | −7.981 | −8.078 | −8.173 | −8.269 | −8.362 | −8.456 |
| 1.704 | 1.643 | 1.582 | 1.519 | 1.456 | 1.393 | 1.329 | 1.265 | 1.200 | 1.136 | 1.070 | 1.005 | 0.940 | 0.875 | 0.809 | 0.744 | 0.678 |

| | | | | | | | | | | | | | | | | |
|---|---|---|---|---|---|---|---|---|---|---|---|---|---|---|---|---|
| 0.921 | 0.922 | 0.924 | 0.926 | 0.927 | 0.929 | 0.931 | 0.932 | 0.934 | 0.936 | 0.937 | 0.939 | 0.941 | 0.942 | 0.944 | 0.946 | 0.947 |
| | | | | | | | | | | | | | | | | |
| −6.738 | −6.795 | −6.850 | −6.904 | −6.955 | −7.005 | −7.053 | −7.099 | −7.142 | −7.185 | −7.225 | −7.264 | −7.302 | −7.337 | −7.372 | −7.404 | −7.435 |
| 0.565 | 0.505 | 0.445 | 0.384 | 0.324 | 0.264 | 0.205 | 0.145 | 0.086 | 0.027 | −0.031 | −0.089 | −0.146 | −0.203 | −0.259 | −0.314 | −0.369 |
| | | | | | | | | | | | | | | | | |
| −8.547 | −8.638 | −8.726 | −8.814 | −8.899 | −8.982 | −9.064 | −9.143 | −9.220 | −9.295 | −9.368 | −9.438 | −9.507 | −9.574 | −9.638 | −9.699 | −9.758 |
| 0.613 | 0.547 | 0.482 | 0.416 | 0.351 | 0.286 | 0.222 | 0.158 | 0.094 | 0.031 | −0.031 | −0.092 | −0.152 | −0.211 | −0.269 | −0.325 | −0.380 |

| | | | | | | | | | | | | | | | | |
|---|---|---|---|---|---|---|---|---|---|---|---|---|---|---|---|---|
| 0.949 | 0.951 | 0.952 | 0.954 | 0.956 | 0.957 | 0.959 | 0.961 | 0.962 | 0.964 | 0.966 | 0.967 | 0.969 | 0.971 | 0.972 | 0.974 | 0.976 |
| | | | | | | | | | | | | | | | | |
| −7.465 | −7.493 | −7.519 | −7.545 | −7.569 | −7.592 | −7.614 | −7.635 | −7.655 | −7.675 | −7.694 | −7.712 | −7.730 | −7.748 | −7.766 | −7.783 | −7.801 |
| −0.422 | −0.475 | −0.526 | −0.577 | −0.626 | −0.675 | −0.723 | −0.770 | −0.816 | −0.861 | −0.905 | −0.948 | −0.990 | −1.031 | −1.070 | −1.107 | −1.144 |
| | | | | | | | | | | | | | | | | |
| −9.815 | −9.869 | −9.921 | −9.971 | −10.018 | −10.063 | −10.106 | −10.147 | −10.186 | −10.224 | −10.260 | −10.295 | −10.329 | −10.362 | −10.393 | −10.424 | −10.454 |
| −0.433 | −0.484 | −0.533 | −0.581 | −0.626 | −0.670 | −0.711 | −0.751 | −0.788 | −0.823 | −0.856 | −0.888 | −0.917 | −0.944 | −0.970 | −0.993 | −1.015 |

| | | | | | | | | | | | | | | | | |
|---|---|---|---|---|---|---|---|---|---|---|---|---|---|---|---|---|
| 0.977 | 0.979 | 0.981 | 0.982 | 0.984 | 0.986 | 0.987 | 0.989 | 0.991 | 0.992 | 0.994 | 0.996 | 0.997 | 0.999 | 1.001 | 1.002 | 1.004 |
| −7.818 | −7.835 | −7.853 | −7.870 | −7.887 | −7.904 | −7.922 | −7.940 | −7.958 | −7.977 | −7.997 | −8.017 | −8.038 | −8.060 | −8.082 | −8.105 | −8.129 |
| −1.178 | −1.211 | −1.243 | −1.273 | −1.301 | −1.328 | −1.353 | −1.377 | −1.399 | −1.419 | −1.438 | −1.455 | −1.470 | −1.484 | −1.495 | −1.506 | −1.514 |
| −10.483 | −10.510 | −10.537 | −10.563 | −10.588 | −10.613 | −10.637 | −10.660 | −10.682 | −10.704 | −10.725 | −10.746 | −10.766 | −10.786 | −10.806 | −10.824 | −10.842 |
| −1.035 | −1.052 | −1.068 | −1.082 | −1.094 | −1.103 | −1.110 | −1.115 | −1.118 | −1.119 | −1.117 | −1.113 | −1.107 | −1.098 | −1.087 | −1.074 | −1.059 |

| | | | | | | | | | | | | | | | | |
|---|---|---|---|---|---|---|---|---|---|---|---|---|---|---|---|---|
| 1.006 | 1.007 | 1.009 | 1.011 | 1.012 | 1.014 | 1.016 | 1.017 | 1.019 | 1.021 | 1.022 | 1.024 | 1.026 | 1.027 | 1.029 | 1.031 | 1.032 |
| | | | | | | | | | | | | | | | | |
| −8.155 | −8.181 | −8.208 | −8.235 | −8.264 | −8.293 | −8.322 | −8.352 | −8.382 | −8.413 | −8.444 | −8.475 | −8.507 | −8.539 | −8.571 | −8.604 | −8.637 |
| −1.520 | −1.525 | −1.528 | −1.529 | −1.528 | −1.525 | −1.520 | −1.512 | −1.503 | −1.492 | −1.479 | −1.464 | −1.448 | −1.429 | −1.408 | −1.386 | −1.361 |
| | | | | | | | | | | | | | | | | |
| −10.859 | −10.876 | −10.892 | −10.908 | −10.923 | −10.938 | −10.954 | −10.969 | −10.984 | −11.000 | −11.015 | −11.031 | −11.046 | −11.062 | −11.078 | −11.095 | −11.111 |
| −1.041 | −1.022 | −1.001 | −0.977 | −0.952 | −0.924 | −0.895 | −0.863 | −0.830 | −0.795 | −0.758 | −0.719 | −0.678 | −0.636 | −0.592 | −0.546 | −0.500 |

| | | | | | | | | | | | | | | | | |
|---|---|---|---|---|---|---|---|---|---|---|---|---|---|---|---|---|
| 1.034 | 1.036 | 1.037 | 1.039 | 1.041 | 1.042 | 1.044 | 1.046 | 1.047 | 1.049 | 1.051 | 1.052 | 1.054 | 1.056 | 1.057 | 1.059 | 1.061 |
| −8.670 | −8.704 | −8.739 | −8.774 | −8.810 | −8.847 | −8.885 | −8.925 | −8.965 | −9.007 | −9.050 | −9.094 | −9.138 | −9.184 | −9.230 | −9.276 | −9.323 |
| −1.335 | −1.306 | −1.276 | −1.243 | −1.209 | −1.174 | −1.136 | −1.098 | −1.057 | −1.016 | −0.972 | −0.928 | −0.882 | −0.836 | −0.788 | −0.739 | −0.689 |
| −11.128 | −11.144 | −11.161 | −11.177 | −11.194 | −11.211 | −11.228 | −11.245 | −11.262 | −11.279 | −11.297 | −11.316 | −11.335 | −11.354 | −11.374 | −11.394 | −11.414 |
| −0.452 | −0.403 | −0.354 | −0.304 | −0.254 | −0.203 | −0.153 | −0.102 | −0.050 | 0.001 | 0.054 | 0.106 | 0.159 | 0.212 | 0.265 | 0.318 | 0.371 |

| | | | | | | | | | | | | | | | | |
|---|---|---|---|---|---|---|---|---|---|---|---|---|---|---|---|---|
| 1.062 | 1.064 | 1.066 | 1.067 | 1.069 | 1.071 | 1.072 | 1.074 | 1.076 | 1.077 | 1.079 | 1.081 | 1.082 | 1.084 | 1.086 | 1.087 | 1.089 |
| −9.371 | −9.418 | −9.466 | −9.514 | −9.563 | −9.611 | −9.659 | −9.707 | −9.755 | −9.802 | −9.850 | −9.897 | −9.943 | −9.988 | −10.032 | −10.075 | −10.117 |
| −0.638 | −0.586 | −0.533 | −0.478 | −0.423 | −0.367 | −0.311 | −0.253 | −0.195 | −0.137 | −0.078 | −0.020 | 0.039 | 0.098 | 0.157 | 0.215 | 0.273 |
| −11.435 | −11.456 | −11.478 | −11.500 | −11.522 | −11.545 | −11.568 | −11.591 | −11.615 | −11.639 | −11.663 | −11.687 | −11.711 | −11.735 | −11.758 | −11.781 | −11.803 |
| 0.423 | 0.475 | 0.526 | 0.577 | 0.626 | 0.675 | 0.723 | 0.770 | 0.816 | 0.862 | 0.906 | 0.949 | 0.991 | 1.033 | 1.073 | 1.111 | 1.149 |

| | | | | | | | | | | | | | | | | |
|---|---|---|---|---|---|---|---|---|---|---|---|---|---|---|---|---|
| 1.091 | 1.092 | 1.094 | 1.096 | 1.097 | 1.099 | 1.101 | 1.102 | 1.104 | 1.106 | 1.107 | 1.109 | 1.111 | 1.112 | 1.114 | 1.116 | 1.117 |
| −10.157 | −10.197 | −10.235 | −10.271 | −10.306 | −10.339 | −10.371 | −10.400 | −10.428 | −10.453 | −10.476 | −10.497 | −10.516 | −10.532 | −10.546 | −10.557 | −10.567 |
| 0.331 | 0.388 | 0.445 | 0.502 | 0.559 | 0.617 | 0.674 | 0.731 | 0.788 | 0.845 | 0.903 | 0.960 | 1.017 | 1.074 | 1.131 | 1.188 | 1.244 |
| −11.825 | −11.846 | −11.866 | −11.885 | −11.902 | −11.918 | −11.932 | −11.944 | −11.954 | −11.962 | −11.968 | −11.972 | −11.974 | −11.974 | −11.972 | −11.968 | −11.962 |
| 1.186 | 1.221 | 1.255 | 1.288 | 1.320 | 1.351 | 1.381 | 1.410 | 1.439 | 1.466 | 1.493 | 1.520 | 1.545 | 1.571 | 1.596 | 1.620 | 1.644 |

| | | | | | | | | | | | | | | | | |
|---|---|---|---|---|---|---|---|---|---|---|---|---|---|---|---|---|
| 1.119 | 1.121 | 1.122 | 1.124 | 1.126 | 1.127 | 1.129 | 1.131 | 1.132 | 1.134 | 1.136 | 1.137 | 1.139 | 1.140 | 1.142 | 1.144 | 1.145 |
| −10.573 | −10.578 | −10.579 | −10.579 | −10.575 | −10.569 | −10.560 | −10.548 | −10.533 | −10.514 | −10.493 | −10.469 | −10.442 | −10.413 | −10.381 | −10.347 | −10.310 |
| 1.301 | 1.357 | 1.413 | 1.469 | 1.525 | 1.581 | 1.637 | 1.692 | 1.747 | 1.802 | 1.857 | 1.911 | 1.965 | 2.019 | 2.073 | 2.127 | 2.180 |
| −11.953 | −11.942 | −11.929 | −11.914 | −11.898 | −11.879 | −11.858 | −11.836 | −11.811 | −11.785 | −11.757 | −11.727 | −11.695 | −11.662 | −11.627 | −11.591 | −11.553 |
| 1.668 | 1.692 | 1.715 | 1.738 | 1.762 | 1.785 | 1.808 | 1.831 | 1.855 | 1.878 | 1.901 | 1.925 | 1.948 | 1.972 | 1.997 | 2.022 | 2.047 |

| | | | | | | | | | | | | | | | | |
|---|---|---|---|---|---|---|---|---|---|---|---|---|---|---|---|---|
| 1.147 | 1.149 | 1.150 | 1.152 | 1.154 | 1.155 | 1.157 | 1.159 | 1.160 | 1.162 | 1.164 | 1.165 | 1.167 | 1.169 | 1.170 | 1.172 | 1.174 |
| | | | | | | | | | | | | | | | | |
| −10.271 | −10.229 | −10.184 | −10.137 | −10.088 | −10.036 | −9.982 | −9.926 | −9.868 | −9.808 | −9.747 | −9.685 | −9.621 | −9.558 | −9.493 | −9.429 | −9.365 |
| 2.234 | 2.287 | 2.340 | 2.393 | 2.446 | 2.499 | 2.552 | 2.605 | 2.657 | 2.710 | 2.762 | 2.814 | 2.865 | 2.916 | 2.967 | 3.017 | 3.067 |
| | | | | | | | | | | | | | | | | |
| −11.514 | −11.473 | −11.431 | −11.388 | −11.342 | −11.296 | −11.248 | −11.199 | −11.149 | −11.098 | −11.046 | −10.994 | −10.942 | −10.889 | −10.836 | −10.783 | −10.730 |
| 2.073 | 2.100 | 2.128 | 2.157 | 2.187 | 2.219 | 2.251 | 2.285 | 2.320 | 2.356 | 2.394 | 2.432 | 2.472 | 2.512 | 2.554 | 2.597 | 2.640 |

| | | | | | | | | | | | | | | | | |
|---|---|---|---|---|---|---|---|---|---|---|---|---|---|---|---|---|
| 1.175 | 1.177 | 1.179 | 1.180 | 1.182 | 1.184 | 1.185 | 1.187 | 1.189 | 1.190 | 1.192 | 1.194 | 1.195 | 1.197 | 1.199 | 1.200 | 1.202 |
| −9.300 | −9.236 | −9.172 | −9.108 | −9.045 | −8.981 | −8.919 | −8.856 | −8.795 | −8.734 | −8.675 | −8.616 | −8.558 | −8.501 | −8.446 | −8.392 | −8.338 |
| 3.117 | 3.166 | 3.214 | 3.262 | 3.310 | 3.357 | 3.404 | 3.450 | 3.495 | 3.540 | 3.585 | 3.629 | 3.673 | 3.716 | 3.760 | 3.803 | 3.846 |
| −10.677 | −10.624 | −10.572 | −10.520 | −10.468 | −10.417 | −10.367 | −10.318 | −10.270 | −10.223 | −10.177 | −10.132 | −10.088 | −10.046 | −10.005 | −9.966 | −9.929 |
| 2.685 | 2.731 | 2.777 | 2.824 | 2.872 | 2.921 | 2.970 | 3.019 | 3.069 | 3.119 | 3.170 | 3.221 | 3.272 | 3.323 | 3.375 | 3.426 | 3.477 |

| | | | | | | | | | | | | | | | | |
|---|---|---|---|---|---|---|---|---|---|---|---|---|---|---|---|---|
| 1.204 | 1.205 | 1.207 | 1.209 | 1.210 | 1.212 | 1.214 | 1.215 | 1.217 | 1.219 | 1.220 | 1.222 | 1.224 | 1.225 | 1.227 | 1.229 | 1.230 |
| −8.287 | −8.237 | −8.188 | −8.141 | −8.097 | −8.054 | −8.014 | −7.976 | −7.940 | −7.907 | −7.877 | −7.849 | −7.824 | −7.801 | −7.782 | −7.766 | −7.752 |
| 3.889 | 3.932 | 3.975 | 4.019 | 4.063 | 4.107 | 4.151 | 4.196 | 4.241 | 4.286 | 4.331 | 4.376 | 4.421 | 4.467 | 4.512 | 4.558 | 4.604 |
| −9.893 | −9.859 | −9.827 | −9.797 | −9.769 | −9.743 | −9.718 | −9.696 | −9.675 | −9.656 | −9.639 | −9.624 | −9.610 | −9.598 | −9.588 | −9.579 | −9.573 |
| 3.529 | 3.579 | 3.630 | 3.680 | 3.729 | 3.778 | 3.826 | 3.874 | 3.922 | 3.969 | 4.015 | 4.061 | 4.106 | 4.151 | 4.196 | 4.239 | 4.282 |

| | | | | | | | | | | | | | | | | |
|---|---|---|---|---|---|---|---|---|---|---|---|---|---|---|---|---|
| 1.232 | 1.234 | 1.235 | 1.237 | 1.239 | 1.240 | 1.242 | 1.244 | 1.245 | 1.247 | 1.249 | 1.250 | 1.252 | 1.254 | 1.255 | 1.257 | 1.259 |
| −7.741 | −7.733 | −7.727 | −7.725 | −7.724 | −7.727 | −7.732 | −7.739 | −7.749 | −7.762 | −7.776 | −7.793 | −7.812 | −7.832 | −7.855 | −7.878 | −7.903 |
| 4.650 | 4.696 | 4.743 | 4.789 | 4.835 | 4.881 | 4.927 | 4.973 | 5.019 | 5.064 | 5.110 | 5.155 | 5.199 | 5.243 | 5.286 | 5.329 | 5.370 |
| −9.567 | −9.565 | −9.563 | −9.564 | −9.567 | −9.571 | −9.577 | −9.584 | −9.593 | −9.603 | −9.613 | −9.625 | −9.637 | −9.650 | −9.664 | −9.680 | −9.697 |
| 4.325 | 4.366 | 4.406 | 4.445 | 4.484 | 4.521 | 4.557 | 4.592 | 4.626 | 4.659 | 4.691 | 4.721 | 4.751 | 4.780 | 4.807 | 4.834 | 4.859 |

| | | | | | | | | | | | | | | | | |
|---|---|---|---|---|---|---|---|---|---|---|---|---|---|---|---|---|
| 1.260 | 1.262 | 1.264 | 1.265 | 1.267 | 1.269 | 1.270 | 1.272 | 1.274 | 1.275 | 1.277 | 1.279 | 1.280 | 1.282 | 1.284 | 1.285 | 1.287 |
| −7.930 | −7.957 | −7.986 | −8.016 | −8.046 | −8.076 | −8.107 | −8.137 | −8.167 | −8.197 | −8.225 | −8.253 | −8.280 | −8.306 | −8.330 | −8.354 | −8.375 |
| 5.412 | 5.452 | 5.492 | 5.530 | 5.569 | 5.606 | 5.643 | 5.679 | 5.715 | 5.750 | 5.784 | 5.818 | 5.852 | 5.885 | 5.918 | 5.951 | 5.983 |
| −9.714 | −9.734 | −9.754 | −9.775 | −9.797 | −9.820 | −9.843 | −9.867 | −9.891 | −9.916 | −9.940 | −9.964 | −9.988 | −10.011 | −10.033 | −10.055 | −10.075 |
| 4.883 | 4.905 | 4.927 | 4.948 | 4.967 | 4.985 | 5.002 | 5.019 | 5.034 | 5.048 | 5.061 | 5.072 | 5.083 | 5.093 | 5.102 | 5.110 | 5.117 |

| | | | | | | | | | | | | | | | | |
|---|---|---|---|---|---|---|---|---|---|---|---|---|---|---|---|---|
| 1.289 | 1.290 | 1.292 | 1.294 | 1.295 | 1.297 | 1.299 | 1.300 | 1.302 | 1.304 | 1.305 | 1.307 | 1.309 | 1.310 | 1.312 | 1.314 | 1.315 |
| −8.396 | −8.415 | −8.433 | −8.450 | −8.467 | −8.481 | −8.495 | −8.507 | −8.517 | −8.526 | −8.533 | −8.539 | −8.544 | −8.547 | −8.548 | −8.548 | −8.547 |
| 6.015 | 6.047 | 6.079 | 6.111 | 6.142 | 6.174 | 6.205 | 6.236 | 6.267 | 6.298 | 6.329 | 6.360 | 6.391 | 6.422 | 6.453 | 6.485 | 6.516 |
| −10.094 | −10.111 | −10.128 | −10.142 | −10.156 | −10.168 | −10.178 | −10.186 | −10.193 | −10.197 | −10.199 | −10.199 | −10.197 | −10.193 | −10.187 | −10.179 | −10.169 |
| 5.124 | 5.130 | 5.135 | 5.140 | 5.144 | 5.148 | 5.151 | 5.154 | 5.157 | 5.161 | 5.164 | 5.168 | 5.172 | 5.177 | 5.182 | 5.188 | 5.194 |

| | | | | | | | | | | | | | | | | |
|---|---|---|---|---|---|---|---|---|---|---|---|---|---|---|---|---|
| 1.317 | 1.319 | 1.320 | 1.322 | 1.324 | 1.325 | 1.327 | 1.329 | 1.330 | 1.332 | 1.334 | 1.335 | 1.337 | 1.339 | 1.340 | 1.342 | 1.344 |
| −8.544 | −8.540 | −8.535 | −8.529 | −8.522 | −8.513 | −8.503 | −8.491 | −8.478 | −8.464 | −8.449 | −8.432 | −8.414 | −8.394 | −8.374 | −8.352 | −8.330 |
| 6.548 | 6.580 | 6.612 | 6.644 | 6.677 | 6.709 | 6.741 | 6.774 | 6.806 | 6.838 | 6.869 | 6.901 | 6.931 | 6.961 | 6.991 | 7.020 | 7.049 |
| −10.157 | −10.143 | −10.127 | −10.110 | −10.090 | −10.069 | −10.046 | −10.021 | −9.995 | −9.966 | −9.935 | −9.903 | −9.869 | −9.833 | −9.795 | −9.756 | −9.715 |
| 5.200 | 5.207 | 5.214 | 5.222 | 5.230 | 5.239 | 5.247 | 5.256 | 5.265 | 5.275 | 5.285 | 5.295 | 5.305 | 5.316 | 5.326 | 5.338 | 5.349 |

| | | | | | | | | | | | | | | | | |
|---|---|---|---|---|---|---|---|---|---|---|---|---|---|---|---|---|
| 1.345 | 1.347 | 1.349 | 1.350 | 1.352 | 1.354 | 1.355 | 1.357 | 1.359 | 1.360 | 1.362 | 1.364 | 1.365 | 1.367 | 1.369 | 1.370 | 1.372 |
| | | | | | | | | | | | | | | | | |
| −8.305 | −8.280 | −8.253 | −8.225 | −8.195 | −8.164 | −8.131 | −8.096 | −8.060 | −8.023 | −7.984 | −7.943 | −7.902 | −7.860 | −7.817 | −7.774 | −7.730 |
| 7.077 | 7.105 | 7.133 | 7.160 | 7.188 | 7.215 | 7.242 | 7.270 | 7.297 | 7.324 | 7.352 | 7.379 | 7.406 | 7.433 | 7.460 | 7.487 | 7.514 |
| | | | | | | | | | | | | | | | | |
| −9.673 | −9.630 | −9.585 | −9.539 | −9.491 | −9.442 | −9.392 | −9.341 | −9.289 | −9.235 | −9.181 | −9.125 | −9.069 | −9.011 | −8.952 | −8.893 | −8.832 |
| 5.362 | 5.374 | 5.388 | 5.401 | 5.416 | 5.431 | 5.446 | 5.463 | 5.480 | 5.499 | 5.518 | 5.539 | 5.560 | 5.583 | 5.607 | 5.632 | 5.659 |

| | | | | | | | | | | | | | | | | |
|---|---|---|---|---|---|---|---|---|---|---|---|---|---|---|---|---|
| 1.374 | 1.375 | 1.377 | 1.379 | 1.380 | 1.382 | 1.384 | 1.385 | 1.387 | 1.389 | 1.390 | 1.392 | 1.394 | 1.395 | 1.397 | 1.399 | 1.400 |
| −7.686 | −7.641 | −7.597 | −7.553 | −7.509 | −7.466 | −7.423 | −7.381 | −7.340 | −7.300 | −7.261 | −7.224 | −7.187 | −7.153 | −7.120 | −7.089 | −7.059 |
| 7.541 | 7.568 | 7.595 | 7.622 | 7.648 | 7.675 | 7.701 | 7.728 | 7.754 | 7.780 | 7.806 | 7.831 | 7.856 | 7.881 | 7.906 | 7.930 | 7.953 |
| −8.771 | −8.708 | −8.645 | −8.581 | −8.517 | −8.452 | −8.387 | −8.322 | −8.256 | −8.190 | −8.124 | −8.058 | −7.992 | −7.927 | −7.862 | −7.798 | −7.735 |
| 5.686 | 5.714 | 5.743 | 5.774 | 5.805 | 5.837 | 5.869 | 5.902 | 5.936 | 5.970 | 6.004 | 6.039 | 6.074 | 6.110 | 6.146 | 6.182 | 6.218 |

| | | | | | | | | | | | | | | | | |
|---|---|---|---|---|---|---|---|---|---|---|---|---|---|---|---|---|
| 1.402 | 1.404 | 1.405 | 1.407 | 1.409 | 1.410 | 1.412 | 1.414 | 1.415 | 1.417 | 1.419 | 1.420 | 1.422 | 1.424 | 1.425 | 1.427 | 1.429 |
| −7.031 | −7.005 | −6.980 | −6.957 | −6.936 | −6.916 | −6.898 | −6.882 | −6.867 | −6.855 | −6.844 | −6.834 | −6.827 | −6.821 | −6.817 | −6.815 | −6.815 |
| 7.976 | 7.997 | 8.018 | 8.038 | 8.057 | 8.075 | 8.092 | 8.108 | 8.122 | 8.135 | 8.147 | 8.158 | 8.167 | 8.174 | 8.181 | 8.185 | 8.188 |
| −7.673 | −7.612 | −7.552 | −7.494 | −7.438 | −7.383 | −7.330 | −7.278 | −7.229 | −7.181 | −7.136 | −7.093 | −7.052 | −7.013 | −6.977 | −6.944 | −6.913 |
| 6.254 | 6.290 | 6.326 | 6.362 | 6.398 | 6.433 | 6.468 | 6.503 | 6.536 | 6.569 | 6.601 | 6.631 | 6.660 | 6.688 | 6.714 | 6.738 | 6.759 |

| | | | | | | | | | | | | | | | | |
|---|---|---|---|---|---|---|---|---|---|---|---|---|---|---|---|---|
| 1.430 | 1.432 | 1.434 | 1.435 | 1.437 | 1.439 | 1.440 | 1.442 | 1.444 | 1.445 | 1.447 | 1.449 | 1.450 | 1.452 | 1.454 | 1.455 | 1.457 |
| −6.818 | −6.822 | −6.828 | −6.836 | −6.846 | −6.858 | −6.873 | −6.889 | −6.908 | −6.928 | −6.951 | −6.977 | −7.004 | −7.034 | −7.066 | −7.099 | −7.135 |
| 8.189 | 8.188 | 8.185 | 8.180 | 8.174 | 8.165 | 8.155 | 8.142 | 8.127 | 8.110 | 8.090 | 8.069 | 8.045 | 8.019 | 7.990 | 7.959 | 7.926 |
| −6.886 | −6.861 | −6.840 | −6.821 | −6.807 | −6.795 | −6.787 | −6.782 | −6.781 | −6.784 | −6.790 | −6.799 | −6.812 | −6.828 | −6.847 | −6.870 | −6.896 |
| 6.779 | 6.797 | 6.812 | 6.824 | 6.835 | 6.842 | 6.848 | 6.850 | 6.850 | 6.847 | 6.840 | 6.831 | 6.819 | 6.804 | 6.785 | 6.764 | 6.739 |

| | | | | | | | | | | | | | | | | |
|---|---|---|---|---|---|---|---|---|---|---|---|---|---|---|---|---|
| 1.459 | 1.460 | 1.462 | 1.463 | 1.465 | 1.467 | 1.468 | 1.470 | 1.472 | 1.473 | 1.475 | 1.477 | 1.478 | 1.480 | 1.482 | 1.483 | 1.485 |
| −7.172 | −7.210 | −7.251 | −7.292 | −7.335 | −7.379 | −7.423 | −7.469 | −7.516 | −7.563 | −7.611 | −7.660 | −7.709 | −7.759 | −7.810 | −7.860 | −7.911 |
| 7.890 | 7.852 | 7.811 | 7.767 | 7.722 | 7.673 | 7.622 | 7.569 | 7.513 | 7.454 | 7.393 | 7.329 | 7.262 | 7.193 | 7.122 | 7.048 | 6.972 |
| −6.926 | −6.959 | −6.996 | −7.036 | −7.079 | −7.125 | −7.174 | −7.226 | −7.282 | −7.340 | −7.402 | −7.466 | −7.533 | −7.603 | −7.676 | −7.751 | −7.829 |
| 6.711 | 6.679 | 6.644 | 6.606 | 6.565 | 6.521 | 6.473 | 6.422 | 6.368 | 6.311 | 6.252 | 6.189 | 6.123 | 6.055 | 5.984 | 5.911 | 5.836 |

| | | | | | | | | | | | | | | | | |
|---|---|---|---|---|---|---|---|---|---|---|---|---|---|---|---|---|
| 1.487 | 1.488 | 1.490 | 1.492 | 1.493 | 1.495 | 1.497 | 1.498 | 1.500 | 1.502 | 1.503 | 1.505 | 1.507 | 1.508 | 1.510 | 1.512 | 1.513 |
| | | | | | | | | | | | | | | | | |
| −7.962 | −8.012 | −8.063 | −8.114 | −8.164 | −8.213 | −8.262 | −8.311 | −8.358 | −8.404 | −8.450 | −8.495 | −8.539 | −8.582 | −8.625 | −8.667 | −8.708 |
| 6.894 | 6.813 | 6.731 | 6.647 | 6.562 | 6.474 | 6.385 | 6.294 | 6.202 | 6.107 | 6.011 | 5.913 | 5.814 | 5.714 | 5.612 | 5.509 | 5.406 |
| | | | | | | | | | | | | | | | | |
| −7.908 | −7.990 | −8.073 | −8.159 | −8.246 | −8.336 | −8.426 | −8.519 | −8.613 | −8.707 | −8.803 | −8.900 | −8.996 | −9.093 | −9.190 | −9.286 | −9.382 |
| 5.759 | 5.680 | 5.599 | 5.517 | 5.432 | 5.347 | 5.259 | 5.171 | 5.081 | 4.989 | 4.897 | 4.803 | 4.709 | 4.614 | 4.519 | 4.423 | 4.327 |

| | | | | | | | | | | | | | | | | |
|---|---|---|---|---|---|---|---|---|---|---|---|---|---|---|---|---|
| 1.515 | 1.517 | 1.518 | 1.520 | 1.522 | 1.523 | 1.525 | 1.527 | 1.528 | 1.530 | 1.532 | 1.533 | 1.535 | 1.537 | 1.538 | 1.540 | 1.542 |
| −8.748 | −8.787 | −8.826 | −8.863 | −8.899 | −8.935 | −8.968 | −9.001 | −9.033 | −9.063 | −9.092 | −9.119 | −9.145 | −9.169 | −9.192 | −9.214 | −9.234 |
| 5.301 | 5.195 | 5.089 | 4.981 | 4.873 | 4.765 | 4.656 | 4.546 | 4.436 | 4.326 | 4.217 | 4.107 | 3.997 | 3.888 | 3.779 | 3.671 | 3.563 |
| −9.477 | −9.572 | −9.666 | −9.759 | −9.851 | −9.942 | −10.032 | −10.121 | −10.208 | −10.293 | −10.377 | −10.458 | −10.538 | −10.616 | −10.691 | −10.765 | −10.836 |
| 4.232 | 4.136 | 4.041 | 3.946 | 3.852 | 3.757 | 3.664 | 3.572 | 3.480 | 3.389 | 3.300 | 3.212 | 3.126 | 3.042 | 2.959 | 2.878 | 2.800 |

| | | | | | | | | | | | | | | | | |
|---|---|---|---|---|---|---|---|---|---|---|---|---|---|---|---|---|
| 1.543 | 1.545 | 1.547 | 1.548 | 1.550 | 1.552 | 1.553 | 1.555 | 1.557 | 1.558 | 1.560 | 1.562 | 1.563 | 1.565 | 1.567 | 1.568 | 1.570 |
| −9.253 | −9.272 | −9.289 | −9.305 | −9.320 | −9.335 | −9.348 | −9.361 | −9.372 | −9.383 | −9.393 | −9.402 | −9.410 | −9.418 | −9.426 | −9.432 | −9.438 |
| 3.457 | 3.352 | 3.247 | 3.144 | 3.042 | 2.941 | 2.842 | 2.745 | 2.649 | 2.555 | 2.463 | 2.373 | 2.286 | 2.201 | 2.120 | 2.041 | 1.965 |
| −10.905 | −10.971 | −11.035 | −11.096 | −11.155 | −11.212 | −11.265 | −11.316 | −11.364 | −11.409 | −11.451 | −11.491 | −11.528 | −11.561 | −11.592 | −11.619 | −11.642 |
| 2.724 | 2.650 | 2.579 | 2.510 | 2.445 | 2.382 | 2.323 | 2.267 | 2.214 | 2.164 | 2.118 | 2.075 | 2.035 | 1.999 | 1.965 | 1.936 | 1.910 |

| | | | | | | | | | | | | | | | | |
|---|---|---|---|---|---|---|---|---|---|---|---|---|---|---|---|---|
| 1.572 | 1.573 | 1.575 | 1.577 | 1.578 | 1.580 | 1.582 | 1.583 | 1.585 | 1.587 | 1.588 | 1.590 | 1.592 | 1.593 | 1.595 | 1.597 | 1.598 |
| | | | | | | | | | | | | | | | | |
| −9.443 | −9.446 | −9.449 | −9.450 | −9.451 | −9.451 | −9.450 | −9.447 | −9.444 | −9.440 | −9.435 | −9.428 | −9.421 | −9.413 | −9.403 | −9.392 | −9.379 |
| 1.892 | 1.823 | 1.757 | 1.695 | 1.637 | 1.581 | 1.530 | 1.482 | 1.437 | 1.397 | 1.359 | 1.325 | 1.294 | 1.267 | 1.243 | 1.221 | 1.203 |
| | | | | | | | | | | | | | | | | |
| −11.662 | −11.679 | −11.692 | −11.701 | −11.707 | −11.710 | −11.709 | −11.705 | −11.698 | −11.689 | −11.676 | −11.661 | −11.643 | −11.622 | −11.599 | −11.574 | −11.546 |
| 1.887 | 1.868 | 1.852 | 1.839 | 1.829 | 1.821 | 1.817 | 1.815 | 1.816 | 1.819 | 1.825 | 1.833 | 1.844 | 1.857 | 1.872 | 1.890 | 1.909 |

| | | | | | | | | | | | | | | | | |
|---|---|---|---|---|---|---|---|---|---|---|---|---|---|---|---|---|
| 1.600 | 1.602 | 1.603 | 1.605 | 1.607 | 1.608 | 1.610 | 1.612 | 1.613 | 1.615 | 1.617 | 1.618 | 1.620 | 1.622 | 1.623 | 1.625 | 1.627 |
| −9.366 | −9.351 | −9.335 | −9.318 | −9.299 | −9.280 | −9.259 | −9.237 | −9.215 | −9.192 | −9.168 | −9.144 | −9.119 | −9.094 | −9.069 | −9.044 | −9.019 |
| 1.188 | 1.176 | 1.166 | 1.160 | 1.156 | 1.154 | 1.155 | 1.159 | 1.165 | 1.173 | 1.184 | 1.197 | 1.211 | 1.228 | 1.246 | 1.266 | 1.288 |
| −11.515 | −11.483 | −11.448 | −11.411 | −11.372 | −11.331 | −11.289 | −11.245 | −11.199 | −11.152 | −11.104 | −11.055 | −11.006 | −10.956 | −10.905 | −10.855 | −10.805 |
| 1.931 | 1.955 | 1.980 | 2.007 | 2.035 | 2.065 | 2.096 | 2.128 | 2.161 | 2.195 | 2.230 | 2.265 | 2.301 | 2.338 | 2.375 | 2.412 | 2.449 |

| | | | | | | | | | | | | | | | | |
|---|---|---|---|---|---|---|---|---|---|---|---|---|---|---|---|---|
| 1.628 | 1.630 | 1.632 | 1.633 | 1.635 | 1.637 | 1.638 | 1.640 | 1.642 | 1.643 | 1.645 | 1.647 | 1.648 | 1.650 | 1.652 | 1.653 | 1.655 |
| | | | | | | | | | | | | | | | | |
| −8.993 | −8.968 | −8.943 | −8.919 | −8.895 | −8.872 | −8.849 | −8.827 | −8.806 | −8.785 | −8.766 | −8.746 | −8.728 | −8.710 | −8.694 | −8.677 | −8.662 |
| 1.311 | 1.335 | 1.361 | 1.389 | 1.417 | 1.446 | 1.476 | 1.508 | 1.540 | 1.572 | 1.605 | 1.638 | 1.672 | 1.705 | 1.738 | 1.771 | 1.803 |
| | | | | | | | | | | | | | | | | |
| −10.756 | −10.707 | −10.659 | −10.612 | −10.565 | −10.520 | −10.476 | −10.434 | −10.393 | −10.353 | −10.316 | −10.279 | −10.245 | −10.212 | −10.180 | −10.150 | −10.122 |
| 2.486 | 2.524 | 2.561 | 2.598 | 2.634 | 2.670 | 2.706 | 2.740 | 2.774 | 2.806 | 2.838 | 2.868 | 2.898 | 2.925 | 2.952 | 2.977 | 3.000 |

| | | | | | | | | | | | | | | | | |
|---|---|---|---|---|---|---|---|---|---|---|---|---|---|---|---|---|
| 1.657 | 1.658 | 1.660 | 1.662 | 1.663 | 1.665 | 1.667 | 1.668 | 1.670 | 1.672 | 1.673 | 1.675 | 1.677 | 1.678 | 1.680 | 1.682 | 1.683 |
| −8.647 | −8.632 | −8.619 | −8.607 | −8.595 | −8.584 | −8.574 | −8.565 | −8.557 | −8.550 | −8.544 | −8.539 | −8.535 | −8.532 | −8.531 | −8.530 | −8.532 |
| 1.835 | 1.867 | 1.898 | 1.929 | 1.960 | 1.990 | 2.020 | 2.049 | 2.077 | 2.105 | 2.133 | 2.159 | 2.185 | 2.211 | 2.236 | 2.260 | 2.284 |
| −10.095 | −10.071 | −10.049 | −10.029 | −10.012 | −9.997 | −9.985 | −9.976 | −9.969 | −9.965 | −9.964 | −9.965 | −9.969 | −9.975 | −9.984 | −9.996 | −10.010 |
| 3.021 | 3.041 | 3.059 | 3.075 | 3.090 | 3.102 | 3.113 | 3.123 | 3.130 | 3.136 | 3.140 | 3.143 | 3.144 | 3.144 | 3.142 | 3.139 | 3.134 |

| | | | | | | | | | | | | | | | | |
|---|---|---|---|---|---|---|---|---|---|---|---|---|---|---|---|---|
| 1.685 | 1.687 | 1.688 | 1.690 | 1.692 | 1.693 | 1.695 | 1.697 | 1.698 | 1.700 | 1.702 | 1.703 | 1.705 | 1.707 | 1.708 | 1.710 | 1.712 |
| −8.534 | −8.538 | −8.543 | −8.550 | −8.558 | −8.567 | −8.578 | −8.590 | −8.602 | −8.616 | −8.631 | −8.647 | −8.663 | −8.681 | −8.700 | −8.720 | −8.741 |
| 2.308 | 2.331 | 2.353 | 2.374 | 2.394 | 2.414 | 2.432 | 2.449 | 2.465 | 2.480 | 2.494 | 2.506 | 2.517 | 2.526 | 2.535 | 2.542 | 2.548 |
| −10.027 | −10.046 | −10.068 | −10.093 | −10.119 | −10.148 | −10.179 | −10.213 | −10.249 | −10.287 | −10.327 | −10.369 | −10.414 | −10.460 | −10.508 | −10.558 | −10.609 |
| 3.128 | 3.120 | 3.111 | 3.101 | 3.089 | 3.076 | 3.062 | 3.048 | 3.032 | 3.016 | 2.999 | 2.981 | 2.962 | 2.944 | 2.924 | 2.905 | 2.884 |

| | | | | | | | | | | | | | | | | |
|---|---|---|---|---|---|---|---|---|---|---|---|---|---|---|---|---|
| 1.713 | 1.715 | 1.717 | 1.718 | 1.720 | 1.722 | 1.723 | 1.725 | 1.727 | 1.728 | 1.730 | 1.732 | 1.733 | 1.735 | 1.737 | 1.738 | 1.740 |
| −8.764 | −8.787 | −8.813 | −8.839 | −8.867 | −8.896 | −8.926 | −8.957 | −8.989 | −9.022 | −9.056 | −9.091 | −9.127 | −9.164 | −9.202 | −9.240 | −9.280 |
| 2.553 | 2.558 | 2.561 | 2.564 | 2.566 | 2.567 | 2.568 | 2.569 | 2.569 | 2.570 | 2.570 | 2.570 | 2.570 | 2.570 | 2.571 | 2.572 | 2.573 |
| −10.661 | −10.714 | −10.768 | −10.823 | −10.878 | −10.933 | −10.988 | −11.043 | −11.098 | −11.153 | −11.207 | −11.261 | −11.315 | −11.368 | −11.421 | −11.473 | −11.524 |
| 2.864 | 2.844 | 2.823 | 2.803 | 2.782 | 2.762 | 2.742 | 2.723 | 2.703 | 2.684 | 2.666 | 2.648 | 2.630 | 2.613 | 2.597 | 2.581 | 2.567 |

| | | | | | | | | | | | | | | | | |
|---|---|---|---|---|---|---|---|---|---|---|---|---|---|---|---|---|
| 1.742 | 1.743 | 1.745 | 1.747 | 1.748 | 1.750 | 1.752 | 1.753 | 1.755 | 1.757 | 1.758 | 1.760 | 1.762 | 1.763 | 1.765 | 1.767 | 1.768 |
| −9.320 | −9.360 | −9.400 | −9.440 | −9.480 | −9.520 | −9.558 | −9.597 | −9.634 | −9.671 | −9.707 | −9.741 | −9.774 | −9.805 | −9.835 | −9.862 | −9.888 |
| 2.575 | 2.578 | 2.582 | 2.586 | 2.591 | 2.597 | 2.603 | 2.611 | 2.619 | 2.628 | 2.638 | 2.649 | 2.660 | 2.673 | 2.686 | 2.700 | 2.715 |
| −11.574 | −11.624 | −11.672 | −11.719 | −11.764 | −11.808 | −11.850 | −11.890 | −11.928 | −11.964 | −11.998 | −12.030 | −12.060 | −12.087 | −12.112 | −12.134 | −12.154 |
| 2.553 | 2.541 | 2.529 | 2.520 | 2.512 | 2.506 | 2.501 | 2.499 | 2.498 | 2.500 | 2.503 | 2.508 | 2.514 | 2.522 | 2.531 | 2.541 | 2.553 |

| | | | | | | | | | | | | | | | | |
|---|---|---|---|---|---|---|---|---|---|---|---|---|---|---|---|---|
| 1.770 | 1.772 | 1.773 | 1.775 | 1.777 | 1.778 | 1.780 | 1.782 | 1.783 | 1.785 | 1.787 | 1.788 | 1.790 | 1.791 | 1.793 | 1.795 | 1.796 |
| −9.912 | −9.934 | −9.954 | −9.973 | −9.989 | −10.003 | −10.016 | −10.027 | −10.036 | −10.044 | −10.051 | −10.056 | −10.060 | −10.063 | −10.065 | −10.066 | −10.066 |
| 2.731 | 2.748 | 2.765 | 2.783 | 2.802 | 2.822 | 2.842 | 2.863 | 2.885 | 2.908 | 2.932 | 2.957 | 2.983 | 3.010 | 3.038 | 3.066 | 3.096 |
| −12.171 | −12.186 | −12.199 | −12.209 | −12.216 | −12.222 | −12.225 | −12.227 | −12.227 | −12.226 | −12.223 | −12.219 | −12.213 | −12.207 | −12.200 | −12.193 | −12.184 |
| 2.565 | 2.579 | 2.593 | 2.609 | 2.625 | 2.642 | 2.659 | 2.676 | 2.695 | 2.713 | 2.732 | 2.751 | 2.771 | 2.791 | 2.811 | 2.832 | 2.853 |

| | | | | | | | | | | | | | | | | |
|---|---|---|---|---|---|---|---|---|---|---|---|---|---|---|---|---|
| 1.798 | 1.800 | 1.801 | 1.803 | 1.805 | 1.806 | 1.808 | 1.810 | 1.811 | 1.813 | 1.815 | 1.816 | 1.818 | 1.820 | 1.821 | 1.823 | 1.825 |
| −10.066 | −10.065 | −10.064 | −10.063 | −10.062 | −10.061 | −10.060 | −10.058 | −10.057 | −10.055 | −10.054 | −10.052 | −10.051 | −10.049 | −10.047 | −10.046 | −10.043 |
| 3.126 | 3.156 | 3.187 | 3.218 | 3.250 | 3.282 | 3.314 | 3.347 | 3.379 | 3.412 | 3.445 | 3.477 | 3.510 | 3.542 | 3.574 | 3.605 | 3.636 |
| −12.175 | −12.165 | −12.155 | −12.144 | −12.132 | −12.120 | −12.107 | −12.092 | −12.077 | −12.061 | −12.044 | −12.025 | −12.006 | −11.985 | −11.963 | −11.941 | −11.917 |
| 2.876 | 2.899 | 2.922 | 2.946 | 2.971 | 2.996 | 3.022 | 3.048 | 3.075 | 3.103 | 3.131 | 3.159 | 3.188 | 3.217 | 3.246 | 3.276 | 3.306 |

| | | | | | | | | | | | | | | | | |
|---|---|---|---|---|---|---|---|---|---|---|---|---|---|---|---|---|
| 1.826 | 1.828 | 1.830 | 1.831 | 1.833 | 1.835 | 1.836 | 1.838 | 1.840 | 1.841 | 1.843 | 1.845 | 1.846 | 1.848 | 1.850 | 1.851 | 1.853 |
| −10.041 | −10.038 | −10.035 | −10.031 | −10.026 | −10.020 | −10.013 | −10.005 | −9.996 | −9.985 | −9.973 | −9.960 | −9.945 | −9.928 | −9.910 | −9.890 | −9.869 |
| 3.667 | 3.698 | 3.728 | 3.757 | 3.786 | 3.815 | 3.843 | 3.870 | 3.897 | 3.923 | 3.948 | 3.972 | 3.996 | 4.018 | 4.040 | 4.061 | 4.080 |
| −11.892 | −11.867 | −11.841 | −11.814 | −11.787 | −11.758 | −11.730 | −11.700 | −11.670 | −11.640 | −11.609 | −11.578 | −11.546 | −11.514 | −11.481 | −11.448 | −11.413 |
| 3.336 | 3.366 | 3.397 | 3.427 | 3.457 | 3.487 | 3.517 | 3.547 | 3.576 | 3.604 | 3.632 | 3.660 | 3.686 | 3.712 | 3.737 | 3.760 | 3.783 |

| | | | | | | | | | | | | | | | | |
|---|---|---|---|---|---|---|---|---|---|---|---|---|---|---|---|---|
| 1.855 | 1.856 | 1.858 | 1.860 | 1.861 | 1.863 | 1.865 | 1.866 | 1.868 | 1.870 | 1.871 | 1.873 | 1.875 | 1.876 | 1.878 | 1.880 | 1.881 |
| −9.846 | −9.822 | −9.796 | −9.769 | −9.741 | −9.711 | −9.679 | −9.646 | −9.611 | −9.574 | −9.536 | −9.496 | −9.454 | −9.411 | −9.366 | −9.319 | −9.272 |
| 4.098 | 4.116 | 4.131 | 4.146 | 4.159 | 4.171 | 4.182 | 4.192 | 4.200 | 4.206 | 4.211 | 4.215 | 4.217 | 4.218 | 4.217 | 4.215 | 4.211 |
| −11.378 | −11.342 | −11.305 | −11.267 | −11.227 | −11.186 | −11.143 | −11.099 | −11.054 | −11.007 | −10.958 | −10.908 | −10.856 | −10.803 | −10.749 | −10.693 | −10.635 |
| 3.804 | 3.824 | 3.842 | 3.858 | 3.872 | 3.885 | 3.896 | 3.905 | 3.913 | 3.918 | 3.923 | 3.925 | 3.926 | 3.926 | 3.925 | 3.922 | 3.918 |

| | | | | | | | | | | | | | | | | |
|---|---|---|---|---|---|---|---|---|---|---|---|---|---|---|---|---|
| 1.883 | 1.885 | 1.886 | 1.888 | 1.890 | 1.891 | 1.893 | 1.895 | 1.896 | 1.898 | 1.900 | 1.901 | 1.903 | 1.905 | 1.906 | 1.908 | 1.910 |
| −9.222 | −9.172 | −9.121 | −9.068 | −9.015 | −8.960 | −8.905 | −8.849 | −8.792 | −8.735 | −8.676 | −8.615 | −8.554 | −8.492 | −8.428 | −8.363 | −8.297 |
| 4.206 | 4.200 | 4.192 | 4.183 | 4.173 | 4.162 | 4.150 | 4.138 | 4.125 | 4.111 | 4.097 | 4.082 | 4.067 | 4.051 | 4.035 | 4.019 | 4.003 |
| −10.576 | −10.517 | −10.456 | −10.393 | −10.330 | −10.266 | −10.201 | −10.135 | −10.068 | −10.001 | −9.932 | −9.863 | −9.793 | −9.722 | −9.652 | −9.580 | −9.509 |
| 3.913 | 3.906 | 3.899 | 3.890 | 3.880 | 3.869 | 3.856 | 3.843 | 3.829 | 3.814 | 3.799 | 3.783 | 3.766 | 3.749 | 3.732 | 3.714 | 3.695 |

| | | | | | | | | | | | | | | | | |
|---|---|---|---|---|---|---|---|---|---|---|---|---|---|---|---|---|
| 1.911 | 1.913 | 1.915 | 1.916 | 1.918 | 1.920 | 1.921 | 1.923 | 1.925 | 1.926 | 1.928 | 1.930 | 1.931 | 1.933 | 1.935 | 1.936 | 1.938 |
| −8.229 | −8.161 | −8.092 | −8.022 | −7.951 | −7.880 | −7.809 | −7.738 | −7.666 | −7.594 | −7.522 | −7.450 | −7.378 | −7.306 | −7.235 | −7.164 | −7.094 |
| 3.987 | 3.971 | 3.955 | 3.939 | 3.924 | 3.909 | 3.894 | 3.880 | 3.866 | 3.853 | 3.840 | 3.828 | 3.817 | 3.806 | 3.796 | 3.786 | 3.777 |
| −9.438 | −9.368 | −9.297 | −9.228 | −9.159 | −9.091 | −9.023 | −8.956 | −8.890 | −8.825 | −8.761 | −8.698 | −8.636 | −8.575 | −8.515 | −8.456 | −8.398 |
| 3.677 | 3.658 | 3.640 | 3.622 | 3.604 | 3.586 | 3.568 | 3.550 | 3.533 | 3.517 | 3.501 | 3.485 | 3.470 | 3.455 | 3.441 | 3.428 | 3.415 |

| | | | | | | | | | | | | | | | | |
|---|---|---|---|---|---|---|---|---|---|---|---|---|---|---|---|---|
| 1.940 | 1.941 | 1.943 | 1.945 | 1.946 | 1.948 | 1.950 | 1.951 | 1.953 | 1.955 | 1.956 | 1.958 | 1.960 | 1.961 | 1.963 | 1.965 | 1.966 |
| −7.024 | −6.954 | −6.885 | −6.817 | −6.748 | −6.681 | −6.614 | −6.548 | −6.483 | −6.419 | −6.356 | −6.294 | −6.232 | −6.172 | −6.112 | −6.053 | −5.996 |
| 3.768 | 3.760 | 3.753 | 3.746 | 3.740 | 3.734 | 3.729 | 3.724 | 3.720 | 3.716 | 3.713 | 3.710 | 3.708 | 3.706 | 3.705 | 3.705 | 3.706 |
| −8.340 | −8.284 | −8.229 | −8.174 | −8.120 | −8.066 | −8.014 | −7.962 | −7.911 | −7.861 | −7.812 | −7.765 | −7.719 | −7.674 | −7.630 | −7.587 | −7.546 |
| 3.403 | 3.391 | 3.379 | 3.369 | 3.358 | 3.349 | 3.340 | 3.331 | 3.324 | 3.317 | 3.310 | 3.304 | 3.299 | 3.294 | 3.290 | 3.286 | 3.283 |

| | | | | | | | | | | | | | | | | |
|---|---|---|---|---|---|---|---|---|---|---|---|---|---|---|---|---|
| 1.968 | 1.970 | 1.971 | 1.973 | 1.975 | 1.976 | 1.978 | 1.980 | 1.981 | 1.983 | 1.985 | 1.986 | 1.988 | 1.990 | 1.991 | 1.993 | 1.995 |
| −5.940 | −5.885 | −5.831 | −5.779 | −5.728 | −5.678 | −5.630 | −5.583 | −5.537 | −5.494 | −5.452 | −5.411 | −5.372 | −5.334 | −5.298 | −5.264 | −5.232 |
| 3.707 | 3.710 | 3.713 | 3.717 | 3.721 | 3.726 | 3.732 | 3.739 | 3.747 | 3.756 | 3.766 | 3.777 | 3.789 | 3.802 | 3.816 | 3.831 | 3.848 |
| −7.505 | −7.465 | −7.425 | −7.387 | −7.350 | −7.313 | −7.277 | −7.242 | −7.208 | −7.175 | −7.142 | −7.111 | −7.079 | −7.048 | −7.018 | −6.988 | −6.958 |
| 3.281 | 3.279 | 3.278 | 3.278 | 3.279 | 3.281 | 3.284 | 3.288 | 3.293 | 3.299 | 3.305 | 3.313 | 3.323 | 3.333 | 3.344 | 3.356 | 3.370 |

| | | | | | | | | | | | | | | | | |
|---|---|---|---|---|---|---|---|---|---|---|---|---|---|---|---|---|
| 1.996 | 1.998 | 2.000 | 2.001 | 2.003 | 2.005 | 2.006 | 2.008 | 2.010 | 2.011 | 2.013 | 2.015 | 2.016 | 2.018 | 2.020 | 2.021 | 2.023 |
| | | | | | | | | | | | | | | | | |
| −5.202 | −5.173 | −5.147 | −5.122 | −5.099 | −5.078 | −5.059 | −5.041 | −5.025 | −5.010 | −4.997 | −4.986 | −4.976 | −4.969 | −4.965 | −4.962 | −4.962 |
| 3.865 | 3.883 | 3.902 | 3.922 | 3.943 | 3.965 | 3.987 | 4.010 | 4.034 | 4.057 | 4.081 | 4.106 | 4.130 | 4.155 | 4.180 | 4.204 | 4.229 |
| | | | | | | | | | | | | | | | | |
| −6.929 | −6.901 | −6.873 | −6.845 | −6.819 | −6.793 | −6.769 | −6.744 | −6.721 | −6.699 | −6.678 | −6.658 | −6.639 | −6.622 | −6.606 | −6.592 | −6.580 |
| 3.385 | 3.400 | 3.417 | 3.435 | 3.454 | 3.474 | 3.496 | 3.518 | 3.542 | 3.566 | 3.592 | 3.618 | 3.645 | 3.674 | 3.702 | 3.731 | 3.760 |

| | | | | | | | | | | | | | | | | |
|---|---|---|---|---|---|---|---|---|---|---|---|---|---|---|---|---|
| 2.025 | 2.026 | 2.028 | 2.030 | 2.031 | 2.033 | 2.035 | 2.036 | 2.038 | 2.040 | 2.041 | 2.043 | 2.045 | 2.046 | 2.048 | 2.050 | 2.051 |
| −4.965 | −4.970 | −4.977 | −4.987 | −4.998 | −5.012 | −5.029 | −5.048 | −5.069 | −5.092 | −5.116 | −5.143 | −5.172 | −5.202 | −5.233 | −5.266 | −5.299 |
| 4.253 | 4.277 | 4.301 | 4.325 | 4.347 | 4.370 | 4.391 | 4.411 | 4.430 | 4.448 | 4.465 | 4.480 | 4.494 | 4.506 | 4.516 | 4.524 | 4.531 |
| −6.569 | −6.559 | −6.551 | −6.545 | −6.541 | −6.538 | −6.537 | −6.537 | −6.539 | −6.542 | −6.546 | −6.551 | −6.557 | −6.564 | −6.572 | −6.581 | −6.591 |
| 3.790 | 3.819 | 3.848 | 3.877 | 3.905 | 3.933 | 3.960 | 3.986 | 4.011 | 4.035 | 4.058 | 4.079 | 4.099 | 4.118 | 4.134 | 4.150 | 4.163 |

| | | | | | | | | | | | | | | | | |
|---|---|---|---|---|---|---|---|---|---|---|---|---|---|---|---|---|
| 2.053 | 2.055 | 2.056 | 2.058 | 2.060 | 2.061 | 2.063 | 2.065 | 2.066 | 2.068 | 2.070 | 2.071 | 2.073 | 2.075 | 2.076 | 2.078 | 2.080 |
| −5.334 | −5.369 | −5.405 | −5.441 | −5.477 | −5.513 | −5.550 | −5.587 | −5.624 | −5.662 | −5.700 | −5.739 | −5.779 | −5.819 | −5.859 | −5.900 | −5.942 |
| 4.536 | 4.539 | 4.540 | 4.540 | 4.537 | 4.533 | 4.526 | 4.518 | 4.507 | 4.493 | 4.477 | 4.458 | 4.436 | 4.411 | 4.384 | 4.353 | 4.320 |
| −6.600 | −6.611 | −6.621 | −6.632 | −6.642 | −6.653 | −6.663 | −6.674 | −6.685 | −6.697 | −6.709 | −6.721 | −6.734 | −6.748 | −6.762 | −6.776 | −6.791 |
| 4.175 | 4.185 | 4.193 | 4.200 | 4.204 | 4.207 | 4.208 | 4.208 | 4.205 | 4.200 | 4.193 | 4.184 | 4.172 | 4.159 | 4.143 | 4.125 | 4.105 |

| | | | | | | | | | | | | | | | | |
|---|---|---|---|---|---|---|---|---|---|---|---|---|---|---|---|---|
| 2.081 | 2.083 | 2.085 | 2.086 | 2.088 | 2.090 | 2.091 | 2.093 | 2.095 | 2.096 | 2.098 | 2.100 | 2.101 | 2.103 | 2.105 | 2.106 | 2.108 |
| −5.984 | −6.026 | −6.068 | −6.110 | −6.152 | −6.194 | −6.237 | −6.279 | −6.320 | −6.361 | −6.401 | −6.440 | −6.478 | −6.514 | −6.549 | −6.582 | −6.612 |
| 4.284 | 4.245 | 4.204 | 4.160 | 4.113 | 4.064 | 4.012 | 3.958 | 3.902 | 3.843 | 3.782 | 3.719 | 3.653 | 3.586 | 3.517 | 3.446 | 3.373 |
| −6.807 | −6.824 | −6.842 | −6.861 | −6.881 | −6.902 | −6.923 | −6.946 | −6.968 | −6.992 | −7.016 | −7.041 | −7.067 | −7.093 | −7.119 | −7.146 | −7.174 |
| 4.082 | 4.058 | 4.030 | 4.000 | 3.968 | 3.933 | 3.895 | 3.855 | 3.812 | 3.767 | 3.720 | 3.670 | 3.619 | 3.565 | 3.509 | 3.451 | 3.391 |

| | | | | | | | | | | | | | | | | |
|---|---|---|---|---|---|---|---|---|---|---|---|---|---|---|---|---|
| 2.110 | 2.111 | 2.113 | 2.114 | 2.116 | 2.118 | 2.119 | 2.121 | 2.123 | 2.124 | 2.126 | 2.128 | 2.129 | 2.131 | 2.133 | 2.134 | 2.136 |
| −6.641 | −6.668 | −6.693 | −6.717 | −6.739 | −6.759 | −6.779 | −6.796 | −6.813 | −6.828 | −6.842 | −6.855 | −6.867 | −6.878 | −6.888 | −6.898 | −6.906 |
| 3.298 | 3.221 | 3.143 | 3.063 | 2.981 | 2.898 | 2.813 | 2.727 | 2.640 | 2.552 | 2.463 | 2.373 | 2.283 | 2.193 | 2.102 | 2.011 | 1.920 |
| −7.201 | −7.229 | −7.258 | −7.287 | −7.316 | −7.346 | −7.377 | −7.408 | −7.440 | −7.472 | −7.504 | −7.536 | −7.568 | −7.600 | −7.632 | −7.663 | −7.694 |
| 3.329 | 3.265 | 3.199 | 3.131 | 3.061 | 2.990 | 2.917 | 2.843 | 2.767 | 2.690 | 2.612 | 2.533 | 2.453 | 2.372 | 2.290 | 2.207 | 2.124 |

| | | | | | | | | | | | | | | | | |
|---|---|---|---|---|---|---|---|---|---|---|---|---|---|---|---|---|
| 2.138 | 2.139 | 2.141 | 2.143 | 2.144 | 2.146 | 2.148 | 2.149 | 2.151 | 2.153 | 2.154 | 2.156 | 2.158 | 2.159 | 2.161 | 2.163 | 2.164 |
| | | | | | | | | | | | | | | | | |
| −6.914 | −6.921 | −6.927 | −6.933 | −6.938 | −6.944 | −6.949 | −6.954 | −6.959 | −6.964 | −6.970 | −6.976 | −6.982 | −6.989 | −6.996 | −7.003 | −7.011 |
| 1.829 | 1.738 | 1.647 | 1.556 | 1.466 | 1.377 | 1.287 | 1.198 | 1.110 | 1.023 | 0.936 | 0.850 | 0.766 | 0.683 | 0.602 | 0.522 | 0.443 |
| | | | | | | | | | | | | | | | | |
| −7.724 | −7.755 | −7.785 | −7.814 | −7.844 | −7.873 | −7.902 | −7.930 | −7.958 | −7.986 | −8.013 | −8.040 | −8.067 | −8.093 | −8.119 | −8.145 | −8.170 |
| 2.039 | 1.955 | 1.869 | 1.784 | 1.698 | 1.612 | 1.525 | 1.439 | 1.353 | 1.266 | 1.180 | 1.094 | 1.009 | 0.923 | 0.838 | 0.754 | 0.670 |

| | | | | | | | | | | | | | | | | |
|---|---|---|---|---|---|---|---|---|---|---|---|---|---|---|---|---|
| 2.166 | 2.168 | 2.169 | 2.171 | 2.173 | 2.174 | 2.176 | 2.178 | 2.179 | 2.181 | 2.183 | 2.184 | 2.186 | 2.188 | 2.189 | 2.191 | 2.193 |
| −7.019 | −7.027 | −7.036 | −7.045 | −7.055 | −7.065 | −7.075 | −7.086 | −7.097 | −7.108 | −7.119 | −7.131 | −7.144 | −7.157 | −7.171 | −7.185 | −7.200 |
| 0.366 | 0.291 | 0.217 | 0.145 | 0.075 | 0.006 | −0.060 | −0.124 | −0.186 | −0.246 | −0.303 | −0.358 | −0.411 | −0.462 | −0.510 | −0.556 | −0.599 |
| −8.196 | −8.221 | −8.247 | −8.272 | −8.297 | −8.323 | −8.348 | −8.373 | −8.398 | −8.423 | −8.448 | −8.472 | −8.496 | −8.520 | −8.544 | −8.567 | −8.590 |
| 0.587 | 0.505 | 0.424 | 0.343 | 0.264 | 0.187 | 0.110 | 0.035 | −0.039 | −0.111 | −0.181 | −0.250 | −0.317 | −0.381 | −0.445 | −0.506 | −0.565 |

| | | | | | | | | | | | | | | | | |
|---|---|---|---|---|---|---|---|---|---|---|---|---|---|---|---|---|
| 2.194 | 2.196 | 2.198 | 2.199 | 2.201 | 2.203 | 2.204 | 2.206 | 2.208 | 2.209 | 2.211 | 2.213 | 2.214 | 2.216 | 2.218 | 2.219 | 2.221 |
| | | | | | | | | | | | | | | | | |
| −7.216 | −7.232 | −7.249 | −7.266 | −7.283 | −7.300 | −7.318 | −7.336 | −7.354 | −7.373 | −7.392 | −7.412 | −7.431 | −7.451 | −7.471 | −7.492 | −7.512 |
| −0.640 | −0.679 | −0.715 | −0.749 | −0.781 | −0.811 | −0.838 | −0.863 | −0.886 | −0.908 | −0.927 | −0.944 | −0.959 | −0.973 | −0.984 | −0.994 | −1.001 |
| | | | | | | | | | | | | | | | | |
| −8.613 | −8.636 | −8.658 | −8.681 | −8.703 | −8.725 | −8.748 | −8.770 | −8.793 | −8.816 | −8.840 | −8.863 | −8.887 | −8.910 | −8.933 | −8.956 | −8.978 |
| −0.622 | −0.677 | −0.730 | −0.780 | −0.828 | −0.874 | −0.917 | −0.958 | −0.996 | −1.031 | −1.063 | −1.092 | −1.117 | −1.140 | −1.160 | −1.177 | −1.191 |

| | | | | | | | | | | | | | | | | |
|---|---|---|---|---|---|---|---|---|---|---|---|---|---|---|---|---|
| 2.223 | 2.224 | 2.226 | 2.228 | 2.229 | 2.231 | 2.233 | 2.234 | 2.236 | 2.238 | 2.239 | 2.241 | 2.243 | 2.244 | 2.246 | 2.248 | 2.249 |
| −7.533 | −7.553 | −7.574 | −7.595 | −7.615 | −7.635 | −7.655 | −7.675 | −7.695 | −7.715 | −7.736 | −7.756 | −7.777 | −7.798 | −7.820 | −7.843 | −7.866 |
| −1.007 | −1.011 | −1.012 | −1.012 | −1.010 | −1.005 | −0.999 | −0.990 | −0.979 | −0.967 | −0.953 | −0.936 | −0.918 | −0.898 | −0.876 | −0.853 | −0.827 |
| −9.001 | −9.023 | −9.046 | −9.068 | −9.091 | −9.114 | −9.138 | −9.163 | −9.188 | −9.215 | −9.242 | −9.270 | −9.299 | −9.329 | −9.359 | −9.390 | −9.421 |
| −1.202 | −1.210 | −1.215 | −1.217 | −1.217 | −1.215 | −1.210 | −1.202 | −1.192 | −1.181 | −1.167 | −1.151 | −1.133 | −1.113 | −1.091 | −1.068 | −1.043 |

| | | | | | | | | | | | | | | | | |
|---|---|---|---|---|---|---|---|---|---|---|---|---|---|---|---|---|
| 2.251 | 2.253 | 2.254 | 2.256 | 2.258 | 2.259 | 2.261 | 2.263 | 2.264 | 2.266 | 2.268 | 2.269 | 2.271 | 2.273 | 2.274 | 2.276 | 2.278 |
| −7.890 | −7.914 | −7.939 | −7.965 | −7.992 | −8.019 | −8.047 | −8.075 | −8.104 | −8.134 | −8.164 | −8.194 | −8.225 | −8.256 | −8.288 | −8.319 | −8.350 |
| −0.800 | −0.771 | −0.740 | −0.708 | −0.675 | −0.640 | −0.604 | −0.566 | −0.528 | −0.488 | −0.447 | −0.406 | −0.365 | −0.322 | −0.280 | −0.237 | −0.193 |
| −9.452 | −9.483 | −9.515 | −9.546 | −9.576 | −9.607 | −9.637 | −9.666 | −9.696 | −9.724 | −9.752 | −9.780 | −9.807 | −9.834 | −9.860 | −9.885 | −9.911 |
| −1.017 | −0.988 | −0.958 | −0.926 | −0.892 | −0.857 | −0.821 | −0.783 | −0.744 | −0.704 | −0.663 | −0.621 | −0.579 | −0.536 | −0.492 | −0.448 | −0.404 |

| | | | | | | | | | | | | | | | | |
|---|---|---|---|---|---|---|---|---|---|---|---|---|---|---|---|---|
| 2.279 | 2.281 | 2.283 | 2.284 | 2.286 | 2.288 | 2.289 | 2.291 | 2.293 | 2.294 | 2.296 | 2.298 | 2.299 | 2.301 | 2.303 | 2.304 | 2.306 |
| −8.381 | −8.412 | −8.442 | −8.473 | −8.502 | −8.532 | −8.561 | −8.589 | −8.618 | −8.645 | −8.672 | −8.698 | −8.724 | −8.749 | −8.773 | −8.796 | −8.818 |
| −0.150 | −0.107 | −0.063 | −0.020 | 0.024 | 0.067 | 0.110 | 0.153 | 0.196 | 0.239 | 0.282 | 0.324 | 0.367 | 0.409 | 0.451 | 0.493 | 0.536 |
| −9.935 | −9.960 | −9.983 | −10.006 | −10.029 | −10.051 | −10.072 | −10.093 | −10.113 | −10.132 | −10.149 | −10.166 | −10.182 | −10.198 | −10.213 | −10.227 | −10.241 |
| −0.360 | −0.316 | −0.272 | −0.227 | −0.183 | −0.138 | −0.094 | −0.050 | −0.005 | 0.039 | 0.083 | 0.127 | 0.171 | 0.214 | 0.257 | 0.300 | 0.343 |

| | | | | | | | | | | | | | | | | |
|---|---|---|---|---|---|---|---|---|---|---|---|---|---|---|---|---|
| 2.308 | 2.309 | 2.311 | 2.313 | 2.314 | 2.316 | 2.318 | 2.319 | 2.321 | 2.323 | 2.324 | 2.326 | 2.328 | 2.329 | 2.331 | 2.333 | 2.334 |
| | | | | | | | | | | | | | | | | |
| −8.839 | −8.859 | −8.877 | −8.894 | −8.908 | −8.921 | −8.932 | −8.942 | −8.950 | −8.956 | −8.961 | −8.965 | −8.967 | −8.968 | −8.967 | −8.965 | −8.962 |
| 0.578 | 0.621 | 0.663 | 0.705 | 0.748 | 0.790 | 0.832 | 0.874 | 0.916 | 0.958 | 0.999 | 1.040 | 1.080 | 1.119 | 1.158 | 1.195 | 1.232 |
| | | | | | | | | | | | | | | | | |
| −10.254 | −10.267 | −10.279 | −10.291 | −10.302 | −10.312 | −10.322 | −10.331 | −10.340 | −10.349 | −10.358 | −10.366 | −10.374 | −10.382 | −10.390 | −10.398 | −10.406 |
| 0.385 | 0.427 | 0.468 | 0.508 | 0.548 | 0.586 | 0.624 | 0.660 | 0.696 | 0.730 | 0.763 | 0.795 | 0.826 | 0.856 | 0.884 | 0.911 | 0.936 |

| | | | | | | | | | | | | | | | | |
|---|---|---|---|---|---|---|---|---|---|---|---|---|---|---|---|---|
| 2.336 | 2.338 | 2.339 | 2.341 | 2.343 | 2.344 | 2.346 | 2.348 | 2.349 | 2.351 | 2.353 | 2.354 | 2.356 | 2.358 | 2.359 | 2.361 | 2.363 |
| −8.958 | −8.953 | −8.947 | −8.940 | −8.932 | −8.924 | −8.915 | −8.906 | −8.896 | −8.887 | −8.877 | −8.866 | −8.856 | −8.845 | −8.833 | −8.821 | −8.809 |
| 1.268 | 1.303 | 1.338 | 1.371 | 1.403 | 1.434 | 1.465 | 1.494 | 1.523 | 1.551 | 1.577 | 1.603 | 1.627 | 1.651 | 1.673 | 1.695 | 1.716 |
| −10.414 | −10.423 | −10.431 | −10.439 | −10.446 | −10.454 | −10.460 | −10.466 | −10.472 | −10.476 | −10.480 | −10.484 | −10.486 | −10.488 | −10.489 | −10.490 | −10.491 |
| 0.961 | 0.984 | 1.007 | 1.029 | 1.050 | 1.070 | 1.090 | 1.110 | 1.129 | 1.148 | 1.167 | 1.185 | 1.203 | 1.220 | 1.237 | 1.253 | 1.269 |

| | | | | | | | | | | | | | | | | |
|---|---|---|---|---|---|---|---|---|---|---|---|---|---|---|---|---|
| 2.364 | 2.366 | 2.368 | 2.369 | 2.371 | 2.373 | 2.374 | 2.376 | 2.378 | 2.379 | 2.381 | 2.383 | 2.384 | 2.386 | 2.388 | 2.389 | 2.391 |
| −8.797 | −8.785 | −8.773 | −8.761 | −8.749 | −8.737 | −8.725 | −8.713 | −8.702 | −8.691 | −8.681 | −8.672 | −8.663 | −8.655 | −8.649 | −8.643 | −8.638 |
| 1.736 | 1.755 | 1.773 | 1.790 | 1.807 | 1.823 | 1.837 | 1.851 | 1.865 | 1.877 | 1.889 | 1.901 | 1.912 | 1.923 | 1.933 | 1.943 | 1.952 |
| −10.492 | −10.493 | −10.495 | −10.497 | −10.499 | −10.502 | −10.505 | −10.509 | −10.513 | −10.519 | −10.524 | −10.530 | −10.537 | −10.545 | −10.553 | −10.563 | −10.572 |
| 1.284 | 1.299 | 1.312 | 1.326 | 1.338 | 1.351 | 1.362 | 1.374 | 1.384 | 1.395 | 1.405 | 1.414 | 1.423 | 1.432 | 1.440 | 1.447 | 1.454 |

| | | | | | | | | | | | | | | | | |
|---|---|---|---|---|---|---|---|---|---|---|---|---|---|---|---|---|
| 2.393 | 2.394 | 2.396 | 2.398 | 2.399 | 2.401 | 2.403 | 2.404 | 2.406 | 2.408 | 2.409 | 2.411 | 2.413 | 2.414 | 2.416 | 2.418 | 2.419 |
| −8.635 | −8.631 | −8.629 | −8.627 | −8.626 | −8.625 | −8.624 | −8.624 | −8.624 | −8.624 | −8.625 | −8.625 | −8.626 | −8.627 | −8.628 | −8.630 | −8.633 |
| 1.962 | 1.971 | 1.979 | 1.988 | 1.996 | 2.004 | 2.012 | 2.019 | 2.027 | 2.034 | 2.041 | 2.049 | 2.056 | 2.064 | 2.072 | 2.080 | 2.088 |
| −10.583 | −10.594 | −10.606 | −10.618 | −10.631 | −10.644 | −10.657 | −10.671 | −10.685 | −10.700 | −10.715 | −10.730 | −10.746 | −10.761 | −10.777 | −10.794 | −10.810 |
| 1.461 | 1.468 | 1.474 | 1.481 | 1.487 | 1.493 | 1.500 | 1.506 | 1.512 | 1.519 | 1.526 | 1.532 | 1.539 | 1.546 | 1.552 | 1.559 | 1.565 |

| | | | | | | | | | | | | | | | | |
|---|---|---|---|---|---|---|---|---|---|---|---|---|---|---|---|---|
| 2.421 | 2.423 | 2.424 | 2.426 | 2.428 | 2.429 | 2.431 | 2.433 | 2.434 | 2.436 | 2.438 | 2.439 | 2.441 | 2.442 | 2.444 | 2.446 | 2.447 |
| −8.636 | −8.639 | −8.643 | −8.647 | −8.652 | −8.657 | −8.662 | −8.668 | −8.673 | −8.679 | −8.685 | −8.692 | −8.699 | −8.706 | −8.714 | −8.722 | −8.731 |
| 2.096 | 2.104 | 2.112 | 2.120 | 2.128 | 2.136 | 2.144 | 2.152 | 2.159 | 2.167 | 2.175 | 2.183 | 2.190 | 2.198 | 2.206 | 2.214 | 2.223 |
| −10.827 | −10.843 | −10.860 | −10.877 | −10.893 | −10.910 | −10.927 | −10.944 | −10.960 | −10.977 | −10.994 | −11.010 | −11.026 | −11.043 | −11.058 | −11.074 | −11.089 |
| 1.572 | 1.578 | 1.584 | 1.590 | 1.595 | 1.600 | 1.606 | 1.610 | 1.615 | 1.620 | 1.624 | 1.629 | 1.633 | 1.636 | 1.640 | 1.644 | 1.648 |

| | | | | | | | | | | | | | | | | |
|---|---|---|---|---|---|---|---|---|---|---|---|---|---|---|---|---|
| 2.449 | 2.451 | 2.452 | 2.454 | 2.456 | 2.457 | 2.459 | 2.461 | 2.462 | 2.464 | 2.466 | 2.467 | 2.469 | 2.471 | 2.472 | 2.474 | 2.476 |
| −8.740 | −8.749 | −8.759 | −8.769 | −8.780 | −8.791 | −8.802 | −8.813 | −8.825 | −8.837 | −8.848 | −8.861 | −8.873 | −8.885 | −8.897 | −8.909 | −8.921 |
| 2.231 | 2.240 | 2.250 | 2.260 | 2.270 | 2.280 | 2.291 | 2.302 | 2.314 | 2.325 | 2.337 | 2.349 | 2.361 | 2.373 | 2.385 | 2.397 | 2.409 |
| −11.104 | −11.118 | −11.131 | −11.144 | −11.157 | −11.168 | −11.179 | −11.189 | −11.198 | −11.206 | −11.214 | −11.220 | −11.225 | −11.230 | −11.233 | −11.234 | −11.234 |
| 1.652 | 1.656 | 1.661 | 1.666 | 1.671 | 1.677 | 1.683 | 1.689 | 1.696 | 1.703 | 1.711 | 1.719 | 1.727 | 1.735 | 1.743 | 1.752 | 1.761 |

| | | | | | | | | | | | | | | | | |
|---|---|---|---|---|---|---|---|---|---|---|---|---|---|---|---|---|
| 2.477 | 2.479 | 2.481 | 2.482 | 2.484 | 2.486 | 2.487 | 2.489 | 2.491 | 2.492 | 2.494 | 2.496 | 2.497 | 2.499 | 2.501 | 2.502 | 2.504 |
| | | | | | | | | | | | | | | | | |
| −8.931 | −8.942 | −8.951 | −8.958 | −8.965 | −8.971 | −8.975 | −8.979 | −8.981 | −8.983 | −8.983 | −8.982 | −8.980 | −8.978 | −8.974 | −8.969 | −8.963 |
| 2.421 | 2.433 | 2.445 | 2.456 | 2.468 | 2.480 | 2.491 | 2.503 | 2.514 | 2.526 | 2.537 | 2.547 | 2.558 | 2.569 | 2.579 | 2.588 | 2.598 |
| | | | | | | | | | | | | | | | | |
| −11.233 | −11.230 | −11.224 | −11.217 | −11.207 | −11.196 | −11.182 | −11.167 | −11.149 | −11.130 | −11.109 | −11.086 | −11.061 | −11.035 | −11.008 | −10.978 | −10.948 |
| 1.770 | 1.778 | 1.787 | 1.796 | 1.805 | 1.814 | 1.823 | 1.831 | 1.840 | 1.848 | 1.856 | 1.864 | 1.871 | 1.878 | 1.886 | 1.892 | 1.899 |

| | | | | | | | | | | | | | | | | |
|---|---|---|---|---|---|---|---|---|---|---|---|---|---|---|---|---|
| 2.506 | 2.507 | 2.509 | 2.511 | 2.512 | 2.514 | 2.516 | 2.517 | 2.519 | 2.521 | 2.522 | 2.524 | 2.526 | 2.527 | 2.529 | 2.531 | 2.532 |
| −8.957 | −8.950 | −8.941 | −8.932 | −8.922 | −8.911 | −8.899 | −8.887 | −8.873 | −8.858 | −8.842 | −8.826 | −8.808 | −8.790 | −8.771 | −8.752 | −8.731 |
| 2.608 | 2.617 | 2.626 | 2.635 | 2.644 | 2.654 | 2.663 | 2.672 | 2.682 | 2.692 | 2.702 | 2.712 | 2.722 | 2.732 | 2.742 | 2.753 | 2.763 |
| −10.915 | −10.882 | −10.847 | −10.812 | −10.776 | −10.739 | −10.701 | −10.663 | −10.624 | −10.586 | −10.547 | −10.508 | −10.469 | −10.429 | −10.390 | −10.351 | −10.311 |
| 1.905 | 1.910 | 1.916 | 1.920 | 1.925 | 1.929 | 1.933 | 1.937 | 1.940 | 1.943 | 1.946 | 1.949 | 1.952 | 1.955 | 1.957 | 1.960 | 1.962 |

| | | | | | | | | | | | | | | | | |
|---|---|---|---|---|---|---|---|---|---|---|---|---|---|---|---|---|
| 2.534 | 2.536 | 2.537 | 2.539 | 2.541 | 2.542 | 2.544 | 2.546 | 2.547 | 2.549 | 2.551 | 2.552 | 2.554 | 2.556 | 2.557 | 2.559 | 2.561 |
| | | | | | | | | | | | | | | | | |
| −8.709 | −8.686 | −8.662 | −8.636 | −8.609 | −8.580 | −8.549 | −8.517 | −8.483 | −8.448 | −8.412 | −8.375 | −8.338 | −8.299 | −8.260 | −8.220 | −8.180 |
| 2.774 | 2.785 | 2.796 | 2.807 | 2.818 | 2.829 | 2.841 | 2.853 | 2.865 | 2.877 | 2.890 | 2.903 | 2.916 | 2.929 | 2.943 | 2.957 | 2.971 |
| | | | | | | | | | | | | | | | | |
| −10.272 | −10.232 | −10.192 | −10.152 | −10.112 | −10.071 | −10.031 | −9.990 | −9.948 | −9.906 | −9.864 | −9.821 | −9.777 | −9.733 | −9.688 | −9.643 | −9.597 |
| 1.964 | 1.966 | 1.967 | 1.968 | 1.969 | 1.970 | 1.970 | 1.970 | 1.970 | 1.970 | 1.971 | 1.971 | 1.971 | 1.972 | 1.972 | 1.972 | 1.973 |

| | | | | | | | | | | | | | | | | |
|---|---|---|---|---|---|---|---|---|---|---|---|---|---|---|---|---|
| 2.562 | 2.564 | 2.566 | 2.567 | 2.569 | 2.571 | 2.572 | 2.574 | 2.576 | 2.577 | 2.579 | 2.581 | 2.582 | 2.584 | 2.586 | 2.587 | 2.589 |
| −8.140 | −8.099 | −8.059 | −8.018 | −7.977 | −7.936 | −7.895 | −7.853 | −7.811 | −7.769 | −7.727 | −7.685 | −7.642 | −7.598 | −7.555 | −7.510 | −7.466 |
| 2.986 | 3.001 | 3.017 | 3.033 | 3.050 | 3.067 | 3.084 | 3.102 | 3.121 | 3.140 | 3.159 | 3.178 | 3.198 | 3.217 | 3.237 | 3.256 | 3.276 |
| −9.550 | −9.502 | −9.454 | −9.405 | −9.355 | −9.305 | −9.255 | −9.203 | −9.151 | −9.099 | −9.046 | −8.992 | −8.939 | −8.885 | −8.831 | −8.777 | −8.722 |
| 1.973 | 1.973 | 1.973 | 1.973 | 1.974 | 1.974 | 1.974 | 1.974 | 1.974 | 1.974 | 1.973 | 1.972 | 1.972 | 1.971 | 1.971 | 1.970 | 1.970 |

| | | | | | | | | | | | | | | | | |
|---|---|---|---|---|---|---|---|---|---|---|---|---|---|---|---|---|
| 2.591 | 2.592 | 2.594 | 2.596 | 2.597 | 2.599 | 2.601 | 2.602 | 2.604 | 2.606 | 2.607 | 2.609 | 2.611 | 2.612 | 2.614 | 2.616 | 2.617 |
| −7.421 | −7.375 | −7.329 | −7.283 | −7.237 | −7.191 | −7.145 | −7.099 | −7.054 | −7.009 | −6.965 | −6.922 | −6.879 | −6.836 | −6.795 | −6.754 | −6.714 |
| 3.296 | 3.315 | 3.334 | 3.354 | 3.373 | 3.392 | 3.411 | 3.430 | 3.448 | 3.466 | 3.484 | 3.501 | 3.517 | 3.533 | 3.549 | 3.564 | 3.578 |
| −8.667 | −8.612 | −8.557 | −8.502 | −8.448 | −8.394 | −8.341 | −8.288 | −8.236 | −8.185 | −8.134 | −8.084 | −8.035 | −7.986 | −7.938 | −7.890 | −7.843 |
| 1.971 | 1.972 | 1.973 | 1.974 | 1.976 | 1.979 | 1.982 | 1.986 | 1.990 | 1.995 | 2.001 | 2.007 | 2.013 | 2.020 | 2.028 | 2.035 | 2.043 |

| | | | | | | | | | | | | | | | | |
|---|---|---|---|---|---|---|---|---|---|---|---|---|---|---|---|---|
| 2.619 | 2.621 | 2.622 | 2.624 | 2.626 | 2.627 | 2.629 | 2.631 | 2.632 | 2.634 | 2.636 | 2.637 | 2.639 | 2.641 | 2.642 | 2.644 | 2.646 |
| −6.674 | −6.636 | −6.598 | −6.561 | −6.525 | −6.489 | −6.455 | −6.421 | −6.388 | −6.355 | −6.324 | −6.292 | −6.262 | −6.232 | −6.202 | −6.173 | −6.144 |
| 3.592 | 3.606 | 3.619 | 3.631 | 3.643 | 3.655 | 3.666 | 3.676 | 3.686 | 3.695 | 3.703 | 3.712 | 3.719 | 3.727 | 3.733 | 3.740 | 3.746 |
| −7.797 | −7.750 | −7.704 | −7.658 | −7.612 | −7.566 | −7.521 | −7.476 | −7.431 | −7.386 | −7.341 | −7.297 | −7.253 | −7.208 | −7.164 | −7.120 | −7.076 |
| 2.051 | 2.060 | 2.069 | 2.079 | 2.089 | 2.099 | 2.109 | 2.120 | 2.132 | 2.143 | 2.155 | 2.167 | 2.179 | 2.192 | 2.204 | 2.217 | 2.230 |

| | | | | | | | | | | | | | | | | |
|---|---|---|---|---|---|---|---|---|---|---|---|---|---|---|---|---|
| 2.647 | 2.649 | 2.651 | 2.652 | 2.654 | 2.656 | 2.657 | 2.659 | 2.661 | 2.662 | 2.664 | 2.666 | 2.667 | 2.669 | 2.671 | 2.672 | 2.674 |
| | | | | | | | | | | | | | | | | |
| −6.115 | −6.087 | −6.058 | −6.029 | −6.000 | −5.972 | −5.944 | −5.917 | −5.891 | −5.865 | −5.840 | −5.815 | −5.791 | −5.767 | −5.744 | −5.721 | −5.699 |
| 3.751 | 3.756 | 3.761 | 3.766 | 3.771 | 3.775 | 3.779 | 3.783 | 3.785 | 3.788 | 3.789 | 3.790 | 3.791 | 3.790 | 3.789 | 3.787 | 3.785 |
| | | | | | | | | | | | | | | | | |
| −7.031 | −6.987 | −6.941 | −6.896 | −6.850 | −6.805 | −6.759 | −6.713 | −6.667 | −6.621 | −6.575 | −6.529 | −6.483 | −6.438 | −6.392 | −6.347 | −6.302 |
| 2.243 | 2.256 | 2.269 | 2.282 | 2.296 | 2.309 | 2.323 | 2.336 | 2.350 | 2.364 | 2.377 | 2.391 | 2.404 | 2.418 | 2.431 | 2.444 | 2.456 |

| | | | | | | | | | | | | | | | | |
|---|---|---|---|---|---|---|---|---|---|---|---|---|---|---|---|---|
| 2.676 | 2.677 | 2.679 | 2.681 | 2.682 | 2.684 | 2.686 | 2.687 | 2.689 | 2.691 | 2.692 | 2.694 | 2.696 | 2.697 | 2.699 | 2.701 | 2.702 |
| | | | | | | | | | | | | | | | | |
| −5.678 | −5.656 | −5.636 | −5.615 | −5.596 | −5.577 | −5.558 | −5.541 | −5.524 | −5.508 | −5.494 | −5.481 | −5.470 | −5.460 | −5.452 | −5.446 | −5.442 |
| 3.781 | 3.776 | 3.770 | 3.763 | 3.755 | 3.745 | 3.734 | 3.722 | 3.708 | 3.693 | 3.676 | 3.658 | 3.638 | 3.616 | 3.592 | 3.566 | 3.539 |
| | | | | | | | | | | | | | | | | |
| −6.258 | −6.215 | −6.171 | −6.128 | −6.086 | −6.043 | −6.002 | −5.960 | −5.920 | −5.880 | −5.842 | −5.805 | −5.770 | −5.736 | −5.703 | −5.672 | −5.643 |
| 2.469 | 2.481 | 2.493 | 2.504 | 2.515 | 2.525 | 2.534 | 2.542 | 2.550 | 2.557 | 2.563 | 2.568 | 2.572 | 2.575 | 2.577 | 2.577 | 2.577 |

| | | | | | | | | | | | | | | | | |
|---|---|---|---|---|---|---|---|---|---|---|---|---|---|---|---|---|
| 2.704 | 2.706 | 2.707 | 2.709 | 2.711 | 2.712 | 2.714 | 2.716 | 2.717 | 2.719 | 2.721 | 2.722 | 2.724 | 2.726 | 2.727 | 2.729 | 2.731 |
| | | | | | | | | | | | | | | | | |
| −5.440 | −5.439 | −5.440 | −5.443 | −5.447 | −5.453 | −5.461 | −5.471 | −5.483 | −5.497 | −5.513 | −5.531 | −5.551 | −5.573 | −5.597 | −5.623 | −5.650 |
| 3.509 | 3.477 | 3.442 | 3.406 | 3.368 | 3.328 | 3.285 | 3.240 | 3.193 | 3.144 | 3.093 | 3.040 | 2.986 | 2.929 | 2.871 | 2.812 | 2.751 |
| | | | | | | | | | | | | | | | | |
| −5.616 | −5.591 | −5.568 | −5.546 | −5.527 | −5.509 | −5.494 | −5.480 | −5.469 | −5.459 | −5.452 | −5.447 | −5.444 | −5.444 | −5.446 | −5.450 | −5.457 |
| 2.575 | 2.571 | 2.566 | 2.559 | 2.551 | 2.540 | 2.529 | 2.515 | 2.499 | 2.482 | 2.462 | 2.440 | 2.417 | 2.391 | 2.364 | 2.334 | 2.303 |

| | | | | | | | | | | | | | | | | |
|---|---|---|---|---|---|---|---|---|---|---|---|---|---|---|---|---|
| 2.732 | 2.734 | 2.736 | 2.737 | 2.739 | 2.741 | 2.742 | 2.744 | 2.746 | 2.747 | 2.749 | 2.751 | 2.752 | 2.754 | 2.756 | 2.757 | 2.759 |
| −5.680 | −5.712 | −5.746 | −5.782 | −5.819 | −5.858 | −5.899 | −5.940 | −5.983 | −6.027 | −6.073 | −6.120 | −6.169 | −6.220 | −6.273 | −6.327 | −6.384 |
| 2.688 | 2.625 | 2.560 | 2.494 | 2.427 | 2.359 | 2.289 | 2.219 | 2.147 | 2.075 | 2.002 | 1.928 | 1.853 | 1.779 | 1.703 | 1.628 | 1.552 |
| −5.467 | −5.479 | −5.494 | −5.510 | −5.529 | −5.550 | −5.573 | −5.598 | −5.625 | −5.654 | −5.685 | −5.718 | −5.754 | −5.792 | −5.832 | −5.876 | −5.921 |
| 2.270 | 2.236 | 2.201 | 2.164 | 2.126 | 2.087 | 2.048 | 2.007 | 1.966 | 1.923 | 1.880 | 1.836 | 1.791 | 1.746 | 1.700 | 1.654 | 1.608 |

| | | | | | | | | | | | | | | | | |
|---|---|---|---|---|---|---|---|---|---|---|---|---|---|---|---|---|
| 2.761 | 2.762 | 2.764 | 2.765 | 2.767 | 2.769 | 2.770 | 2.772 | 2.774 | 2.775 | 2.777 | 2.779 | 2.780 | 2.782 | 2.784 | 2.785 | 2.787 |
| −6.443 | −6.503 | −6.566 | −6.630 | −6.696 | −6.764 | −6.834 | −6.905 | −6.978 | −7.051 | −7.126 | −7.202 | −7.279 | −7.357 | −7.436 | −7.516 | −7.597 |
| 1.477 | 1.401 | 1.325 | 1.249 | 1.173 | 1.098 | 1.022 | 0.947 | 0.873 | 0.799 | 0.725 | 0.652 | 0.579 | 0.507 | 0.435 | 0.365 | 0.294 |
| −5.970 | −6.020 | −6.074 | −6.130 | −6.189 | −6.251 | −6.315 | −6.382 | −6.452 | −6.525 | −6.600 | −6.677 | −6.757 | −6.839 | −6.922 | −7.008 | −7.095 |
| 1.561 | 1.514 | 1.467 | 1.419 | 1.371 | 1.322 | 1.273 | 1.223 | 1.173 | 1.122 | 1.071 | 1.020 | 0.969 | 0.917 | 0.865 | 0.813 | 0.760 |

| | | | | | | | | | | | | | | | | |
|---|---|---|---|---|---|---|---|---|---|---|---|---|---|---|---|---|
| 2.789 | 2.790 | 2.792 | 2.794 | 2.795 | 2.797 | 2.799 | 2.800 | 2.802 | 2.804 | 2.805 | 2.807 | 2.809 | 2.810 | 2.812 | 2.814 | 2.815 |
| | | | | | | | | | | | | | | | | |
| −7.678 | −7.759 | −7.840 | −7.921 | −8.002 | −8.082 | −8.162 | −8.241 | −8.320 | −8.399 | −8.476 | −8.553 | −8.630 | −8.705 | −8.778 | −8.850 | −8.921 |
| 0.225 | 0.157 | 0.089 | 0.023 | −0.043 | −0.107 | −0.171 | −0.234 | −0.296 | −0.357 | −0.417 | −0.477 | −0.536 | −0.594 | −0.652 | −0.708 | −0.764 |
| | | | | | | | | | | | | | | | | |
| −7.184 | −7.274 | −7.366 | −7.458 | −7.552 | −7.646 | −7.742 | −7.837 | −7.934 | −8.031 | −8.128 | −8.226 | −8.324 | −8.421 | −8.518 | −8.615 | −8.711 |
| 0.707 | 0.654 | 0.600 | 0.546 | 0.492 | 0.437 | 0.382 | 0.326 | 0.270 | 0.214 | 0.157 | 0.100 | 0.042 | −0.016 | −0.073 | −0.131 | −0.189 |

| | | | | | | | | | | | | | | | | |
|---|---|---|---|---|---|---|---|---|---|---|---|---|---|---|---|---|
| 2.817 | 2.819 | 2.820 | 2.822 | 2.824 | 2.825 | 2.827 | 2.829 | 2.830 | 2.832 | 2.834 | 2.835 | 2.837 | 2.839 | 2.840 | 2.842 | 2.844 |
| −8.991 | −9.058 | −9.124 | −9.188 | −9.251 | −9.312 | −9.371 | −9.429 | −9.485 | −9.539 | −9.592 | −9.644 | −9.693 | −9.742 | −9.788 | −9.833 | −9.877 |
| −0.818 | −0.872 | −0.924 | −0.975 | −1.025 | −1.074 | −1.122 | −1.169 | −1.214 | −1.258 | −1.301 | −1.342 | −1.382 | −1.421 | −1.458 | −1.494 | −1.528 |
| −8.805 | −8.899 | −8.991 | −9.081 | −9.170 | −9.257 | −9.343 | −9.426 | −9.507 | −9.586 | −9.664 | −9.739 | −9.811 | −9.882 | −9.950 | −10.015 | −10.078 |
| −0.247 | −0.304 | −0.361 | −0.417 | −0.472 | −0.527 | −0.580 | −0.632 | −0.683 | −0.732 | −0.780 | −0.827 | −0.871 | −0.914 | −0.955 | −0.994 | −1.031 |

| | | | | | | | | | | | | | | | | |
|---|---|---|---|---|---|---|---|---|---|---|---|---|---|---|---|---|
| 2.845 | 2.847 | 2.849 | 2.850 | 2.852 | 2.854 | 2.855 | 2.857 | 2.859 | 2.860 | 2.862 | 2.864 | 2.865 | 2.867 | 2.869 | 2.870 | 2.872 |
| −9.919 | −9.959 | −9.999 | −10.036 | −10.072 | −10.107 | −10.140 | −10.172 | −10.201 | −10.229 | −10.256 | −10.280 | −10.302 | −10.322 | −10.339 | −10.355 | −10.368 |
| −1.561 | −1.592 | −1.621 | −1.648 | −1.673 | −1.696 | −1.716 | −1.734 | −1.750 | −1.763 | −1.774 | −1.783 | −1.790 | −1.794 | −1.796 | −1.796 | −1.793 |
| −10.138 | −10.196 | −10.250 | −10.301 | −10.349 | −10.394 | −10.436 | −10.474 | −10.509 | −10.541 | −10.570 | −10.597 | −10.621 | −10.642 | −10.661 | −10.677 | −10.690 |
| −1.066 | −1.100 | −1.131 | −1.161 | −1.188 | −1.212 | −1.234 | −1.254 | −1.271 | −1.286 | −1.298 | −1.307 | −1.313 | −1.316 | −1.316 | −1.314 | −1.309 |

| | | | | | | | | | | | | | | | | |
|---|---|---|---|---|---|---|---|---|---|---|---|---|---|---|---|---|
| 2.874 | 2.875 | 2.877 | 2.879 | 2.880 | 2.882 | 2.884 | 2.885 | 2.887 | 2.889 | 2.890 | 2.892 | 2.894 | 2.895 | 2.897 | 2.899 | 2.900 |
| −10.380 | −10.389 | −10.396 | −10.401 | −10.405 | −10.406 | −10.406 | −10.404 | −10.400 | −10.394 | −10.386 | −10.377 | −10.365 | −10.352 | −10.337 | −10.321 | −10.304 |
| −1.788 | −1.781 | −1.772 | −1.760 | −1.746 | −1.730 | −1.711 | −1.690 | −1.667 | −1.641 | −1.612 | −1.580 | −1.546 | −1.510 | −1.471 | −1.429 | −1.386 |
| −10.702 | −10.711 | −10.718 | −10.723 | −10.726 | −10.727 | −10.726 | −10.724 | −10.719 | −10.713 | −10.705 | −10.695 | −10.683 | −10.670 | −10.655 | −10.639 | −10.622 |
| −1.301 | −1.291 | −1.277 | −1.261 | −1.242 | −1.220 | −1.195 | −1.168 | −1.138 | −1.105 | −1.071 | −1.033 | −0.994 | −0.952 | −0.909 | −0.863 | −0.815 |

| | | | | | | | | | | | | | | | | |
|---|---|---|---|---|---|---|---|---|---|---|---|---|---|---|---|---|
| 2.902 | 2.904 | 2.905 | 2.907 | 2.909 | 2.910 | 2.912 | 2.914 | 2.915 | 2.917 | 2.919 | 2.920 | 2.922 | 2.924 | 2.925 | 2.927 | 2.929 |
| −10.286 | −10.266 | −10.247 | −10.226 | −10.206 | −10.184 | −10.163 | −10.142 | −10.121 | −10.100 | −10.080 | −10.060 | −10.041 | −10.023 | −10.005 | −9.987 | −9.970 |
| −1.340 | −1.291 | −1.240 | −1.187 | −1.132 | −1.074 | −1.014 | −0.953 | −0.890 | −0.825 | −0.759 | −0.692 | −0.624 | −0.555 | −0.486 | −0.416 | −0.346 |
| −10.603 | −10.584 | −10.563 | −10.543 | −10.522 | −10.501 | −10.479 | −10.458 | −10.436 | −10.415 | −10.394 | −10.374 | −10.354 | −10.335 | −10.316 | −10.299 | −10.282 |
| −0.766 | −0.715 | −0.661 | −0.607 | −0.550 | −0.492 | −0.433 | −0.372 | −0.310 | −0.246 | −0.181 | −0.115 | −0.049 | 0.019 | 0.087 | 0.156 | 0.225 |

| | | | | | | | | | | | | | | | | |
|---|---|---|---|---|---|---|---|---|---|---|---|---|---|---|---|---|
| 2.930 | 2.932 | 2.934 | 2.935 | 2.937 | 2.939 | 2.940 | 2.942 | 2.944 | 2.945 | 2.947 | 2.949 | 2.950 | 2.952 | 2.954 | 2.955 | 2.957 |
| −9.954 | −9.938 | −9.922 | −9.906 | −9.890 | −9.875 | −9.860 | −9.844 | −9.829 | −9.814 | −9.799 | −9.783 | −9.768 | −9.752 | −9.736 | −9.719 | −9.703 |
| −0.276 | −0.205 | −0.135 | −0.064 | 0.007 | 0.078 | 0.149 | 0.220 | 0.291 | 0.362 | 0.433 | 0.503 | 0.573 | 0.643 | 0.712 | 0.780 | 0.848 |
| −10.265 | −10.250 | −10.235 | −10.221 | −10.208 | −10.196 | −10.185 | −10.175 | −10.166 | −10.158 | −10.151 | −10.146 | −10.141 | −10.138 | −10.136 | −10.135 | −10.135 |
| 0.295 | 0.364 | 0.433 | 0.503 | 0.572 | 0.640 | 0.708 | 0.775 | 0.842 | 0.907 | 0.972 | 1.036 | 1.098 | 1.160 | 1.221 | 1.280 | 1.339 |

| | | | | | | | | | | | | | | | | |
|---|---|---|---|---|---|---|---|---|---|---|---|---|---|---|---|---|
| 2.959 | 2.960 | 2.962 | 2.964 | 2.965 | 2.967 | 2.969 | 2.970 | 2.972 | 2.974 | 2.975 | 2.977 | 2.979 | 2.980 | 2.982 | 2.984 | 2.985 |
| | | | | | | | | | | | | | | | | |
| −9.686 | −9.669 | −9.651 | −9.633 | −9.614 | −9.594 | −9.574 | −9.553 | −9.532 | −9.510 | −9.488 | −9.465 | −9.442 | −9.418 | −9.394 | −9.370 | −9.345 |
| 0.914 | 0.980 | 1.045 | 1.109 | 1.171 | 1.233 | 1.294 | 1.353 | 1.411 | 1.468 | 1.524 | 1.579 | 1.632 | 1.684 | 1.735 | 1.785 | 1.833 |
| | | | | | | | | | | | | | | | | |
| −10.136 | −10.138 | −10.141 | −10.144 | −10.149 | −10.154 | −10.160 | −10.166 | −10.174 | −10.182 | −10.190 | −10.200 | −10.209 | −10.219 | −10.228 | −10.238 | −10.247 |
| 1.396 | 1.451 | 1.506 | 1.558 | 1.610 | 1.660 | 1.709 | 1.756 | 1.802 | 1.846 | 1.888 | 1.929 | 1.967 | 2.004 | 2.038 | 2.070 | 2.100 |

| | | | | | | | | | | | | | | | | |
|---|---|---|---|---|---|---|---|---|---|---|---|---|---|---|---|---|
| 2.987 | 2.989 | 2.990 | 2.992 | 2.994 | 2.995 | 2.997 | 2.999 | 3.000 | 3.002 | 3.004 | 3.005 | 3.007 | 3.009 | 3.010 | 3.012 | 3.014 |
| | | | | | | | | | | | | | | | | |
| −9.320 | −9.294 | −9.268 | −9.242 | −9.214 | −9.187 | −9.159 | −9.131 | −9.103 | −9.074 | −9.046 | −9.018 | −8.991 | −8.963 | −8.937 | −8.910 | −8.885 |
| 1.881 | 1.926 | 1.971 | 2.014 | 2.056 | 2.096 | 2.135 | 2.172 | 2.207 | 2.241 | 2.273 | 2.303 | 2.331 | 2.358 | 2.383 | 2.407 | 2.428 |
| | | | | | | | | | | | | | | | | |
| −10.256 | −10.264 | −10.272 | −10.279 | −10.286 | −10.292 | −10.297 | −10.301 | −10.304 | −10.306 | −10.307 | −10.307 | −10.305 | −10.303 | −10.299 | −10.295 | −10.290 |
| 2.128 | 2.154 | 2.178 | 2.201 | 2.221 | 2.239 | 2.255 | 2.269 | 2.282 | 2.292 | 2.301 | 2.307 | 2.312 | 2.315 | 2.317 | 2.317 | 2.316 |

| | | | | | | | | | | | | | | | | |
|---|---|---|---|---|---|---|---|---|---|---|---|---|---|---|---|---|
| 3.015 | 3.017 | 3.019 | 3.020 | 3.022 | 3.024 | 3.025 | 3.027 | 3.029 | 3.030 | 3.032 | 3.034 | 3.035 | 3.037 | 3.039 | 3.040 | 3.042 |
| −8.859 | −8.834 | −8.809 | −8.784 | −8.759 | −8.734 | −8.710 | −8.685 | −8.660 | −8.635 | −8.610 | −8.584 | −8.558 | −8.531 | −8.504 | −8.476 | −8.448 |
| 2.448 | 2.466 | 2.483 | 2.498 | 2.511 | 2.523 | 2.534 | 2.543 | 2.552 | 2.559 | 2.566 | 2.571 | 2.577 | 2.581 | 2.586 | 2.589 | 2.593 |
| −10.284 | −10.277 | −10.270 | −10.261 | −10.252 | −10.241 | −10.230 | −10.218 | −10.204 | −10.190 | −10.174 | −10.157 | −10.138 | −10.118 | −10.097 | −10.074 | −10.051 |
| 2.314 | 2.311 | 2.306 | 2.301 | 2.295 | 2.289 | 2.282 | 2.275 | 2.267 | 2.259 | 2.250 | 2.241 | 2.231 | 2.222 | 2.212 | 2.203 | 2.192 |

| | | | | | | | | | | | | | | | | |
|---|---|---|---|---|---|---|---|---|---|---|---|---|---|---|---|---|
| 3.044 | 3.045 | 3.047 | 3.049 | 3.050 | 3.052 | 3.054 | 3.055 | 3.057 | 3.059 | 3.060 | 3.062 | 3.064 | 3.065 | 3.067 | 3.069 | 3.070 |
| −8.418 | −8.387 | −8.356 | −8.324 | −8.291 | −8.257 | −8.223 | −8.188 | −8.152 | −8.116 | −8.079 | −8.041 | −8.003 | −7.964 | −7.925 | −7.885 | −7.845 |
| 2.596 | 2.598 | 2.600 | 2.602 | 2.603 | 2.604 | 2.604 | 2.604 | 2.604 | 2.604 | 2.604 | 2.603 | 2.603 | 2.602 | 2.601 | 2.600 | 2.599 |
| −10.025 | −9.999 | −9.972 | −9.943 | −9.913 | −9.882 | −9.850 | −9.816 | −9.781 | −9.745 | −9.707 | −9.668 | −9.628 | −9.586 | −9.544 | −9.500 | −9.455 |
| 2.182 | 2.172 | 2.161 | 2.149 | 2.138 | 2.125 | 2.113 | 2.100 | 2.086 | 2.072 | 2.058 | 2.044 | 2.030 | 2.016 | 2.002 | 1.989 | 1.976 |

| | | | | | | | | | | | | | | | | |
|---|---|---|---|---|---|---|---|---|---|---|---|---|---|---|---|---|
| 3.072 | 3.074 | 3.075 | 3.077 | 3.079 | 3.080 | 3.082 | 3.084 | 3.085 | 3.087 | 3.089 | 3.090 | 3.092 | 3.093 | 3.095 | 3.097 | 3.098 |
| | | | | | | | | | | | | | | | | |
| −7.804 | −7.763 | −7.722 | −7.680 | −7.638 | −7.596 | −7.553 | −7.510 | −7.468 | −7.426 | −7.385 | −7.345 | −7.305 | −7.267 | −7.229 | −7.192 | −7.156 |
| 2.598 | 2.597 | 2.595 | 2.594 | 2.592 | 2.591 | 2.589 | 2.588 | 2.588 | 2.588 | 2.588 | 2.588 | 2.590 | 2.591 | 2.594 | 2.597 | 2.600 |
| | | | | | | | | | | | | | | | | |
| −9.410 | −9.363 | −9.315 | −9.267 | −9.217 | −9.167 | −9.116 | −9.064 | −9.011 | −8.958 | −8.905 | −8.852 | −8.798 | −8.745 | −8.691 | −8.638 | −8.585 |
| 1.963 | 1.951 | 1.940 | 1.929 | 1.919 | 1.911 | 1.904 | 1.898 | 1.893 | 1.889 | 1.887 | 1.886 | 1.886 | 1.888 | 1.890 | 1.894 | 1.899 |

| | | | | | | | | | | | | | | | | |
|---|---|---|---|---|---|---|---|---|---|---|---|---|---|---|---|---|
| 3.100 | 3.102 | 3.103 | 3.105 | 3.107 | 3.108 | 3.110 | 3.112 | 3.113 | 3.115 | 3.117 | 3.118 | 3.120 | 3.122 | 3.123 | 3.125 | 3.127 |
| | | | | | | | | | | | | | | | | |
| −7.120 | −7.085 | −7.050 | −7.016 | −6.982 | −6.949 | −6.916 | −6.885 | −6.853 | −6.823 | −6.793 | −6.765 | −6.737 | −6.710 | −6.684 | −6.659 | −6.635 |
| 2.605 | 2.610 | 2.616 | 2.623 | 2.631 | 2.640 | 2.650 | 2.660 | 2.672 | 2.685 | 2.698 | 2.712 | 2.728 | 2.744 | 2.760 | 2.778 | 2.796 |
| | | | | | | | | | | | | | | | | |
| −8.532 | −8.480 | −8.429 | −8.378 | −8.329 | −8.280 | −8.234 | −8.189 | −8.145 | −8.103 | −8.063 | −8.025 | −7.989 | −7.954 | −7.921 | −7.890 | −7.861 |
| 1.905 | 1.912 | 1.920 | 1.928 | 1.937 | 1.947 | 1.957 | 1.966 | 1.977 | 1.987 | 1.998 | 2.008 | 2.019 | 2.030 | 2.042 | 2.054 | 2.066 |

| | | | | | | | | | | | | | | | | |
|---|---|---|---|---|---|---|---|---|---|---|---|---|---|---|---|---|
| 3.128 | 3.130 | 3.132 | 3.133 | 3.135 | 3.137 | 3.138 | 3.140 | 3.142 | 3.143 | 3.145 | 3.147 | 3.148 | 3.150 | 3.152 | 3.153 | 3.155 |
| −6.612 | −6.591 | −6.571 | −6.554 | −6.537 | −6.523 | −6.510 | −6.499 | −6.489 | −6.480 | −6.472 | −6.465 | −6.459 | −6.454 | −6.449 | −6.445 | −6.441 |
| 2.816 | 2.836 | 2.856 | 2.877 | 2.899 | 2.921 | 2.943 | 2.965 | 2.988 | 3.011 | 3.034 | 3.056 | 3.079 | 3.102 | 3.124 | 3.146 | 3.168 |
| −7.833 | −7.808 | −7.785 | −7.764 | −7.745 | −7.728 | −7.712 | −7.699 | −7.688 | −7.679 | −7.671 | −7.666 | −7.663 | −7.661 | −7.661 | −7.663 | −7.667 |
| 2.079 | 2.091 | 2.104 | 2.117 | 2.129 | 2.142 | 2.154 | 2.167 | 2.178 | 2.190 | 2.201 | 2.212 | 2.222 | 2.232 | 2.242 | 2.251 | 2.259 |

| | | | | | | | | | | | | | | | | |
|---|---|---|---|---|---|---|---|---|---|---|---|---|---|---|---|---|
| 3.157 | 3.158 | 3.160 | 3.162 | 3.163 | 3.165 | 3.167 | 3.168 | 3.170 | 3.172 | 3.173 | 3.175 | 3.177 | 3.178 | 3.180 | 3.182 | 3.183 |
| −6.437 | −6.434 | −6.431 | −6.429 | −6.426 | −6.424 | −6.422 | −6.419 | −6.417 | −6.415 | −6.414 | −6.412 | −6.410 | −6.409 | −6.407 | −6.406 | −6.405 |
| 3.189 | 3.211 | 3.232 | 3.253 | 3.274 | 3.294 | 3.314 | 3.334 | 3.354 | 3.373 | 3.392 | 3.411 | 3.429 | 3.447 | 3.464 | 3.482 | 3.499 |
| −7.672 | −7.678 | −7.686 | −7.695 | −7.706 | −7.719 | −7.733 | −7.748 | −7.765 | −7.783 | −7.802 | −7.822 | −7.843 | −7.865 | −7.887 | −7.909 | −7.932 |
| 2.268 | 2.276 | 2.283 | 2.290 | 2.297 | 2.303 | 2.308 | 2.313 | 2.317 | 2.321 | 2.325 | 2.328 | 2.330 | 2.332 | 2.332 | 2.333 | 2.332 |

| | | | | | | | | | | | | | | | | |
|---|---|---|---|---|---|---|---|---|---|---|---|---|---|---|---|---|
| 3.185 | 3.187 | 3.188 | 3.190 | 3.192 | 3.193 | 3.195 | 3.197 | 3.198 | 3.200 | 3.202 | 3.203 | 3.205 | 3.207 | 3.208 | 3.210 | 3.212 |
| −6.404 | −6.402 | −6.401 | −6.399 | −6.397 | −6.394 | −6.392 | −6.388 | −6.384 | −6.379 | −6.374 | −6.368 | −6.361 | −6.353 | −6.344 | −6.334 | −6.323 |
| 3.516 | 3.533 | 3.550 | 3.566 | 3.583 | 3.600 | 3.617 | 3.634 | 3.651 | 3.668 | 3.685 | 3.701 | 3.718 | 3.734 | 3.749 | 3.765 | 3.780 |
| −7.954 | −7.976 | −7.998 | −8.019 | −8.040 | −8.060 | −8.078 | −8.095 | −8.111 | −8.125 | −8.137 | −8.148 | −8.156 | −8.163 | −8.168 | −8.172 | −8.173 |
| 2.331 | 2.330 | 2.327 | 2.324 | 2.321 | 2.317 | 2.313 | 2.308 | 2.303 | 2.298 | 2.292 | 2.286 | 2.279 | 2.271 | 2.264 | 2.256 | 2.248 |

| | | | | | | | | | | | | | | | | |
|---|---|---|---|---|---|---|---|---|---|---|---|---|---|---|---|---|
| 3.213 | 3.215 | 3.217 | 3.218 | 3.220 | 3.222 | 3.223 | 3.225 | 3.227 | 3.228 | 3.230 | 3.232 | 3.233 | 3.235 | 3.237 | 3.238 | 3.240 |
| −6.310 | −6.297 | −6.282 | −6.267 | −6.250 | −6.232 | −6.213 | −6.193 | −6.172 | −6.150 | −6.128 | −6.104 | −6.080 | −6.054 | −6.028 | −6.000 | −5.972 |
| 3.794 | 3.809 | 3.823 | 3.837 | 3.850 | 3.864 | 3.878 | 3.892 | 3.906 | 3.920 | 3.934 | 3.949 | 3.963 | 3.977 | 3.991 | 4.005 | 4.018 |
| −8.173 | −8.171 | −8.166 | −8.160 | −8.152 | −8.142 | −8.129 | −8.114 | −8.097 | −8.078 | −8.055 | −8.031 | −8.003 | −7.973 | −7.940 | −7.904 | −7.866 |
| 2.239 | 2.231 | 2.223 | 2.214 | 2.206 | 2.199 | 2.192 | 2.186 | 2.181 | 2.177 | 2.174 | 2.172 | 2.172 | 2.172 | 2.173 | 2.176 | 2.179 |

| | | | | | | | | | | | | | | | | |
|---|---|---|---|---|---|---|---|---|---|---|---|---|---|---|---|---|
| 3.242 | 3.243 | 3.245 | 3.247 | 3.248 | 3.250 | 3.252 | 3.253 | 3.255 | 3.257 | 3.258 | 3.260 | 3.262 | 3.263 | 3.265 | 3.267 | 3.268 |
| −5.944 | −5.915 | −5.886 | −5.857 | −5.828 | −5.800 | −5.771 | −5.742 | −5.714 | −5.686 | −5.658 | −5.629 | −5.601 | −5.572 | −5.544 | −5.516 | −5.487 |
| 4.031 | 4.044 | 4.057 | 4.070 | 4.083 | 4.095 | 4.107 | 4.119 | 4.130 | 4.141 | 4.152 | 4.162 | 4.173 | 4.183 | 4.194 | 4.204 | 4.214 |
| −7.825 | −7.783 | −7.738 | −7.691 | −7.642 | −7.591 | −7.538 | −7.483 | −7.426 | −7.367 | −7.307 | −7.245 | −7.181 | −7.116 | −7.049 | −6.981 | −6.912 |
| 2.183 | 2.189 | 2.196 | 2.203 | 2.212 | 2.222 | 2.234 | 2.246 | 2.259 | 2.274 | 2.289 | 2.306 | 2.325 | 2.344 | 2.365 | 2.387 | 2.410 |

| | | | | | | | | | | | | | | | | |
|---|---|---|---|---|---|---|---|---|---|---|---|---|---|---|---|---|
| 3.270 | 3.272 | 3.273 | 3.275 | 3.277 | 3.278 | 3.280 | 3.282 | 3.283 | 3.285 | 3.287 | 3.288 | 3.290 | 3.292 | 3.293 | 3.295 | 3.297 |
| | | | | | | | | | | | | | | | | |
| −5.459 | −5.430 | −5.401 | −5.372 | −5.343 | −5.313 | −5.284 | −5.254 | −5.224 | −5.193 | −5.163 | −5.132 | −5.102 | −5.072 | −5.041 | −5.011 | −4.981 |
| 4.225 | 4.235 | 4.244 | 4.254 | 4.263 | 4.272 | 4.281 | 4.290 | 4.298 | 4.307 | 4.315 | 4.323 | 4.330 | 4.338 | 4.345 | 4.351 | 4.358 |
| | | | | | | | | | | | | | | | | |
| −6.842 | −6.772 | −6.702 | −6.632 | −6.562 | −6.493 | −6.425 | −6.357 | −6.291 | −6.226 | −6.162 | −6.100 | −6.039 | −5.980 | −5.922 | −5.866 | −5.811 |
| 2.433 | 2.457 | 2.482 | 2.507 | 2.532 | 2.558 | 2.585 | 2.612 | 2.639 | 2.667 | 2.695 | 2.724 | 2.752 | 2.781 | 2.811 | 2.840 | 2.869 |

| | | | | | | | | | | | | | | | | |
|---|---|---|---|---|---|---|---|---|---|---|---|---|---|---|---|---|
| 3.298 | 3.300 | 3.302 | 3.303 | 3.305 | 3.307 | 3.308 | 3.310 | 3.312 | 3.313 | 3.315 | 3.317 | 3.318 | 3.320 | 3.322 | 3.323 | 3.325 |
| −4.950 | −4.919 | −4.889 | −4.858 | −4.827 | −4.797 | −4.767 | −4.738 | −4.709 | −4.681 | −4.653 | −4.626 | −4.599 | −4.574 | −4.549 | −4.525 | −4.501 |
| 4.363 | 4.368 | 4.372 | 4.376 | 4.378 | 4.380 | 4.381 | 4.381 | 4.379 | 4.376 | 4.371 | 4.364 | 4.356 | 4.347 | 4.335 | 4.322 | 4.307 |
| −5.757 | −5.705 | −5.655 | −5.606 | −5.559 | −5.513 | −5.469 | −5.427 | −5.387 | −5.348 | −5.311 | −5.277 | −5.244 | −5.213 | −5.184 | −5.158 | −5.133 |
| 2.897 | 2.925 | 2.953 | 2.980 | 3.006 | 3.031 | 3.054 | 3.077 | 3.098 | 3.118 | 3.136 | 3.153 | 3.168 | 3.181 | 3.193 | 3.203 | 3.210 |

| | | | | | | | | | | | | | | | | |
|---|---|---|---|---|---|---|---|---|---|---|---|---|---|---|---|---|
| 3.327 | 3.328 | 3.330 | 3.332 | 3.333 | 3.335 | 3.337 | 3.338 | 3.340 | 3.342 | 3.343 | 3.345 | 3.347 | 3.348 | 3.350 | 3.352 | 3.353 |
| | | | | | | | | | | | | | | | | |
| −4.478 | −4.456 | −4.435 | −4.415 | −4.397 | −4.380 | −4.364 | −4.350 | −4.338 | −4.327 | −4.319 | −4.312 | −4.307 | −4.305 | −4.304 | −4.306 | −4.311 |
| 4.291 | 4.272 | 4.252 | 4.229 | 4.205 | 4.178 | 4.149 | 4.117 | 4.083 | 4.047 | 4.007 | 3.966 | 3.921 | 3.874 | 3.824 | 3.771 | 3.716 |
| | | | | | | | | | | | | | | | | |
| −5.110 | −5.089 | −5.070 | −5.054 | −5.040 | −5.028 | −5.019 | −5.012 | −5.009 | −5.008 | −5.010 | −5.016 | −5.024 | −5.035 | −5.049 | −5.066 | −5.086 |
| 3.216 | 3.218 | 3.219 | 3.217 | 3.212 | 3.205 | 3.194 | 3.181 | 3.164 | 3.145 | 3.122 | 3.096 | 3.067 | 3.034 | 2.998 | 2.959 | 2.917 |

| | | | | | | | | | | | | | | | | |
|---|---|---|---|---|---|---|---|---|---|---|---|---|---|---|---|---|
| 3.355 | 3.357 | 3.358 | 3.360 | 3.362 | 3.363 | 3.365 | 3.367 | 3.368 | 3.370 | 3.372 | 3.373 | 3.375 | 3.377 | 3.378 | 3.380 | 3.382 |
| −4.318 | −4.328 | −4.341 | −4.357 | −4.375 | −4.397 | −4.422 | −4.449 | −4.479 | −4.512 | −4.548 | −4.586 | −4.627 | −4.670 | −4.716 | −4.763 | −4.813 |
| 3.659 | 3.598 | 3.535 | 3.469 | 3.400 | 3.329 | 3.254 | 3.178 | 3.098 | 3.016 | 2.932 | 2.845 | 2.756 | 2.664 | 2.571 | 2.476 | 2.380 |
| −5.108 | −5.133 | −5.161 | −5.191 | −5.223 | −5.257 | −5.294 | −5.332 | −5.373 | −5.417 | −5.463 | −5.511 | −5.562 | −5.615 | −5.670 | −5.728 | −5.788 |
| 2.872 | 2.824 | 2.773 | 2.719 | 2.663 | 2.603 | 2.542 | 2.477 | 2.410 | 2.340 | 2.267 | 2.192 | 2.114 | 2.033 | 1.950 | 1.865 | 1.778 |

| | | | | | | | | | | | | | | | | |
|---|---|---|---|---|---|---|---|---|---|---|---|---|---|---|---|---|
| 3.383 | 3.385 | 3.387 | 3.388 | 3.390 | 3.392 | 3.393 | 3.395 | 3.397 | 3.398 | 3.400 | 3.402 | 3.403 | 3.405 | 3.407 | 3.408 | 3.410 |
| −4.865 | −4.918 | −4.973 | −5.029 | −5.086 | −5.145 | −5.206 | −5.268 | −5.331 | −5.396 | −5.461 | −5.528 | −5.596 | −5.664 | −5.733 | −5.803 | −5.874 |
| 2.281 | 2.182 | 2.080 | 1.978 | 1.874 | 1.768 | 1.662 | 1.555 | 1.447 | 1.338 | 1.229 | 1.119 | 1.009 | 0.899 | 0.789 | 0.679 | 0.569 |
| −5.849 | −5.913 | −5.978 | −6.044 | −6.113 | −6.182 | −6.253 | −6.326 | −6.399 | −6.474 | −6.550 | −6.628 | −6.707 | −6.787 | −6.868 | −6.950 | −7.034 |
| 1.689 | 1.598 | 1.506 | 1.411 | 1.315 | 1.218 | 1.119 | 1.019 | 0.919 | 0.818 | 0.717 | 0.615 | 0.513 | 0.412 | 0.310 | 0.209 | 0.108 |

| | | | | | | | | | | | | | | | | |
|---|---|---|---|---|---|---|---|---|---|---|---|---|---|---|---|---|
| 3.412 | 3.413 | 3.415 | 3.416 | 3.418 | 3.420 | 3.421 | 3.423 | 3.425 | 3.426 | 3.428 | 3.430 | 3.431 | 3.433 | 3.435 | 3.436 | 3.438 |
| | | | | | | | | | | | | | | | | |
| −5.944 | −6.015 | −6.087 | −6.158 | −6.230 | −6.301 | −6.372 | −6.443 | −6.513 | −6.582 | −6.651 | −6.720 | −6.788 | −6.856 | −6.924 | −6.991 | −7.058 |
| 0.460 | 0.351 | 0.243 | 0.135 | 0.028 | −0.077 | −0.182 | −0.286 | −0.388 | −0.489 | −0.588 | −0.686 | −0.783 | −0.878 | −0.971 | −1.062 | −1.152 |
| | | | | | | | | | | | | | | | | |
| −7.118 | −7.204 | −7.290 | −7.378 | −7.466 | −7.554 | −7.644 | −7.734 | −7.824 | −7.915 | −8.006 | −8.097 | −8.188 | −8.278 | −8.368 | −8.458 | −8.548 |
| 0.007 | −0.093 | −0.192 | −0.291 | −0.389 | −0.486 | −0.582 | −0.677 | −0.771 | −0.864 | −0.956 | −1.046 | −1.135 | −1.222 | −1.308 | −1.392 | −1.475 |

| | | | | | | | | | | | | | | | | |
|---|---|---|---|---|---|---|---|---|---|---|---|---|---|---|---|---|
| 3.440 | 3.441 | 3.443 | 3.445 | 3.446 | 3.448 | 3.450 | 3.451 | 3.453 | 3.455 | 3.456 | 3.458 | 3.460 | 3.461 | 3.463 | 3.465 | 3.466 |
| −7.125 | −7.192 | −7.258 | −7.324 | −7.391 | −7.457 | −7.524 | −7.590 | −7.657 | −7.724 | −7.791 | −7.859 | −7.927 | −7.996 | −8.064 | −8.133 | −8.203 |
| −1.240 | −1.327 | −1.412 | −1.495 | −1.577 | −1.657 | −1.735 | −1.811 | −1.885 | −1.957 | −2.028 | −2.096 | −2.161 | −2.225 | −2.285 | −2.344 | −2.399 |
| −8.637 | −8.726 | −8.814 | −8.901 | −8.988 | −9.074 | −9.159 | −9.244 | −9.327 | −9.409 | −9.491 | −9.571 | −9.650 | −9.729 | −9.806 | −9.883 | −9.959 |
| −1.556 | −1.636 | −1.714 | −1.790 | −1.865 | −1.938 | −2.009 | −2.079 | −2.146 | −2.212 | −2.276 | −2.338 | −2.398 | −2.456 | −2.512 | −2.566 | −2.618 |

| | | | | | | | | | | | | | | | | |
|---|---|---|---|---|---|---|---|---|---|---|---|---|---|---|---|---|
| 3.468 | 3.470 | 3.471 | 3.473 | 3.475 | 3.476 | 3.478 | 3.480 | 3.481 | 3.483 | 3.485 | 3.486 | 3.488 | 3.490 | 3.491 | 3.493 | 3.495 |
| −8.273 | −8.343 | −8.413 | −8.483 | −8.553 | −8.622 | −8.691 | −8.759 | −8.826 | −8.892 | −8.957 | −9.021 | −9.084 | −9.147 | −9.208 | −9.268 | −9.328 |
| −2.451 | −2.501 | −2.547 | −2.590 | −2.629 | −2.666 | −2.699 | −2.728 | −2.754 | −2.777 | −2.796 | −2.812 | −2.825 | −2.835 | −2.842 | −2.846 | −2.847 |
| −10.034 | −10.108 | −10.181 | −10.252 | −10.323 | −10.392 | −10.460 | −10.527 | −10.593 | −10.657 | −10.721 | −10.784 | −10.845 | −10.906 | −10.966 | −11.026 | −11.084 |
| −2.668 | −2.716 | −2.761 | −2.805 | −2.846 | −2.885 | −2.922 | −2.956 | −2.987 | −3.016 | −3.041 | −3.064 | −3.083 | −3.100 | −3.114 | −3.125 | −3.134 |

| | | | | | | | | | | | | | | | | |
|---|---|---|---|---|---|---|---|---|---|---|---|---|---|---|---|---|
| 3.496 | 3.498 | 3.500 | 3.501 | 3.503 | 3.505 | 3.506 | 3.508 | 3.510 | 3.511 | 3.513 | 3.515 | 3.516 | 3.518 | 3.520 | 3.521 | 3.523 |
| −9.386 | −9.443 | −9.498 | −9.553 | −9.606 | −9.657 | −9.706 | −9.753 | −9.799 | −9.843 | −9.885 | −9.925 | −9.962 | −9.998 | −10.031 | −10.063 | −10.092 |
| −2.846 | −2.841 | −2.834 | −2.825 | −2.812 | −2.797 | −2.780 | −2.759 | −2.736 | −2.711 | −2.684 | −2.654 | −2.622 | −2.589 | −2.553 | −2.515 | −2.476 |
| −11.142 | −11.199 | −11.255 | −11.310 | −11.365 | −11.419 | −11.471 | −11.523 | −11.574 | −11.624 | −11.673 | −11.720 | −11.767 | −11.812 | −11.856 | −11.898 | −11.938 |
| −3.139 | −3.142 | −3.142 | −3.139 | −3.133 | −3.124 | −3.112 | −3.098 | −3.080 | −3.060 | −3.037 | −3.012 | −2.983 | −2.952 | −2.918 | −2.882 | −2.843 |

| | | | | | | | | | | | | | | | | |
|---|---|---|---|---|---|---|---|---|---|---|---|---|---|---|---|---|
| 3.525 | 3.526 | 3.528 | 3.530 | 3.531 | 3.533 | 3.535 | 3.536 | 3.538 | 3.540 | 3.541 | 3.543 | 3.545 | 3.546 | 3.548 | 3.550 | 3.551 |
| −10.118 | −10.142 | −10.165 | −10.185 | −10.203 | −10.218 | −10.232 | −10.243 | −10.253 | −10.260 | −10.266 | −10.270 | −10.273 | −10.275 | −10.275 | −10.275 | −10.273 |
| −2.434 | −2.391 | −2.346 | −2.300 | −2.251 | −2.201 | −2.150 | −2.098 | −2.045 | −1.991 | −1.936 | −1.881 | −1.825 | −1.768 | −1.711 | −1.654 | −1.597 |
| −11.977 | −12.015 | −12.051 | −12.086 | −12.118 | −12.150 | −12.179 | −12.207 | −12.234 | −12.258 | −12.281 | −12.303 | −12.323 | −12.341 | −12.359 | −12.374 | −12.389 |
| −2.801 | −2.757 | −2.711 | −2.663 | −2.613 | −2.561 | −2.508 | −2.453 | −2.397 | −2.339 | −2.281 | −2.221 | −2.160 | −2.098 | −2.036 | −1.973 | −1.909 |

| | | | | | | | | | | | | | | | | |
|---|---|---|---|---|---|---|---|---|---|---|---|---|---|---|---|---|
| 3.553 | 3.555 | 3.556 | 3.558 | 3.560 | 3.561 | 3.563 | 3.565 | 3.566 | 3.568 | 3.570 | 3.571 | 3.573 | 3.575 | 3.576 | 3.578 | 3.580 |
| −10.271 | −10.268 | −10.264 | −10.260 | −10.256 | −10.251 | −10.245 | −10.239 | −10.233 | −10.227 | −10.220 | −10.213 | −10.205 | −10.197 | −10.189 | −10.181 | −10.172 |
| −1.539 | −1.481 | −1.424 | −1.367 | −1.310 | −1.253 | −1.196 | −1.140 | −1.083 | −1.027 | −0.971 | −0.916 | −0.860 | −0.805 | −0.750 | −0.695 | −0.640 |
| −12.402 | −12.414 | −12.425 | −12.434 | −12.443 | −12.451 | −12.457 | −12.463 | −12.469 | −12.473 | −12.478 | −12.481 | −12.485 | −12.488 | −12.491 | −12.493 | −12.495 |
| −1.844 | −1.779 | −1.713 | −1.647 | −1.581 | −1.515 | −1.448 | −1.382 | −1.316 | −1.249 | −1.183 | −1.117 | −1.052 | −0.986 | −0.921 | −0.857 | −0.793 |

| | | | | | | | | | | | | | | | | |
|---|---|---|---|---|---|---|---|---|---|---|---|---|---|---|---|---|
| 3.581 | 3.583 | 3.585 | 3.586 | 3.588 | 3.590 | 3.591 | 3.593 | 3.595 | 3.596 | 3.598 | 3.600 | 3.601 | 3.603 | 3.605 | 3.606 | 3.608 |
| −10.164 | −10.155 | −10.146 | −10.138 | −10.129 | −10.121 | −10.113 | −10.106 | −10.099 | −10.093 | −10.088 | −10.082 | −10.078 | −10.075 | −10.072 | −10.071 | −10.071 |
| −0.586 | −0.533 | −0.480 | −0.428 | −0.376 | −0.326 | −0.277 | −0.228 | −0.181 | −0.135 | −0.090 | −0.046 | −0.003 | 0.039 | 0.079 | 0.119 | 0.158 |
| −12.497 | −12.497 | −12.498 | −12.498 | −12.498 | −12.497 | −12.495 | −12.492 | −12.490 | −12.486 | −12.482 | −12.477 | −12.471 | −12.464 | −12.456 | −12.448 | −12.438 |
| −0.730 | −0.668 | −0.607 | −0.547 | −0.489 | −0.431 | −0.375 | −0.320 | −0.266 | −0.214 | −0.163 | −0.113 | −0.065 | −0.018 | 0.028 | 0.072 | 0.115 |

| | | | | | | | | | | | | | | | | |
|---|---|---|---|---|---|---|---|---|---|---|---|---|---|---|---|---|
| 3.610 | 3.611 | 3.613 | 3.615 | 3.616 | 3.618 | 3.620 | 3.621 | 3.623 | 3.625 | 3.626 | 3.628 | 3.630 | 3.631 | 3.633 | 3.635 | 3.636 |
| −10.072 | −10.073 | −10.075 | −10.078 | −10.082 | −10.085 | −10.090 | −10.094 | −10.098 | −10.103 | −10.107 | −10.110 | −10.113 | −10.115 | −10.117 | −10.117 | −10.117 |
| 0.196 | 0.233 | 0.270 | 0.305 | 0.340 | 0.374 | 0.407 | 0.439 | 0.471 | 0.502 | 0.531 | 0.560 | 0.588 | 0.614 | 0.640 | 0.664 | 0.688 |
| −12.428 | −12.417 | −12.405 | −12.393 | −12.380 | −12.367 | −12.353 | −12.338 | −12.323 | −12.307 | −12.291 | −12.274 | −12.256 | −12.238 | −12.220 | −12.202 | −12.183 |
| 0.156 | 0.196 | 0.236 | 0.273 | 0.310 | 0.345 | 0.378 | 0.410 | 0.441 | 0.470 | 0.498 | 0.524 | 0.549 | 0.573 | 0.595 | 0.616 | 0.636 |

| | | | | | | | | | | | | | | | | |
|---|---|---|---|---|---|---|---|---|---|---|---|---|---|---|---|---|
| 3.638 | 3.640 | 3.641 | 3.643 | 3.645 | 3.646 | 3.648 | 3.650 | 3.651 | 3.653 | 3.655 | 3.656 | 3.658 | 3.660 | 3.661 | 3.663 | 3.665 |
| −10.116 | −10.114 | −10.111 | −10.108 | −10.103 | −10.097 | −10.089 | −10.080 | −10.069 | −10.057 | −10.043 | −10.028 | −10.010 | −9.991 | −9.970 | −9.947 | −9.921 |
| 0.711 | 0.732 | 0.754 | 0.774 | 0.794 | 0.813 | 0.833 | 0.851 | 0.869 | 0.887 | 0.904 | 0.921 | 0.938 | 0.954 | 0.971 | 0.987 | 1.003 |
| −12.164 | −12.145 | −12.126 | −12.106 | −12.086 | −12.066 | −12.046 | −12.026 | −12.005 | −11.984 | −11.962 | −11.940 | −11.918 | −11.895 | −11.871 | −11.847 | −11.823 |
| 0.655 | 0.673 | 0.690 | 0.706 | 0.722 | 0.737 | 0.751 | 0.765 | 0.778 | 0.791 | 0.804 | 0.815 | 0.827 | 0.838 | 0.849 | 0.860 | 0.870 |

| | | | | | | | | | | | | | | | | |
|---|---|---|---|---|---|---|---|---|---|---|---|---|---|---|---|---|
| 3.666 | 3.668 | 3.670 | 3.671 | 3.673 | 3.675 | 3.676 | 3.678 | 3.680 | 3.681 | 3.683 | 3.685 | 3.686 | 3.688 | 3.690 | 3.691 | 3.693 |
| −9.894 | −9.865 | −9.833 | −9.798 | −9.762 | −9.723 | −9.682 | −9.638 | −9.593 | −9.547 | −9.499 | −9.451 | −9.401 | −9.351 | −9.301 | −9.250 | −9.199 |
| 1.019 | 1.036 | 1.051 | 1.067 | 1.083 | 1.099 | 1.114 | 1.130 | 1.145 | 1.161 | 1.176 | 1.190 | 1.205 | 1.219 | 1.233 | 1.248 | 1.262 |
| −11.799 | −11.773 | −11.748 | −11.721 | −11.694 | −11.666 | −11.637 | −11.608 | −11.578 | −11.547 | −11.516 | −11.485 | −11.452 | −11.418 | −11.384 | −11.349 | −11.313 |
| 0.881 | 0.891 | 0.901 | 0.912 | 0.922 | 0.933 | 0.944 | 0.955 | 0.966 | 0.977 | 0.989 | 0.999 | 1.010 | 1.021 | 1.031 | 1.042 | 1.052 |

| | | | | | | | | | | | | | | | | |
|---|---|---|---|---|---|---|---|---|---|---|---|---|---|---|---|---|
| 3.695 | 3.696 | 3.698 | 3.700 | 3.701 | 3.703 | 3.705 | 3.706 | 3.708 | 3.710 | 3.711 | 3.713 | 3.715 | 3.716 | 3.718 | 3.720 | 3.721 |
| −9.149 | −9.098 | −9.048 | −8.998 | −8.948 | −8.900 | −8.852 | −8.805 | −8.759 | −8.714 | −8.671 | −8.628 | −8.586 | −8.545 | −8.506 | −8.467 | −8.430 |
| 1.276 | 1.290 | 1.304 | 1.318 | 1.332 | 1.346 | 1.360 | 1.374 | 1.388 | 1.402 | 1.416 | 1.431 | 1.446 | 1.461 | 1.476 | 1.492 | 1.508 |
| −11.276 | −11.238 | −11.200 | −11.162 | −11.123 | −11.084 | −11.045 | −11.006 | −10.966 | −10.926 | −10.887 | −10.847 | −10.807 | −10.767 | −10.727 | −10.687 | −10.647 |
| 1.063 | 1.074 | 1.084 | 1.095 | 1.105 | 1.116 | 1.127 | 1.138 | 1.149 | 1.161 | 1.173 | 1.185 | 1.198 | 1.211 | 1.225 | 1.238 | 1.252 |

| | | | | | | | | | | | | | | | | |
|---|---|---|---|---|---|---|---|---|---|---|---|---|---|---|---|---|
| 3.723 | 3.725 | 3.726 | 3.728 | 3.730 | 3.731 | 3.733 | 3.735 | 3.736 | 3.738 | 3.740 | 3.741 | 3.743 | 3.744 | 3.746 | 3.748 | 3.749 |
| −8.393 | −8.358 | −8.323 | −8.289 | −8.256 | −8.223 | −8.191 | −8.160 | −8.129 | −8.099 | −8.069 | −8.040 | −8.012 | −7.983 | −7.955 | −7.927 | −7.899 |
| 1.524 | 1.540 | 1.557 | 1.574 | 1.590 | 1.607 | 1.623 | 1.640 | 1.656 | 1.673 | 1.690 | 1.707 | 1.724 | 1.742 | 1.759 | 1.777 | 1.795 |
| −10.607 | −10.567 | −10.527 | −10.487 | −10.447 | −10.406 | −10.365 | −10.324 | −10.282 | −10.239 | −10.195 | −10.150 | −10.105 | −10.058 | −10.011 | −9.963 | −9.915 |
| 1.267 | 1.281 | 1.296 | 1.311 | 1.326 | 1.341 | 1.356 | 1.371 | 1.386 | 1.402 | 1.417 | 1.432 | 1.448 | 1.464 | 1.479 | 1.495 | 1.511 |

| | | | | | | | | | | | | | | | | |
|---|---|---|---|---|---|---|---|---|---|---|---|---|---|---|---|---|
| 3.751 | 3.753 | 3.754 | 3.756 | 3.758 | 3.759 | 3.761 | 3.763 | 3.764 | 3.766 | 3.768 | 3.769 | 3.771 | 3.773 | 3.774 | 3.776 | 3.778 |
| −7.870 | −7.843 | −7.815 | −7.787 | −7.760 | −7.732 | −7.704 | −7.677 | −7.650 | −7.623 | −7.596 | −7.569 | −7.543 | −7.517 | −7.491 | −7.466 | −7.442 |
| 1.813 | 1.831 | 1.849 | 1.868 | 1.886 | 1.904 | 1.923 | 1.941 | 1.960 | 1.978 | 1.997 | 2.015 | 2.033 | 2.050 | 2.067 | 2.084 | 2.100 |
| −9.865 | −9.814 | −9.763 | −9.711 | −9.658 | −9.604 | −9.550 | −9.496 | −9.441 | −9.386 | −9.331 | −9.275 | −9.220 | −9.164 | −9.109 | −9.054 | −9.000 |
| 1.528 | 1.544 | 1.560 | 1.577 | 1.593 | 1.609 | 1.625 | 1.641 | 1.656 | 1.672 | 1.687 | 1.703 | 1.718 | 1.734 | 1.748 | 1.763 | 1.777 |

| | | | | | | | | | | | | | | | | |
|---|---|---|---|---|---|---|---|---|---|---|---|---|---|---|---|---|
| 3.779 | 3.781 | 3.783 | 3.784 | 3.786 | 3.788 | 3.789 | 3.791 | 3.793 | 3.794 | 3.796 | 3.798 | 3.799 | 3.801 | 3.803 | 3.804 | 3.806 |
| | | | | | | | | | | | | | | | | |
| −7.418 | −7.395 | −7.373 | −7.351 | −7.329 | −7.308 | −7.287 | −7.266 | −7.245 | −7.225 | −7.204 | −7.183 | −7.162 | −7.141 | −7.120 | −7.099 | −7.077 |
| 2.115 | 2.130 | 2.145 | 2.158 | 2.171 | 2.183 | 2.194 | 2.203 | 2.212 | 2.220 | 2.227 | 2.233 | 2.238 | 2.242 | 2.245 | 2.248 | 2.249 |
| | | | | | | | | | | | | | | | | |
| −8.946 | −8.893 | −8.841 | −8.790 | −8.739 | −8.690 | −8.642 | −8.595 | −8.550 | −8.507 | −8.465 | −8.424 | −8.386 | −8.349 | −8.315 | −8.282 | −8.251 |
| 1.790 | 1.803 | 1.814 | 1.825 | 1.835 | 1.843 | 1.851 | 1.857 | 1.861 | 1.865 | 1.867 | 1.868 | 1.867 | 1.865 | 1.861 | 1.855 | 1.848 |

| | | | | | | | | | | | | | | | | |
|---|---|---|---|---|---|---|---|---|---|---|---|---|---|---|---|---|
| 3.808 | 3.809 | 3.811 | 3.813 | 3.814 | 3.816 | 3.818 | 3.819 | 3.821 | 3.823 | 3.824 | 3.826 | 3.828 | 3.829 | 3.831 | 3.833 | 3.834 |
| −7.056 | −7.036 | −7.015 | −6.994 | −6.974 | −6.954 | −6.934 | −6.914 | −6.895 | −6.876 | −6.858 | −6.839 | −6.821 | −6.803 | −6.786 | −6.768 | −6.751 |
| 2.250 | 2.250 | 2.249 | 2.247 | 2.244 | 2.240 | 2.235 | 2.230 | 2.224 | 2.217 | 2.210 | 2.201 | 2.193 | 2.184 | 2.174 | 2.165 | 2.155 |
| −8.221 | −8.194 | −8.168 | −8.143 | −8.120 | −8.099 | −8.079 | −8.060 | −8.043 | −8.028 | −8.014 | −8.002 | −7.990 | −7.980 | −7.970 | −7.962 | −7.954 |
| 1.840 | 1.829 | 1.817 | 1.803 | 1.788 | 1.770 | 1.752 | 1.731 | 1.709 | 1.686 | 1.662 | 1.637 | 1.610 | 1.582 | 1.553 | 1.523 | 1.492 |

| | | | | | | | | | | | | | | | | |
|---|---|---|---|---|---|---|---|---|---|---|---|---|---|---|---|---|
| 3.836 | 3.838 | 3.839 | 3.841 | 3.843 | 3.844 | 3.846 | 3.848 | 3.849 | 3.851 | 3.853 | 3.854 | 3.856 | 3.858 | 3.859 | 3.861 | 3.863 |
| −6.734 | −6.717 | −6.700 | −6.683 | −6.666 | −6.650 | −6.633 | −6.617 | −6.601 | −6.584 | −6.568 | −6.551 | −6.534 | −6.516 | −6.498 | −6.480 | −6.461 |
| 2.144 | 2.134 | 2.124 | 2.114 | 2.104 | 2.094 | 2.084 | 2.075 | 2.066 | 2.057 | 2.049 | 2.041 | 2.034 | 2.027 | 2.020 | 2.014 | 2.008 |
| −7.948 | −7.942 | −7.936 | −7.932 | −7.928 | −7.925 | −7.922 | −7.919 | −7.917 | −7.914 | −7.910 | −7.906 | −7.901 | −7.896 | −7.889 | −7.881 | −7.873 |
| 1.460 | 1.428 | 1.394 | 1.361 | 1.327 | 1.293 | 1.260 | 1.226 | 1.192 | 1.159 | 1.125 | 1.092 | 1.058 | 1.025 | 0.992 | 0.958 | 0.925 |

| | | | | | | | | | | | | | | | | |
|---|---|---|---|---|---|---|---|---|---|---|---|---|---|---|---|---|
| 3.864 | 3.866 | 3.868 | 3.869 | 3.871 | 3.873 | 3.874 | 3.876 | 3.878 | 3.879 | 3.881 | 3.883 | 3.884 | 3.886 | 3.888 | 3.889 | 3.891 |
| | | | | | | | | | | | | | | | | |
| −6.441 | −6.421 | −6.400 | −6.378 | −6.355 | −6.331 | −6.305 | −6.279 | −6.250 | −6.221 | −6.190 | −6.158 | −6.125 | −6.091 | −6.056 | −6.020 | −5.983 |
| 2.002 | 1.996 | 1.990 | 1.985 | 1.980 | 1.975 | 1.970 | 1.965 | 1.960 | 1.956 | 1.951 | 1.947 | 1.943 | 1.940 | 1.937 | 1.934 | 1.931 |
| | | | | | | | | | | | | | | | | |
| −7.863 | −7.851 | −7.838 | −7.824 | −7.807 | −7.789 | −7.769 | −7.747 | −7.723 | −7.698 | −7.670 | −7.641 | −7.610 | −7.578 | −7.544 | −7.508 | −7.471 |
| 0.892 | 0.859 | 0.826 | 0.794 | 0.761 | 0.729 | 0.697 | 0.665 | 0.634 | 0.604 | 0.574 | 0.545 | 0.518 | 0.492 | 0.467 | 0.445 | 0.424 |

| | | | | | | | | | | | | | | | | |
|---|---|---|---|---|---|---|---|---|---|---|---|---|---|---|---|---|
| 3.893 | 3.894 | 3.896 | 3.898 | 3.899 | 3.901 | 3.903 | 3.904 | 3.906 | 3.908 | 3.909 | 3.911 | 3.913 | 3.914 | 3.916 | 3.918 | 3.919 |
| | | | | | | | | | | | | | | | | |
| −5.945 | −5.907 | −5.869 | −5.830 | −5.790 | −5.751 | −5.712 | −5.672 | −5.633 | −5.594 | −5.555 | −5.517 | −5.479 | −5.441 | −5.404 | −5.368 | −5.332 |
| 1.928 | 1.926 | 1.923 | 1.919 | 1.915 | 1.911 | 1.907 | 1.901 | 1.896 | 1.890 | 1.883 | 1.876 | 1.868 | 1.861 | 1.853 | 1.845 | 1.838 |
| | | | | | | | | | | | | | | | | |
| −7.432 | −7.393 | −7.351 | −7.309 | −7.266 | −7.222 | −7.176 | −7.131 | −7.085 | −7.040 | −6.993 | −6.947 | −6.900 | −6.854 | −6.807 | −6.760 | −6.713 |
| 0.404 | 0.387 | 0.372 | 0.358 | 0.346 | 0.337 | 0.329 | 0.322 | 0.318 | 0.315 | 0.313 | 0.313 | 0.315 | 0.318 | 0.323 | 0.329 | 0.337 |

| | | | | | | | | | | | | | | | | |
|---|---|---|---|---|---|---|---|---|---|---|---|---|---|---|---|---|
| 3.921 | 3.923 | 3.924 | 3.926 | 3.928 | 3.929 | 3.931 | 3.933 | 3.934 | 3.936 | 3.938 | 3.939 | 3.941 | 3.943 | 3.944 | 3.946 | 3.948 |
| −5.296 | −5.261 | −5.225 | −5.189 | −5.154 | −5.118 | −5.082 | −5.047 | −5.012 | −4.977 | −4.942 | −4.908 | −4.873 | −4.839 | −4.805 | −4.771 | −4.738 |
| 1.830 | 1.822 | 1.814 | 1.806 | 1.798 | 1.790 | 1.782 | 1.774 | 1.766 | 1.757 | 1.749 | 1.739 | 1.730 | 1.720 | 1.709 | 1.698 | 1.686 |
| −6.665 | −6.618 | −6.571 | −6.523 | −6.474 | −6.426 | −6.377 | −6.329 | −6.280 | −6.231 | −6.181 | −6.132 | −6.082 | −6.032 | −5.981 | −5.931 | −5.880 |
| 0.346 | 0.357 | 0.370 | 0.385 | 0.401 | 0.419 | 0.438 | 0.459 | 0.482 | 0.505 | 0.531 | 0.557 | 0.584 | 0.612 | 0.641 | 0.671 | 0.701 |

| | | | | | | | | | | | | | | | | |
|---|---|---|---|---|---|---|---|---|---|---|---|---|---|---|---|---|
| 3.949 | 3.951 | 3.953 | 3.954 | 3.956 | 3.958 | 3.959 | 3.961 | 3.963 | 3.964 | 3.966 | 3.968 | 3.969 | 3.971 | 3.973 | 3.974 | 3.976 |
| −4.706 | −4.674 | −4.644 | −4.614 | −4.585 | −4.557 | −4.531 | −4.505 | −4.481 | −4.457 | −4.436 | −4.415 | −4.397 | −4.380 | −4.364 | −4.350 | −4.339 |
| 1.673 | 1.660 | 1.646 | 1.632 | 1.617 | 1.602 | 1.586 | 1.569 | 1.552 | 1.534 | 1.515 | 1.496 | 1.475 | 1.454 | 1.431 | 1.408 | 1.383 |
| −5.829 | −5.779 | −5.730 | −5.681 | −5.634 | −5.587 | −5.541 | −5.497 | −5.454 | −5.412 | −5.373 | −5.336 | −5.300 | −5.267 | −5.235 | −5.206 | −5.179 |
| 0.732 | 0.763 | 0.795 | 0.826 | 0.858 | 0.889 | 0.920 | 0.950 | 0.980 | 1.010 | 1.039 | 1.066 | 1.093 | 1.119 | 1.144 | 1.167 | 1.189 |

| | | | | | | | | | | | | | | | | |
|---|---|---|---|---|---|---|---|---|---|---|---|---|---|---|---|---|
| 3.978 | 3.979 | 3.981 | 3.983 | 3.984 | 3.986 | 3.988 | 3.989 | 3.991 | 3.993 | 3.994 | 3.996 | 3.998 | 3.999 | 4.001 | 4.003 | 4.004 |
| −4.329 | −4.321 | −4.315 | −4.312 | −4.311 | −4.313 | −4.318 | −4.325 | −4.337 | −4.351 | −4.369 | −4.390 | −4.414 | −4.441 | −4.472 | −4.506 | −4.544 |
| 1.357 | 1.330 | 1.302 | 1.272 | 1.241 | 1.209 | 1.174 | 1.139 | 1.101 | 1.063 | 1.022 | 0.981 | 0.937 | 0.892 | 0.845 | 0.796 | 0.746 |
| −5.154 | −5.132 | −5.112 | −5.094 | −5.079 | −5.067 | −5.057 | −5.050 | −5.046 | −5.044 | −5.046 | −5.051 | −5.060 | −5.072 | −5.088 | −5.107 | −5.130 |
| 1.210 | 1.229 | 1.246 | 1.262 | 1.276 | 1.289 | 1.299 | 1.307 | 1.313 | 1.316 | 1.318 | 1.316 | 1.312 | 1.305 | 1.296 | 1.284 | 1.270 |

| | | | | | | | | | | | | | | | | |
|---|---|---|---|---|---|---|---|---|---|---|---|---|---|---|---|---|
| 4.006 | 4.008 | 4.009 | 4.011 | 4.013 | 4.014 | 4.016 | 4.018 | 4.019 | 4.021 | 4.023 | 4.024 | 4.026 | 4.028 | 4.029 | 4.031 | 4.033 |
| −4.585 | −4.629 | −4.677 | −4.727 | −4.781 | −4.837 | −4.896 | −4.959 | −5.024 | −5.092 | −5.163 | −5.237 | −5.314 | −5.393 | −5.476 | −5.560 | −5.647 |
| 0.695 | 0.642 | 0.587 | 0.531 | 0.473 | 0.413 | 0.352 | 0.290 | 0.226 | 0.161 | 0.094 | 0.027 | −0.043 | −0.113 | −0.184 | −0.256 | −0.329 |
| −5.156 | −5.186 | −5.220 | −5.256 | −5.297 | −5.340 | −5.386 | −5.435 | −5.488 | −5.543 | −5.601 | −5.661 | −5.724 | −5.790 | −5.858 | −5.929 | −6.002 |
| 1.252 | 1.232 | 1.210 | 1.185 | 1.158 | 1.128 | 1.095 | 1.061 | 1.024 | 0.986 | 0.945 | 0.903 | 0.858 | 0.812 | 0.763 | 0.713 | 0.661 |

| | | | | | | | | | | | | | | | | |
|---|---|---|---|---|---|---|---|---|---|---|---|---|---|---|---|---|
| 4.034 | 4.036 | 4.038 | 4.039 | 4.041 | 4.043 | 4.044 | 4.046 | 4.048 | 4.049 | 4.051 | 4.053 | 4.054 | 4.056 | 4.058 | 4.059 | 4.061 |
| −5.737 | −5.828 | −5.922 | −6.017 | −6.115 | −6.215 | −6.316 | −6.419 | −6.523 | −6.630 | −6.737 | −6.846 | −6.956 | −7.068 | −7.180 | −7.294 | −7.407 |
| −0.402 | −0.477 | −0.552 | −0.628 | −0.704 | −0.781 | −0.858 | −0.936 | −1.015 | −1.094 | −1.173 | −1.252 | −1.331 | −1.410 | −1.489 | −1.567 | −1.646 |
| −6.077 | −6.155 | −6.235 | −6.318 | −6.402 | −6.489 | −6.577 | −6.666 | −6.758 | −6.851 | −6.946 | −7.042 | −7.139 | −7.238 | −7.338 | −7.438 | −7.540 |
| 0.607 | 0.551 | 0.494 | 0.434 | 0.373 | 0.309 | 0.244 | 0.177 | 0.108 | 0.038 | −0.034 | −0.107 | −0.182 | −0.258 | −0.334 | −0.412 | −0.490 |

| | | | | | | | | | | | | | | | | |
|---|---|---|---|---|---|---|---|---|---|---|---|---|---|---|---|---|
| 4.063 | 4.064 | 4.066 | 4.067 | 4.069 | 4.071 | 4.072 | 4.074 | 4.076 | 4.077 | 4.079 | 4.081 | 4.082 | 4.084 | 4.086 | 4.087 | 4.089 |
| | | | | | | | | | | | | | | | | |
| −7.521 | −7.635 | −7.749 | −7.863 | −7.976 | −8.088 | −8.200 | −8.310 | −8.419 | −8.526 | −8.631 | −8.734 | −8.835 | −8.934 | −9.031 | −9.125 | −9.217 |
| −1.723 | −1.800 | −1.877 | −1.953 | −2.028 | −2.103 | −2.176 | −2.249 | −2.321 | −2.392 | −2.462 | −2.530 | −2.598 | −2.663 | −2.728 | −2.790 | −2.851 |
| | | | | | | | | | | | | | | | | |
| −7.642 | −7.744 | −7.847 | −7.950 | −8.052 | −8.154 | −8.255 | −8.356 | −8.456 | −8.555 | −8.652 | −8.747 | −8.841 | −8.933 | −9.022 | −9.111 | −9.197 |
| −0.569 | −0.649 | −0.729 | −0.810 | −0.890 | −0.971 | −1.052 | −1.134 | −1.215 | −1.295 | −1.376 | −1.456 | −1.536 | −1.615 | −1.693 | −1.771 | −1.848 |

| | | | | | | | | | | | | | | | | |
|---|---|---|---|---|---|---|---|---|---|---|---|---|---|---|---|---|
| 4.091 | 4.092 | 4.094 | 4.096 | 4.097 | 4.099 | 4.101 | 4.102 | 4.104 | 4.106 | 4.107 | 4.109 | 4.111 | 4.112 | 4.114 | 4.116 | 4.117 |
| | | | | | | | | | | | | | | | | |
| −9.307 | −9.394 | −9.480 | −9.563 | −9.643 | −9.722 | −9.798 | −9.872 | −9.944 | −10.014 | −10.082 | −10.148 | −10.211 | −10.273 | −10.332 | −10.389 | −10.444 |
| −2.910 | −2.967 | −3.023 | −3.076 | −3.127 | −3.176 | −3.223 | −3.268 | −3.310 | −3.350 | −3.387 | −3.423 | −3.456 | −3.488 | −3.517 | −3.544 | −3.569 |
| | | | | | | | | | | | | | | | | |
| −9.282 | −9.366 | −9.448 | −9.529 | −9.609 | −9.687 | −9.765 | −9.841 | −9.916 | −9.991 | −10.065 | −10.138 | −10.210 | −10.282 | −10.351 | −10.420 | −10.487 |
| −1.923 | −1.998 | −2.072 | −2.145 | −2.215 | −2.285 | −2.352 | −2.418 | −2.482 | −2.545 | −2.605 | −2.663 | −2.720 | −2.774 | −2.827 | −2.878 | −2.926 |

| | | | | | | | | | | | | | | | | |
|---|---|---|---|---|---|---|---|---|---|---|---|---|---|---|---|---|
| 4.119 | 4.121 | 4.122 | 4.124 | 4.126 | 4.127 | 4.129 | 4.131 | 4.132 | 4.134 | 4.136 | 4.137 | 4.139 | 4.141 | 4.142 | 4.144 | 4.146 |
| −10.496 | −10.547 | −10.595 | −10.641 | −10.684 | −10.725 | −10.763 | −10.798 | −10.831 | −10.862 | −10.890 | −10.916 | −10.939 | −10.961 | −10.980 | −10.998 | −11.014 |
| −3.592 | −3.613 | −3.632 | −3.650 | −3.666 | −3.680 | −3.693 | −3.705 | −3.714 | −3.723 | −3.729 | −3.735 | −3.739 | −3.742 | −3.743 | −3.742 | −3.740 |
| −10.553 | −10.616 | −10.679 | −10.740 | −10.799 | −10.856 | −10.912 | −10.966 | −11.018 | −11.068 | −11.117 | −11.165 | −11.211 | −11.257 | −11.301 | −11.344 | −11.386 |
| −2.973 | −3.018 | −3.060 | −3.101 | −3.139 | −3.176 | −3.210 | −3.241 | −3.271 | −3.298 | −3.323 | −3.346 | −3.367 | −3.387 | −3.404 | −3.419 | −3.433 |

| | | | | | | | | | | | | | | | | |
|---|---|---|---|---|---|---|---|---|---|---|---|---|---|---|---|---|
| 4.147 | 4.149 | 4.151 | 4.152 | 4.154 | 4.156 | 4.157 | 4.159 | 4.161 | 4.162 | 4.164 | 4.166 | 4.167 | 4.169 | 4.171 | 4.172 | 4.174 |
| | | | | | | | | | | | | | | | | |
| −11.028 | −11.040 | −11.051 | −11.060 | −11.067 | −11.072 | −11.076 | −11.077 | −11.077 | −11.074 | −11.070 | −11.064 | −11.057 | −11.047 | −11.036 | −11.024 | −11.010 |
| −3.737 | −3.732 | −3.725 | −3.717 | −3.707 | −3.696 | −3.683 | −3.669 | −3.654 | −3.638 | −3.620 | −3.601 | −3.581 | −3.560 | −3.537 | −3.513 | −3.488 |
| | | | | | | | | | | | | | | | | |
| −11.426 | −11.465 | −11.503 | −11.540 | −11.575 | −11.609 | −11.641 | −11.672 | −11.701 | −11.728 | −11.753 | −11.776 | −11.798 | −11.818 | −11.836 | −11.852 | −11.866 |
| −3.445 | −3.455 | −3.464 | −3.470 | −3.475 | −3.478 | −3.479 | −3.478 | −3.476 | −3.472 | −3.467 | −3.459 | −3.451 | −3.440 | −3.429 | −3.415 | −3.401 |

| | | | | | | | | | | | | | | | | |
|---|---|---|---|---|---|---|---|---|---|---|---|---|---|---|---|---|
| 4.176 | 4.177 | 4.179 | 4.181 | 4.182 | 4.184 | 4.186 | 4.187 | 4.189 | 4.191 | 4.192 | 4.194 | 4.196 | 4.197 | 4.199 | 4.201 | 4.202 |
| −10.994 | −10.976 | −10.957 | −10.936 | −10.914 | −10.890 | −10.865 | −10.837 | −10.809 | −10.779 | −10.748 | −10.715 | −10.682 | −10.647 | −10.611 | −10.574 | −10.537 |
| −3.462 | −3.435 | −3.407 | −3.377 | −3.346 | −3.314 | −3.280 | −3.246 | −3.210 | −3.174 | −3.137 | −3.098 | −3.059 | −3.019 | −2.979 | −2.937 | −2.895 |
| −11.878 | −11.887 | −11.895 | −11.901 | −11.905 | −11.907 | −11.908 | −11.906 | −11.903 | −11.897 | −11.890 | −11.881 | −11.870 | −11.857 | −11.843 | −11.827 | −11.809 |
| −3.385 | −3.368 | −3.350 | −3.330 | −3.309 | −3.287 | −3.265 | −3.241 | −3.216 | −3.190 | −3.164 | −3.136 | −3.108 | −3.078 | −3.048 | −3.018 | −2.987 |

| | | | | | | | | | | | | | | | | |
|---|---|---|---|---|---|---|---|---|---|---|---|---|---|---|---|---|
| 4.204 | 4.206 | 4.207 | 4.209 | 4.211 | 4.212 | 4.214 | 4.216 | 4.217 | 4.219 | 4.221 | 4.222 | 4.224 | 4.226 | 4.227 | 4.229 | 4.231 |
| −10.499 | −10.460 | −10.421 | −10.381 | −10.341 | −10.301 | −10.260 | −10.220 | −10.179 | −10.138 | −10.096 | −10.055 | −10.013 | −9.972 | −9.930 | −9.889 | −9.848 |
| −2.852 | −2.808 | −2.764 | −2.719 | −2.673 | −2.628 | −2.582 | −2.536 | −2.490 | −2.444 | −2.399 | −2.353 | −2.309 | −2.264 | −2.220 | −2.177 | −2.133 |
| −11.790 | −11.769 | −11.748 | −11.725 | −11.701 | −11.677 | −11.651 | −11.625 | −11.598 | −11.570 | −11.543 | −11.514 | −11.485 | −11.456 | −11.426 | −11.396 | −11.366 |
| −2.956 | −2.925 | −2.893 | −2.861 | −2.830 | −2.798 | −2.766 | −2.734 | −2.702 | −2.669 | −2.637 | −2.604 | −2.572 | −2.539 | −2.507 | −2.475 | −2.443 |

| | | | | | | | | | | | | | | | | |
|---|---|---|---|---|---|---|---|---|---|---|---|---|---|---|---|---|
| 4.232 | 4.234 | 4.236 | 4.237 | 4.239 | 4.241 | 4.242 | 4.244 | 4.246 | 4.247 | 4.249 | 4.251 | 4.252 | 4.254 | 4.256 | 4.257 | 4.259 |
| −9.807 | −9.766 | −9.726 | −9.685 | −9.645 | −9.604 | −9.563 | −9.522 | −9.481 | −9.441 | −9.400 | −9.360 | −9.321 | −9.282 | −9.245 | −9.208 | −9.172 |
| −2.090 | −2.047 | −2.005 | −1.963 | −1.921 | −1.880 | −1.839 | −1.798 | −1.757 | −1.717 | −1.678 | −1.639 | −1.600 | −1.562 | −1.525 | −1.488 | −1.452 |
| −11.337 | −11.307 | −11.277 | −11.248 | −11.218 | −11.189 | −11.160 | −11.132 | −11.104 | −11.076 | −11.049 | −11.023 | −10.997 | −10.972 | −10.948 | −10.925 | −10.902 |
| −2.411 | −2.380 | −2.349 | −2.318 | −2.288 | −2.258 | −2.228 | −2.198 | −2.168 | −2.139 | −2.109 | −2.079 | −2.048 | −2.018 | −1.987 | −1.957 | −1.926 |

| | | | | | | | | | | | | | | | | |
|---|---|---|---|---|---|---|---|---|---|---|---|---|---|---|---|---|
| 4.261 | 4.262 | 4.264 | 4.266 | 4.267 | 4.269 | 4.271 | 4.272 | 4.274 | 4.276 | 4.277 | 4.279 | 4.281 | 4.282 | 4.284 | 4.286 | 4.287 |
| −9.137 | −9.104 | −9.071 | −9.040 | −9.010 | −8.982 | −8.955 | −8.929 | −8.905 | −8.883 | −8.863 | −8.844 | −8.826 | −8.811 | −8.797 | −8.785 | −8.774 |
| −1.416 | −1.381 | −1.346 | −1.312 | −1.279 | −1.247 | −1.214 | −1.183 | −1.152 | −1.121 | −1.091 | −1.061 | −1.031 | −1.001 | −0.972 | −0.942 | −0.913 |
| −10.880 | −10.859 | −10.839 | −10.819 | −10.800 | −10.782 | −10.764 | −10.747 | −10.730 | −10.714 | −10.698 | −10.683 | −10.668 | −10.654 | −10.642 | −10.630 | −10.619 |
| −1.895 | −1.865 | −1.834 | −1.803 | −1.772 | −1.741 | −1.710 | −1.680 | −1.649 | −1.619 | −1.590 | −1.560 | −1.532 | −1.503 | −1.475 | −1.448 | −1.420 |

| | | | | | | | | | | | | | | | | |
|---|---|---|---|---|---|---|---|---|---|---|---|---|---|---|---|---|
| 4.289 | 4.291 | 4.292 | 4.294 | 4.296 | 4.297 | 4.299 | 4.301 | 4.302 | 4.304 | 4.306 | 4.307 | 4.309 | 4.311 | 4.312 | 4.314 | 4.316 |
| | | | | | | | | | | | | | | | | |
| −8.765 | −8.757 | −8.751 | −8.746 | −8.742 | −8.740 | −8.739 | −8.740 | −8.742 | −8.745 | −8.750 | −8.755 | −8.762 | −8.770 | −8.779 | −8.789 | −8.800 |
| −0.884 | −0.855 | −0.826 | −0.797 | −0.768 | −0.740 | −0.711 | −0.682 | −0.653 | −0.624 | −0.595 | −0.566 | −0.536 | −0.506 | −0.475 | −0.443 | −0.411 |
| | | | | | | | | | | | | | | | | |
| −10.608 | −10.599 | −10.591 | −10.583 | −10.577 | −10.571 | −10.566 | −10.562 | −10.558 | −10.555 | −10.552 | −10.550 | −10.549 | −10.547 | −10.546 | −10.546 | −10.546 |
| −1.393 | −1.365 | −1.338 | −1.311 | −1.283 | −1.256 | −1.229 | −1.201 | −1.173 | −1.145 | −1.117 | −1.088 | −1.059 | −1.029 | −0.998 | −0.967 | −0.935 |

| | | | | | | | | | | | | | | | | |
|---|---|---|---|---|---|---|---|---|---|---|---|---|---|---|---|---|
| 4.317 | 4.319 | 4.321 | 4.322 | 4.324 | 4.326 | 4.327 | 4.329 | 4.331 | 4.332 | 4.334 | 4.336 | 4.337 | 4.339 | 4.341 | 4.342 | 4.344 |
| | | | | | | | | | | | | | | | | |
| −8.811 | −8.824 | −8.837 | −8.851 | −8.865 | −8.879 | −8.894 | −8.909 | −8.924 | −8.938 | −8.953 | −8.968 | −8.983 | −8.998 | −9.013 | −9.028 | −9.043 |
| −0.378 | −0.345 | −0.310 | −0.275 | −0.240 | −0.204 | −0.168 | −0.131 | −0.094 | −0.056 | −0.018 | 0.020 | 0.059 | 0.099 | 0.138 | 0.178 | 0.218 |
| | | | | | | | | | | | | | | | | |
| −10.545 | −10.545 | −10.545 | −10.544 | −10.543 | −10.541 | −10.540 | −10.538 | −10.537 | −10.535 | −10.534 | −10.532 | −10.531 | −10.530 | −10.529 | −10.529 | −10.529 |
| −0.902 | −0.869 | −0.834 | −0.799 | −0.764 | −0.727 | −0.690 | −0.653 | −0.614 | −0.575 | −0.535 | −0.494 | −0.453 | −0.411 | −0.368 | −0.324 | −0.280 |

| | | | | | | | | | | | | | | | | |
|---|---|---|---|---|---|---|---|---|---|---|---|---|---|---|---|---|
| 4.346 | 4.347 | 4.349 | 4.351 | 4.352 | 4.354 | 4.356 | 4.357 | 4.359 | 4.361 | 4.362 | 4.364 | 4.366 | 4.367 | 4.369 | 4.371 | 4.372 |
| −9.058 | −9.073 | −9.088 | −9.102 | −9.116 | −9.130 | −9.144 | −9.158 | −9.170 | −9.183 | −9.196 | −9.208 | −9.221 | −9.233 | −9.244 | −9.255 | −9.266 |
| 0.258 | 0.297 | 0.337 | 0.376 | 0.414 | 0.452 | 0.489 | 0.526 | 0.561 | 0.595 | 0.628 | 0.659 | 0.690 | 0.718 | 0.746 | 0.771 | 0.796 |
| −10.529 | −10.529 | −10.529 | −10.530 | −10.531 | −10.532 | −10.532 | −10.534 | −10.535 | −10.536 | −10.538 | −10.539 | −10.541 | −10.543 | −10.545 | −10.547 | −10.550 |
| −0.235 | −0.190 | −0.144 | −0.097 | −0.050 | −0.003 | 0.044 | 0.091 | 0.138 | 0.184 | 0.230 | 0.275 | 0.320 | 0.364 | 0.407 | 0.449 | 0.489 |

| | | | | | | | | | | | | | | | | |
|---|---|---|---|---|---|---|---|---|---|---|---|---|---|---|---|---|
| 4.374 | 4.376 | 4.377 | 4.379 | 4.381 | 4.382 | 4.384 | 4.386 | 4.387 | 4.389 | 4.390 | 4.392 | 4.394 | 4.395 | 4.397 | 4.399 | 4.400 |
| | | | | | | | | | | | | | | | | |
| −9.276 | −9.286 | −9.296 | −9.305 | −9.314 | −9.322 | −9.329 | −9.335 | −9.341 | −9.345 | −9.349 | −9.351 | −9.352 | −9.353 | −9.352 | −9.350 | −9.348 |
| 0.818 | 0.840 | 0.860 | 0.878 | 0.895 | 0.910 | 0.924 | 0.937 | 0.948 | 0.957 | 0.966 | 0.973 | 0.979 | 0.983 | 0.987 | 0.990 | 0.992 |
| | | | | | | | | | | | | | | | | |
| −10.554 | −10.557 | −10.562 | −10.566 | −10.571 | −10.576 | −10.582 | −10.588 | −10.594 | −10.601 | −10.608 | −10.615 | −10.623 | −10.630 | −10.637 | −10.645 | −10.653 |
| 0.528 | 0.566 | 0.602 | 0.637 | 0.670 | 0.701 | 0.730 | 0.757 | 0.781 | 0.804 | 0.824 | 0.843 | 0.859 | 0.873 | 0.885 | 0.894 | 0.902 |

| | | | | | | | | | | | | | | | | |
|---|---|---|---|---|---|---|---|---|---|---|---|---|---|---|---|---|
| 4.402 | 4.404 | 4.405 | 4.407 | 4.409 | 4.410 | 4.412 | 4.414 | 4.415 | 4.417 | 4.419 | 4.420 | 4.422 | 4.424 | 4.425 | 4.427 | 4.429 |
| −9.345 | −9.340 | −9.335 | −9.328 | −9.320 | −9.311 | −9.300 | −9.287 | −9.273 | −9.256 | −9.237 | −9.217 | −9.194 | −9.170 | −9.144 | −9.117 | −9.088 |
| 0.994 | 0.994 | 0.994 | 0.994 | 0.992 | 0.990 | 0.987 | 0.984 | 0.980 | 0.975 | 0.969 | 0.963 | 0.956 | 0.948 | 0.939 | 0.930 | 0.921 |
| −10.661 | −10.668 | −10.676 | −10.683 | −10.690 | −10.696 | −10.702 | −10.707 | −10.711 | −10.714 | −10.715 | −10.715 | −10.714 | −10.712 | −10.708 | −10.703 | −10.697 |
| 0.907 | 0.911 | 0.912 | 0.912 | 0.910 | 0.906 | 0.901 | 0.894 | 0.886 | 0.876 | 0.865 | 0.852 | 0.838 | 0.822 | 0.805 | 0.786 | 0.767 |

| | | | | | | | | | | | | | | | | |
|---|---|---|---|---|---|---|---|---|---|---|---|---|---|---|---|---|
| 4.430 | 4.432 | 4.434 | 4.435 | 4.437 | 4.439 | 4.440 | 4.442 | 4.444 | 4.445 | 4.447 | 4.449 | 4.450 | 4.452 | 4.454 | 4.455 | 4.457 |
| −9.057 | −9.024 | −8.990 | −8.954 | −8.917 | −8.879 | −8.840 | −8.800 | −8.760 | −8.719 | −8.678 | −8.636 | −8.593 | −8.550 | −8.506 | −8.462 | −8.418 |
| 0.911 | 0.900 | 0.889 | 0.878 | 0.866 | 0.854 | 0.841 | 0.828 | 0.814 | 0.800 | 0.785 | 0.769 | 0.753 | 0.737 | 0.720 | 0.702 | 0.684 |
| −10.689 | −10.680 | −10.669 | −10.656 | −10.642 | −10.625 | −10.607 | −10.587 | −10.566 | −10.542 | −10.516 | −10.488 | −10.459 | −10.427 | −10.395 | −10.361 | −10.326 |
| 0.746 | 0.724 | 0.700 | 0.676 | 0.650 | 0.624 | 0.597 | 0.569 | 0.540 | 0.510 | 0.480 | 0.449 | 0.417 | 0.384 | 0.351 | 0.317 | 0.283 |

| | | | | | | | | | | | | | | | | |
|---|---|---|---|---|---|---|---|---|---|---|---|---|---|---|---|---|
| 4.459 | 4.460 | 4.462 | 4.464 | 4.465 | 4.467 | 4.469 | 4.470 | 4.472 | 4.474 | 4.475 | 4.477 | 4.479 | 4.480 | 4.482 | 4.484 | 4.485 |
| −8.373 | −8.329 | −8.284 | −8.239 | −8.194 | −8.148 | −8.103 | −8.058 | −8.013 | −7.968 | −7.924 | −7.880 | −7.836 | −7.793 | −7.750 | −7.709 | −7.668 |
| 0.666 | 0.647 | 0.628 | 0.609 | 0.591 | 0.572 | 0.552 | 0.533 | 0.514 | 0.495 | 0.475 | 0.456 | 0.437 | 0.418 | 0.399 | 0.380 | 0.362 |
| −10.290 | −10.253 | −10.215 | −10.177 | −10.138 | −10.099 | −10.060 | −10.020 | −9.980 | −9.940 | −9.899 | −9.859 | −9.819 | −9.779 | −9.739 | −9.700 | −9.660 |
| 0.249 | 0.215 | 0.180 | 0.146 | 0.112 | 0.078 | 0.045 | 0.011 | −0.022 | −0.054 | −0.087 | −0.119 | −0.151 | −0.183 | −0.214 | −0.245 | −0.275 |

| | | | | | | | | | | | | | | | | |
|---|---|---|---|---|---|---|---|---|---|---|---|---|---|---|---|---|
| 4.487 | 4.489 | 4.490 | 4.492 | 4.494 | 4.495 | 4.497 | 4.499 | 4.500 | 4.502 | 4.504 | 4.505 | 4.507 | 4.509 | 4.510 | 4.512 | 4.514 |
| −7.628 | −7.588 | −7.550 | −7.512 | −7.476 | −7.440 | −7.406 | −7.373 | −7.341 | −7.310 | −7.280 | −7.252 | −7.224 | −7.198 | −7.172 | −7.148 | −7.124 |
| 0.343 | 0.324 | 0.305 | 0.286 | 0.267 | 0.248 | 0.228 | 0.209 | 0.189 | 0.169 | 0.149 | 0.129 | 0.108 | 0.087 | 0.066 | 0.045 | 0.023 |
| −9.620 | −9.581 | −9.541 | −9.501 | −9.461 | −9.421 | −9.381 | −9.341 | −9.301 | −9.261 | −9.221 | −9.181 | −9.141 | −9.102 | −9.062 | −9.023 | −8.984 |
| −0.304 | −0.333 | −0.362 | −0.390 | −0.417 | −0.444 | −0.470 | −0.495 | −0.521 | −0.545 | −0.570 | −0.594 | −0.618 | −0.642 | −0.665 | −0.688 | −0.711 |

| | | | | | | | | | | | | | | | | |
|---|---|---|---|---|---|---|---|---|---|---|---|---|---|---|---|---|
| 4.515 | 4.517 | 4.519 | 4.520 | 4.522 | 4.524 | 4.525 | 4.527 | 4.529 | 4.530 | 4.532 | 4.534 | 4.535 | 4.537 | 4.539 | 4.540 | 4.542 |
| −7.102 | −7.081 | −7.061 | −7.043 | −7.026 | −7.011 | −6.998 | −6.987 | −6.978 | −6.970 | −6.965 | −6.962 | −6.962 | −6.963 | −6.967 | −6.973 | −6.981 |
| 0.000 | −0.022 | −0.045 | −0.068 | −0.091 | −0.114 | −0.137 | −0.160 | −0.183 | −0.205 | −0.228 | −0.250 | −0.271 | −0.292 | −0.313 | −0.333 | −0.352 |
| −8.945 | −8.906 | −8.868 | −8.829 | −8.792 | −8.754 | −8.718 | −8.683 | −8.648 | −8.615 | −8.583 | −8.552 | −8.522 | −8.493 | −8.466 | −8.439 | −8.414 |
| −0.734 | −0.756 | −0.778 | −0.800 | −0.822 | −0.843 | −0.864 | −0.884 | −0.904 | −0.923 | −0.942 | −0.961 | −0.979 | −0.996 | −1.013 | −1.030 | −1.046 |

| | | | | | | | | | | | | | | | | |
|---|---|---|---|---|---|---|---|---|---|---|---|---|---|---|---|---|
| 4.544 | 4.545 | 4.547 | 4.549 | 4.550 | 4.552 | 4.554 | 4.555 | 4.557 | 4.559 | 4.560 | 4.562 | 4.564 | 4.565 | 4.567 | 4.569 | 4.570 |
| | | | | | | | | | | | | | | | | |
| −6.992 | −7.004 | −7.019 | −7.037 | −7.056 | −7.079 | −7.103 | −7.130 | −7.159 | −7.190 | −7.223 | −7.259 | −7.296 | −7.336 | −7.378 | −7.422 | −7.468 |
| −0.370 | −0.388 | −0.405 | −0.422 | −0.438 | −0.453 | −0.468 | −0.482 | −0.496 | −0.510 | −0.523 | −0.536 | −0.549 | −0.560 | −0.571 | −0.581 | −0.591 |
| | | | | | | | | | | | | | | | | |
| −8.389 | −8.366 | −8.344 | −8.324 | −8.305 | −8.288 | −8.273 | −8.259 | −8.248 | −8.238 | −8.230 | −8.224 | −8.219 | −8.216 | −8.215 | −8.216 | −8.218 |
| −1.062 | −1.077 | −1.092 | −1.107 | −1.122 | −1.137 | −1.151 | −1.165 | −1.180 | −1.194 | −1.208 | −1.222 | −1.236 | −1.250 | −1.264 | −1.279 | −1.293 |

| | | | | | | | | | | | | | | | | |
|---|---|---|---|---|---|---|---|---|---|---|---|---|---|---|---|---|
| 4.572 | 4.574 | 4.575 | 4.577 | 4.579 | 4.580 | 4.582 | 4.584 | 4.585 | 4.587 | 4.589 | 4.590 | 4.592 | 4.594 | 4.595 | 4.597 | 4.599 |
| | | | | | | | | | | | | | | | | |
| −7.515 | −7.564 | −7.614 | −7.665 | −7.717 | −7.770 | −7.824 | −7.878 | −7.933 | −7.988 | −8.044 | −8.100 | −8.156 | −8.212 | −8.268 | −8.324 | −8.380 |
| −0.599 | −0.608 | −0.616 | −0.623 | −0.630 | −0.637 | −0.643 | −0.649 | −0.655 | −0.660 | −0.666 | −0.671 | −0.677 | −0.682 | −0.688 | −0.694 | −0.700 |
| | | | | | | | | | | | | | | | | |
| −8.222 | −8.227 | −8.233 | −8.241 | −8.249 | −8.259 | −8.269 | −8.280 | −8.293 | −8.306 | −8.321 | −8.337 | −8.354 | −8.371 | −8.390 | −8.410 | −8.431 |
| −1.307 | −1.322 | −1.337 | −1.352 | −1.367 | −1.382 | −1.398 | −1.414 | −1.429 | −1.445 | −1.460 | −1.475 | −1.490 | −1.505 | −1.520 | −1.534 | −1.548 |

| | | | | | | | | | | | | | | | | |
|---|---|---|---|---|---|---|---|---|---|---|---|---|---|---|---|---|
| 4.600 | 4.602 | 4.604 | 4.605 | 4.607 | 4.609 | 4.610 | 4.612 | 4.614 | 4.615 | 4.617 | 4.619 | 4.620 | 4.622 | 4.624 | 4.625 | 4.627 |
| −8.435 | −8.490 | −8.545 | −8.599 | −8.652 | −8.705 | −8.757 | −8.808 | −8.858 | −8.907 | −8.955 | −9.002 | −9.047 | −9.092 | −9.136 | −9.178 | −9.220 |
| −0.707 | −0.714 | −0.722 | −0.729 | −0.737 | −0.745 | −0.754 | −0.762 | −0.771 | −0.780 | −0.789 | −0.799 | −0.809 | −0.819 | −0.829 | −0.839 | −0.850 |
| −8.453 | −8.476 | −8.501 | −8.526 | −8.552 | −8.580 | −8.608 | −8.638 | −8.668 | −8.700 | −8.733 | −8.767 | −8.802 | −8.838 | −8.875 | −8.912 | −8.950 |
| −1.562 | −1.575 | −1.588 | −1.600 | −1.613 | −1.626 | −1.639 | −1.652 | −1.665 | −1.679 | −1.692 | −1.706 | −1.721 | −1.735 | −1.749 | −1.764 | −1.778 |

| | | | | | | | | | | | | | | | | |
|---|---|---|---|---|---|---|---|---|---|---|---|---|---|---|---|---|
| 4.629 | 4.630 | 4.632 | 4.634 | 4.635 | 4.637 | 4.639 | 4.640 | 4.642 | 4.644 | 4.645 | 4.647 | 4.649 | 4.650 | 4.652 | 4.654 | 4.655 |
| | | | | | | | | | | | | | | | | |
| −9.260 | −9.300 | −9.338 | −9.374 | −9.410 | −9.444 | −9.477 | −9.507 | −9.536 | −9.563 | −9.588 | −9.612 | −9.633 | −9.652 | −9.670 | −9.686 | −9.700 |
| −0.862 | −0.875 | −0.888 | −0.901 | −0.916 | −0.931 | −0.948 | −0.965 | −0.983 | −1.003 | −1.023 | −1.045 | −1.067 | −1.091 | −1.116 | −1.142 | −1.169 |
| | | | | | | | | | | | | | | | | |
| −8.989 | −9.028 | −9.066 | −9.106 | −9.145 | −9.184 | −9.223 | −9.262 | −9.300 | −9.338 | −9.377 | −9.415 | −9.452 | −9.489 | −9.525 | −9.562 | −9.598 |
| −1.793 | −1.807 | −1.822 | −1.836 | −1.851 | −1.865 | −1.880 | −1.894 | −1.909 | −1.924 | −1.938 | −1.953 | −1.967 | −1.981 | −1.996 | −2.010 | −2.024 |

| | | | | | | | | | | | | | | | | |
|---|---|---|---|---|---|---|---|---|---|---|---|---|---|---|---|---|
| 4.657 | 4.659 | 4.660 | 4.662 | 4.664 | 4.665 | 4.667 | 4.669 | 4.670 | 4.672 | 4.674 | 4.675 | 4.677 | 4.679 | 4.680 | 4.682 | 4.684 |
| | | | | | | | | | | | | | | | | |
| −9.713 | −9.723 | −9.733 | −9.740 | −9.746 | −9.750 | −9.753 | −9.755 | −9.756 | −9.755 | −9.754 | −9.753 | −9.751 | −9.749 | −9.746 | −9.743 | −9.741 |
| −1.197 | −1.227 | −1.258 | −1.289 | −1.322 | −1.355 | −1.388 | −1.422 | −1.457 | −1.491 | −1.526 | −1.561 | −1.596 | −1.632 | −1.667 | −1.702 | −1.737 |
| | | | | | | | | | | | | | | | | |
| −9.633 | −9.668 | −9.703 | −9.738 | −9.772 | −9.807 | −9.840 | −9.874 | −9.908 | −9.941 | −9.974 | −10.008 | −10.040 | −10.073 | −10.105 | −10.136 | −10.167 |
| −2.038 | −2.051 | −2.065 | −2.078 | −2.091 | −2.104 | −2.116 | −2.127 | −2.138 | −2.148 | −2.158 | −2.167 | −2.175 | −2.183 | −2.191 | −2.199 | −2.206 |

| | | | | | | | | | | | | | | | | |
|---|---|---|---|---|---|---|---|---|---|---|---|---|---|---|---|---|
| 4.685 | 4.687 | 4.689 | 4.690 | 4.692 | 4.694 | 4.695 | 4.697 | 4.699 | 4.700 | 4.702 | 4.704 | 4.705 | 4.707 | 4.709 | 4.710 | 4.712 |
| −9.738 | −9.735 | −9.731 | −9.728 | −9.725 | −9.721 | −9.717 | −9.713 | −9.709 | −9.705 | −9.700 | −9.696 | −9.691 | −9.686 | −9.681 | −9.676 | −9.671 |
| −1.772 | −1.806 | −1.840 | −1.873 | −1.906 | −1.939 | −1.971 | −2.003 | −2.034 | −2.064 | −2.093 | −2.122 | −2.150 | −2.177 | −2.203 | −2.228 | −2.251 |
| −10.196 | −10.225 | −10.252 | −10.279 | −10.304 | −10.328 | −10.351 | −10.372 | −10.391 | −10.408 | −10.424 | −10.437 | −10.448 | −10.458 | −10.465 | −10.470 | −10.474 |
| −2.214 | −2.221 | −2.229 | −2.236 | −2.243 | −2.250 | −2.256 | −2.263 | −2.270 | −2.276 | −2.283 | −2.289 | −2.295 | −2.301 | −2.307 | −2.313 | −2.319 |

| | | | | | | | | | | | | | | | | |
|---|---|---|---|---|---|---|---|---|---|---|---|---|---|---|---|---|
| 4.714 | 4.715 | 4.717 | 4.718 | 4.720 | 4.722 | 4.723 | 4.725 | 4.727 | 4.728 | 4.730 | 4.732 | 4.733 | 4.735 | 4.737 | 4.738 | 4.740 |
| | | | | | | | | | | | | | | | | |
| −9.665 | −9.660 | −9.654 | −9.648 | −9.642 | −9.635 | −9.628 | −9.620 | −9.612 | −9.604 | −9.594 | −9.584 | −9.573 | −9.561 | −9.548 | −9.535 | −9.520 |
| −2.274 | −2.295 | −2.316 | −2.334 | −2.352 | −2.369 | −2.385 | −2.399 | −2.413 | −2.426 | −2.438 | −2.449 | −2.459 | −2.468 | −2.476 | −2.484 | −2.490 |
| | | | | | | | | | | | | | | | | |
| −10.475 | −10.475 | −10.473 | −10.470 | −10.464 | −10.457 | −10.449 | −10.439 | −10.428 | −10.416 | −10.403 | −10.388 | −10.372 | −10.356 | −10.338 | −10.320 | −10.301 |
| −2.324 | −2.330 | −2.335 | −2.339 | −2.344 | −2.349 | −2.353 | −2.358 | −2.364 | −2.370 | −2.377 | −2.384 | −2.392 | −2.400 | −2.410 | −2.419 | −2.430 |

| | | | | | | | | | | | | | | | | |
|---|---|---|---|---|---|---|---|---|---|---|---|---|---|---|---|---|
| 4.742 | 4.743 | 4.745 | 4.747 | 4.748 | 4.750 | 4.752 | 4.753 | 4.755 | 4.757 | 4.758 | 4.760 | 4.762 | 4.763 | 4.765 | 4.767 | 4.768 |
| | | | | | | | | | | | | | | | | |
| −9.505 | −9.489 | −9.471 | −9.452 | −9.433 | −9.412 | −9.389 | −9.365 | −9.341 | −9.315 | −9.288 | −9.259 | −9.230 | −9.200 | −9.168 | −9.136 | −9.102 |
| −2.497 | −2.503 | −2.509 | −2.515 | −2.520 | −2.525 | −2.531 | −2.535 | −2.541 | −2.545 | −2.550 | −2.555 | −2.560 | −2.564 | −2.570 | −2.575 | −2.580 |
| | | | | | | | | | | | | | | | | |
| −10.281 | −10.261 | −10.240 | −10.219 | −10.197 | −10.174 | −10.151 | −10.127 | −10.102 | −10.076 | −10.050 | −10.023 | −9.996 | −9.968 | −9.940 | −9.911 | −9.883 |
| −2.440 | −2.452 | −2.464 | −2.477 | −2.491 | −2.505 | −2.520 | −2.536 | −2.553 | −2.571 | −2.590 | −2.609 | −2.629 | −2.650 | −2.672 | −2.694 | −2.718 |

| | | | | | | | | | | | | | | | | |
|---|---|---|---|---|---|---|---|---|---|---|---|---|---|---|---|---|
| 4.770 | 4.772 | 4.773 | 4.775 | 4.777 | 4.778 | 4.780 | 4.782 | 4.783 | 4.785 | 4.787 | 4.788 | 4.790 | 4.792 | 4.793 | 4.795 | 4.797 |
| −9.068 | −9.032 | −8.997 | −8.960 | −8.924 | −8.887 | −8.851 | −8.814 | −8.778 | −8.741 | −8.706 | −8.670 | −8.634 | −8.599 | −8.565 | −8.531 | −8.496 |
| −2.586 | −2.592 | −2.598 | −2.605 | −2.611 | −2.618 | −2.626 | −2.633 | −2.640 | −2.647 | −2.655 | −2.663 | −2.671 | −2.679 | −2.688 | −2.697 | −2.707 |
| −9.854 | −9.825 | −9.797 | −9.769 | −9.741 | −9.714 | −9.687 | −9.661 | −9.635 | −9.609 | −9.584 | −9.559 | −9.535 | −9.512 | −9.489 | −9.468 | −9.447 |
| −2.743 | −2.769 | −2.795 | −2.823 | −2.852 | −2.882 | −2.913 | −2.944 | −2.976 | −3.009 | −3.042 | −3.076 | −3.110 | −3.145 | −3.180 | −3.216 | −3.252 |

| | | | | | | | | | | | | | | | | |
|---|---|---|---|---|---|---|---|---|---|---|---|---|---|---|---|---|
| 4.798 | 4.800 | 4.802 | 4.803 | 4.805 | 4.807 | 4.808 | 4.810 | 4.812 | 4.813 | 4.815 | 4.817 | 4.818 | 4.820 | 4.822 | 4.823 | 4.825 |
| | | | | | | | | | | | | | | | | |
| −8.462 | −8.428 | −8.395 | −8.362 | −8.329 | −8.297 | −8.265 | −8.234 | −8.203 | −8.173 | −8.143 | −8.113 | −8.085 | −8.058 | −8.032 | −8.007 | −7.984 |
| −2.717 | −2.728 | −2.738 | −2.750 | −2.761 | −2.774 | −2.786 | −2.800 | −2.814 | −2.828 | −2.843 | −2.859 | −2.874 | −2.891 | −2.908 | −2.925 | −2.943 |
| | | | | | | | | | | | | | | | | |
| −9.427 | −9.409 | −9.391 | −9.375 | −9.359 | −9.345 | −9.332 | −9.319 | −9.308 | −9.298 | −9.290 | −9.283 | −9.278 | −9.275 | −9.274 | −9.274 | −9.276 |
| −3.289 | −3.327 | −3.364 | −3.403 | −3.441 | −3.480 | −3.519 | −3.558 | −3.596 | −3.634 | −3.671 | −3.707 | −3.743 | −3.778 | −3.812 | −3.846 | −3.878 |

| | | | | | | | | | | | | | | | | |
|---|---|---|---|---|---|---|---|---|---|---|---|---|---|---|---|---|
| 4.827 | 4.828 | 4.830 | 4.832 | 4.833 | 4.835 | 4.837 | 4.838 | 4.840 | 4.842 | 4.843 | 4.845 | 4.847 | 4.848 | 4.850 | 4.852 | 4.853 |
| −7.963 | −7.944 | −7.926 | −7.911 | −7.896 | −7.884 | −7.873 | −7.863 | −7.855 | −7.848 | −7.842 | −7.838 | −7.834 | −7.832 | −7.831 | −7.831 | −7.832 |
| −2.962 | −2.981 | −3.000 | −3.020 | −3.040 | −3.062 | −3.083 | −3.106 | −3.129 | −3.153 | −3.176 | −3.201 | −3.225 | −3.250 | −3.275 | −3.301 | −3.327 |
| −9.279 | −9.284 | −9.291 | −9.299 | −9.309 | −9.320 | −9.332 | −9.345 | −9.360 | −9.376 | −9.393 | −9.411 | −9.430 | −9.450 | −9.470 | −9.491 | −9.513 |
| −3.911 | −3.942 | −3.973 | −4.003 | −4.033 | −4.063 | −4.092 | −4.121 | −4.150 | −4.179 | −4.207 | −4.236 | −4.264 | −4.292 | −4.320 | −4.347 | −4.373 |

| | | | | | | | | | | | | | | | | |
|---|---|---|---|---|---|---|---|---|---|---|---|---|---|---|---|---|
| 4.855 | 4.857 | 4.858 | 4.860 | 4.862 | 4.863 | 4.865 | 4.867 | 4.868 | 4.870 | 4.872 | 4.873 | 4.875 | 4.877 | 4.878 | 4.880 | 4.882 |
| | | | | | | | | | | | | | | | | |
| −7.833 | −7.835 | −7.838 | −7.841 | −7.845 | −7.849 | −7.853 | −7.857 | −7.862 | −7.867 | −7.873 | −7.879 | −7.885 | −7.893 | −7.900 | −7.908 | −7.916 |
| −3.353 | −3.380 | −3.408 | −3.436 | −3.465 | −3.494 | −3.523 | −3.553 | −3.583 | −3.614 | −3.645 | −3.677 | −3.709 | −3.742 | −3.775 | −3.808 | −3.841 |
| | | | | | | | | | | | | | | | | |
| −9.535 | −9.557 | −9.580 | −9.603 | −9.626 | −9.650 | −9.673 | −9.696 | −9.719 | −9.742 | −9.765 | −9.788 | −9.810 | −9.833 | −9.855 | −9.877 | −9.898 |
| −4.399 | −4.424 | −4.449 | −4.474 | −4.498 | −4.521 | −4.543 | −4.565 | −4.586 | −4.607 | −4.627 | −4.647 | −4.665 | −4.684 | −4.702 | −4.720 | −4.737 |

| | | | | | | | | | | | | | | | | |
|---|---|---|---|---|---|---|---|---|---|---|---|---|---|---|---|---|
| 4.883 | 4.885 | 4.887 | 4.888 | 4.890 | 4.892 | 4.893 | 4.895 | 4.897 | 4.898 | 4.900 | 4.902 | 4.903 | 4.905 | 4.907 | 4.908 | 4.910 |
| −7.925 | −7.934 | −7.943 | −7.953 | −7.963 | −7.974 | −7.986 | −7.998 | −8.011 | −8.024 | −8.038 | −8.053 | −8.068 | −8.084 | −8.100 | −8.117 | −8.134 |
| −3.874 | −3.908 | −3.941 | −3.974 | −4.007 | −4.040 | −4.073 | −4.106 | −4.139 | −4.172 | −4.205 | −4.238 | −4.271 | −4.303 | −4.336 | −4.369 | −4.401 |
| −9.920 | −9.941 | −9.962 | −9.983 | −10.003 | −10.023 | −10.041 | −10.059 | −10.076 | −10.092 | −10.107 | −10.121 | −10.134 | −10.147 | −10.158 | −10.170 | −10.181 |
| −4.754 | −4.771 | −4.787 | −4.802 | −4.817 | −4.832 | −4.847 | −4.861 | −4.876 | −4.890 | −4.905 | −4.920 | −4.935 | −4.950 | −4.966 | −4.982 | −4.999 |

| | | | | | | | | | | | | | | | | |
|---|---|---|---|---|---|---|---|---|---|---|---|---|---|---|---|---|
| 4.912 | 4.913 | 4.915 | 4.917 | 4.918 | 4.920 | 4.922 | 4.923 | 4.925 | 4.927 | 4.928 | 4.930 | 4.932 | 4.933 | 4.935 | 4.937 | 4.938 |
| −8.152 | −8.170 | −8.188 | −8.206 | −8.225 | −8.243 | −8.261 | −8.279 | −8.296 | −8.314 | −8.330 | −8.347 | −8.363 | −8.379 | −8.394 | −8.409 | −8.424 |
| −4.433 | −4.465 | −4.497 | −4.529 | −4.561 | −4.593 | −4.624 | −4.656 | −4.687 | −4.719 | −4.750 | −4.782 | −4.813 | −4.845 | −4.877 | −4.909 | −4.941 |
| −10.192 | −10.202 | −10.211 | −10.219 | −10.227 | −10.234 | −10.240 | −10.245 | −10.250 | −10.253 | −10.256 | −10.258 | −10.260 | −10.260 | −10.260 | −10.259 | −10.256 |
| −5.015 | −5.033 | −5.051 | −5.070 | −5.089 | −5.109 | −5.129 | −5.150 | −5.172 | −5.194 | −5.217 | −5.240 | −5.264 | −5.289 | −5.314 | −5.340 | −5.367 |

| | | | | | | | | | | | | | | | | |
|---|---|---|---|---|---|---|---|---|---|---|---|---|---|---|---|---|
| 4.940 | 4.942 | 4.943 | 4.945 | 4.947 | 4.948 | 4.950 | 4.952 | 4.953 | 4.955 | 4.957 | 4.958 | 4.960 | 4.962 | 4.963 | 4.965 | 4.967 |
| | | | | | | | | | | | | | | | | |
| −8.437 | −8.450 | −8.462 | −8.473 | −8.484 | −8.493 | −8.501 | −8.507 | −8.512 | −8.516 | −8.518 | −8.518 | −8.517 | −8.514 | −8.510 | −8.504 | −8.498 |
| −4.973 | −5.006 | −5.038 | −5.072 | −5.105 | −5.139 | −5.173 | −5.207 | −5.241 | −5.275 | −5.309 | −5.343 | −5.377 | −5.411 | −5.444 | −5.477 | −5.510 |
| | | | | | | | | | | | | | | | | |
| −10.253 | −10.248 | −10.243 | −10.237 | −10.230 | −10.222 | −10.213 | −10.203 | −10.192 | −10.180 | −10.166 | −10.151 | −10.135 | −10.117 | −10.098 | −10.078 | −10.057 |
| −5.395 | −5.422 | −5.451 | −5.480 | −5.509 | −5.538 | −5.567 | −5.596 | −5.625 | −5.653 | −5.682 | −5.710 | −5.738 | −5.766 | −5.793 | −5.820 | −5.847 |

| | | | | | | | | | | | | | | | | |
|---|---|---|---|---|---|---|---|---|---|---|---|---|---|---|---|---|
| 4.968 | 4.970 | 4.972 | 4.973 | 4.975 | 4.977 | 4.978 | 4.980 | 4.982 | 4.983 | 4.985 | 4.987 | 4.988 | 4.990 | 4.992 | 4.993 | 4.995 |
| −8.490 | −8.481 | −8.472 | −8.461 | −8.450 | −8.438 | −8.425 | −8.411 | −8.397 | −8.382 | −8.366 | −8.348 | −8.330 | −8.311 | −8.291 | −8.270 | −8.249 |
| −5.542 | −5.573 | −5.603 | −5.633 | −5.662 | −5.690 | −5.718 | −5.745 | −5.771 | −5.796 | −5.821 | −5.845 | −5.868 | −5.890 | −5.911 | −5.932 | −5.952 |
| −10.034 | −10.011 | −9.988 | −9.964 | −9.940 | −9.916 | −9.891 | −9.866 | −9.841 | −9.815 | −9.789 | −9.762 | −9.734 | −9.706 | −9.678 | −9.648 | −9.618 |
| −5.874 | −5.900 | −5.926 | −5.951 | −5.975 | −5.999 | −6.022 | −6.045 | −6.067 | −6.088 | −6.109 | −6.129 | −6.148 | −6.166 | −6.184 | −6.201 | −6.217 |

| | | | | | | | | | | | | | | | | |
|---|---|---|---|---|---|---|---|---|---|---|---|---|---|---|---|---|
| 4.997 | 4.998 | 5.000 | 5.002 | 5.003 | 5.005 | 5.007 | 5.008 | 5.010 | 5.012 | 5.013 | 5.015 | 5.017 | 5.018 | 5.020 | 5.022 | 5.023 |
| | | | | | | | | | | | | | | | | |
| −8.227 | −8.204 | −8.181 | −8.158 | −8.134 | −8.110 | −8.085 | −8.061 | −8.035 | −8.009 | −7.983 | −7.957 | −7.931 | −7.905 | −7.878 | −7.852 | −7.826 |
| −5.971 | −5.989 | −6.007 | −6.024 | −6.040 | −6.056 | −6.071 | −6.085 | −6.098 | −6.110 | −6.122 | −6.133 | −6.144 | −6.153 | −6.162 | −6.169 | −6.176 |
| | | | | | | | | | | | | | | | | |
| −9.587 | −9.556 | −9.524 | −9.490 | −9.456 | −9.420 | −9.383 | −9.346 | −9.307 | −9.268 | −9.228 | −9.188 | −9.148 | −9.108 | −9.067 | −9.027 | −8.987 |
| −6.232 | −6.247 | −6.261 | −6.274 | −6.287 | −6.299 | −6.310 | −6.321 | −6.330 | −6.339 | −6.347 | −6.354 | −6.360 | −6.364 | −6.368 | −6.371 | −6.372 |

| | | | | | | | | | | | | | | | | |
|---|---|---|---|---|---|---|---|---|---|---|---|---|---|---|---|---|
| 5.025 | 5.027 | 5.028 | 5.030 | 5.032 | 5.033 | 5.035 | 5.037 | 5.038 | 5.040 | 5.041 | 5.043 | 5.045 | 5.046 | 5.048 | 5.050 | 5.051 |
| −7.801 | −7.776 | −7.751 | −7.728 | −7.705 | −7.683 | −7.663 | −7.644 | −7.625 | −7.609 | −7.593 | −7.580 | −7.567 | −7.556 | −7.546 | −7.538 | −7.530 |
| −6.182 | −6.187 | −6.191 | −6.194 | −6.197 | −6.199 | −6.200 | −6.200 | −6.199 | −6.198 | −6.195 | −6.192 | −6.188 | −6.183 | −6.177 | −6.171 | −6.164 |
| −8.948 | −8.909 | −8.870 | −8.832 | −8.795 | −8.759 | −8.723 | −8.689 | −8.656 | −8.623 | −8.592 | −8.562 | −8.533 | −8.505 | −8.479 | −8.453 | −8.429 |
| −6.371 | −6.370 | −6.366 | −6.362 | −6.356 | −6.350 | −6.342 | −6.333 | −6.323 | −6.312 | −6.300 | −6.288 | −6.275 | −6.261 | −6.246 | −6.231 | −6.216 |

| | | | | | | | | | | | | | | | | |
|---|---|---|---|---|---|---|---|---|---|---|---|---|---|---|---|---|
| 5.053 | 5.055 | 5.056 | 5.058 | 5.060 | 5.061 | 5.063 | 5.065 | 5.066 | 5.068 | 5.070 | 5.071 | 5.073 | 5.075 | 5.076 | 5.078 | 5.080 |
| −7.524 | −7.519 | −7.515 | −7.511 | −7.508 | −7.506 | −7.505 | −7.503 | −7.503 | −7.504 | −7.505 | −7.507 | −7.510 | −7.514 | −7.519 | −7.524 | −7.531 |
| −6.156 | −6.148 | −6.140 | −6.131 | −6.122 | −6.113 | −6.103 | −6.093 | −6.083 | −6.072 | −6.061 | −6.050 | −6.038 | −6.026 | −6.013 | −6.001 | −5.987 |
| −8.406 | −8.384 | −8.364 | −8.344 | −8.325 | −8.308 | −8.292 | −8.277 | −8.264 | −8.252 | −8.241 | −8.232 | −8.224 | −8.217 | −8.211 | −8.207 | −8.203 |
| −6.200 | −6.184 | −6.169 | −6.153 | −6.137 | −6.122 | −6.107 | −6.093 | −6.079 | −6.065 | −6.051 | −6.038 | −6.025 | −6.012 | −5.999 | −5.987 | −5.974 |

| | | | | | | | | | | | | | | | | |
|---|---|---|---|---|---|---|---|---|---|---|---|---|---|---|---|---|
| 5.081 | 5.083 | 5.085 | 5.086 | 5.088 | 5.090 | 5.091 | 5.093 | 5.095 | 5.096 | 5.098 | 5.100 | 5.101 | 5.103 | 5.105 | 5.106 | 5.108 |
| | | | | | | | | | | | | | | | | |
| −7.537 | −7.544 | −7.552 | −7.560 | −7.569 | −7.579 | −7.589 | −7.599 | −7.611 | −7.623 | −7.635 | −7.648 | −7.660 | −7.672 | −7.685 | −7.696 | −7.707 |
| −5.974 | −5.961 | −5.947 | −5.933 | −5.920 | −5.907 | −5.894 | −5.881 | −5.869 | −5.857 | −5.846 | −5.836 | −5.827 | −5.819 | −5.813 | −5.808 | −5.804 |
| | | | | | | | | | | | | | | | | |
| −8.201 | −8.200 | −8.200 | −8.200 | −8.202 | −8.205 | −8.208 | −8.212 | −8.217 | −8.222 | −8.228 | −8.234 | −8.241 | −8.248 | −8.255 | −8.262 | −8.270 |
| −5.961 | −5.949 | −5.937 | −5.925 | −5.914 | −5.904 | −5.895 | −5.887 | −5.879 | −5.873 | −5.868 | −5.864 | −5.862 | −5.861 | −5.862 | −5.864 | −5.868 |

| | | | | | | | | | | | | | | | | |
|---|---|---|---|---|---|---|---|---|---|---|---|---|---|---|---|---|
| 5.110 | 5.111 | 5.113 | 5.115 | 5.116 | 5.118 | 5.120 | 5.121 | 5.123 | 5.125 | 5.126 | 5.128 | 5.130 | 5.131 | 5.133 | 5.135 | 5.136 |
| −7.718 | −7.728 | −7.738 | −7.747 | −7.756 | −7.764 | −7.771 | −7.778 | −7.783 | −7.787 | −7.790 | −7.791 | −7.791 | −7.790 | −7.787 | −7.782 | −7.776 |
| −5.802 | −5.801 | −5.802 | −5.804 | −5.808 | −5.813 | −5.820 | −5.827 | −5.836 | −5.845 | −5.856 | −5.868 | −5.881 | −5.895 | −5.910 | −5.927 | −5.944 |
| −8.278 | −8.286 | −8.294 | −8.301 | −8.307 | −8.313 | −8.318 | −8.322 | −8.324 | −8.325 | −8.325 | −8.323 | −8.320 | −8.314 | −8.306 | −8.296 | −8.284 |
| −5.874 | −5.881 | −5.891 | −5.901 | −5.914 | −5.928 | −5.944 | −5.962 | −5.982 | −6.003 | −6.026 | −6.050 | −6.076 | −6.104 | −6.134 | −6.165 | −6.197 |

| | | | | | | | | | | | | | | | | |
|---|---|---|---|---|---|---|---|---|---|---|---|---|---|---|---|---|
| 5.138 | 5.140 | 5.141 | 5.143 | 5.145 | 5.146 | 5.148 | 5.150 | 5.151 | 5.153 | 5.155 | 5.156 | 5.158 | 5.160 | 5.161 | 5.163 | 5.165 |
| | | | | | | | | | | | | | | | | |
| −7.768 | −7.758 | −7.746 | −7.733 | −7.717 | −7.701 | −7.682 | −7.662 | −7.640 | −7.617 | −7.592 | −7.566 | −7.538 | −7.509 | −7.478 | −7.445 | −7.411 |
| −5.963 | −5.983 | −6.004 | −6.026 | −6.050 | −6.074 | −6.098 | −6.124 | −6.150 | −6.176 | −6.203 | −6.231 | −6.259 | −6.287 | −6.315 | −6.344 | −6.373 |
| | | | | | | | | | | | | | | | | |
| −8.270 | −8.253 | −8.234 | −8.212 | −8.189 | −8.164 | −8.137 | −8.107 | −8.076 | −8.043 | −8.008 | −7.971 | −7.933 | −7.894 | −7.853 | −7.811 | −7.768 |
| −6.231 | −6.266 | −6.303 | −6.340 | −6.378 | −6.417 | −6.457 | −6.498 | −6.540 | −6.582 | −6.624 | −6.668 | −6.712 | −6.756 | −6.801 | −6.847 | −6.893 |

| | | | | | | | | | | | | | | | | |
|---|---|---|---|---|---|---|---|---|---|---|---|---|---|---|---|---|
| 5.166 | 5.168 | 5.170 | 5.171 | 5.173 | 5.175 | 5.176 | 5.178 | 5.180 | 5.181 | 5.183 | 5.185 | 5.186 | 5.188 | 5.190 | 5.191 | 5.193 |
| −7.376 | −7.340 | −7.303 | −7.265 | −7.225 | −7.185 | −7.144 | −7.102 | −7.059 | −7.015 | −6.971 | −6.926 | −6.881 | −6.835 | −6.789 | −6.743 | −6.697 |
| −6.401 | −6.430 | −6.459 | −6.488 | −6.516 | −6.545 | −6.573 | −6.602 | −6.630 | −6.658 | −6.686 | −6.714 | −6.741 | −6.768 | −6.794 | −6.820 | −6.844 |
| −7.723 | −7.679 | −7.633 | −7.587 | −7.541 | −7.494 | −7.447 | −7.399 | −7.352 | −7.305 | −7.259 | −7.213 | −7.167 | −7.122 | −7.077 | −7.032 | −6.989 |
| −6.939 | −6.985 | −7.032 | −7.078 | −7.125 | −7.171 | −7.217 | −7.262 | −7.307 | −7.350 | −7.393 | −7.436 | −7.477 | −7.517 | −7.556 | −7.593 | −7.630 |

| | | | | | | | | | | | | | | | | |
|---|---|---|---|---|---|---|---|---|---|---|---|---|---|---|---|---|
| 5.195 | 5.196 | 5.198 | 5.200 | 5.201 | 5.203 | 5.205 | 5.206 | 5.208 | 5.210 | 5.211 | 5.213 | 5.215 | 5.216 | 5.218 | 5.220 | 5.221 |
| | | | | | | | | | | | | | | | | |
| −6.650 | −6.604 | −6.558 | −6.512 | −6.466 | −6.422 | −6.378 | −6.335 | −6.293 | −6.252 | −6.212 | −6.174 | −6.137 | −6.102 | −6.068 | −6.035 | −6.004 |
| −6.869 | −6.892 | −6.915 | −6.937 | −6.959 | −6.980 | −7.000 | −7.019 | −7.038 | −7.056 | −7.074 | −7.091 | −7.108 | −7.123 | −7.138 | −7.152 | −7.166 |
| | | | | | | | | | | | | | | | | |
| −6.945 | −6.902 | −6.859 | −6.818 | −6.776 | −6.736 | −6.696 | −6.658 | −6.620 | −6.583 | −6.547 | −6.513 | −6.479 | −6.447 | −6.416 | −6.386 | −6.357 |
| −7.665 | −7.699 | −7.731 | −7.761 | −7.790 | −7.817 | −7.843 | −7.867 | −7.890 | −7.911 | −7.931 | −7.950 | −7.967 | −7.983 | −7.997 | −8.009 | −8.021 |

| | | | | | | | | | | | | | | | | |
|---|---|---|---|---|---|---|---|---|---|---|---|---|---|---|---|---|
| 5.223 | 5.225 | 5.226 | 5.228 | 5.230 | 5.231 | 5.233 | 5.235 | 5.236 | 5.238 | 5.240 | 5.241 | 5.243 | 5.245 | 5.246 | 5.248 | 5.250 |
| | | | | | | | | | | | | | | | | |
| −5.976 | −5.949 | −5.924 | −5.900 | −5.878 | −5.858 | −5.840 | −5.824 | −5.809 | −5.797 | −5.786 | −5.778 | −5.771 | −5.767 | −5.765 | −5.765 | −5.767 |
| −7.179 | −7.191 | −7.202 | −7.213 | −7.222 | −7.231 | −7.238 | −7.245 | −7.250 | −7.255 | −7.259 | −7.262 | −7.263 | −7.264 | −7.264 | −7.263 | −7.261 |
| | | | | | | | | | | | | | | | | |
| −6.330 | −6.304 | −6.280 | −6.257 | −6.235 | −6.215 | −6.196 | −6.178 | −6.162 | −6.147 | −6.133 | −6.122 | −6.111 | −6.102 | −6.095 | −6.089 | −6.085 |
| −8.031 | −8.039 | −8.046 | −8.052 | −8.057 | −8.060 | −8.062 | −8.064 | −8.064 | −8.063 | −8.062 | −8.059 | −8.055 | −8.050 | −8.044 | −8.038 | −8.030 |

| | | | | | | | | | | | | | | | | |
|---|---|---|---|---|---|---|---|---|---|---|---|---|---|---|---|---|
| 5.251 | 5.253 | 5.255 | 5.256 | 5.258 | 5.260 | 5.261 | 5.263 | 5.265 | 5.266 | 5.268 | 5.270 | 5.271 | 5.273 | 5.275 | 5.276 | 5.278 |
| −5.771 | −5.776 | −5.784 | −5.793 | −5.805 | −5.817 | −5.832 | −5.847 | −5.865 | −5.883 | −5.903 | −5.925 | −5.947 | −5.971 | −5.996 | −6.022 | −6.049 |
| −7.258 | −7.256 | −7.252 | −7.249 | −7.245 | −7.242 | −7.239 | −7.236 | −7.233 | −7.230 | −7.227 | −7.225 | −7.223 | −7.222 | −7.220 | −7.220 | −7.220 |
| −6.082 | −6.081 | −6.082 | −6.084 | −6.088 | −6.094 | −6.102 | −6.111 | −6.123 | −6.137 | −6.153 | −6.170 | −6.190 | −6.211 | −6.234 | −6.259 | −6.286 |
| −8.022 | −8.013 | −8.003 | −7.992 | −7.981 | −7.970 | −7.957 | −7.945 | −7.931 | −7.918 | −7.904 | −7.890 | −7.875 | −7.861 | −7.846 | −7.832 | −7.818 |

| | | | | | | | | | | | | | | | | |
|---|---|---|---|---|---|---|---|---|---|---|---|---|---|---|---|---|
| 5.280 | 5.281 | 5.283 | 5.285 | 5.286 | 5.288 | 5.290 | 5.291 | 5.293 | 5.295 | 5.296 | 5.298 | 5.300 | 5.301 | 5.303 | 5.305 | 5.306 |
| −6.077 | −6.106 | −6.135 | −6.165 | −6.196 | −6.228 | −6.260 | −6.293 | −6.327 | −6.361 | −6.396 | −6.432 | −6.468 | −6.504 | −6.542 | −6.579 | −6.617 |
| −7.220 | −7.221 | −7.222 | −7.225 | −7.227 | −7.231 | −7.235 | −7.240 | −7.245 | −7.250 | −7.256 | −7.263 | −7.270 | −7.277 | −7.285 | −7.293 | −7.301 |
| −6.314 | −6.344 | −6.375 | −6.408 | −6.443 | −6.478 | −6.514 | −6.551 | −6.589 | −6.627 | −6.667 | −6.706 | −6.746 | −6.787 | −6.827 | −6.867 | −6.908 |
| −7.804 | −7.790 | −7.777 | −7.763 | −7.751 | −7.739 | −7.727 | −7.717 | −7.707 | −7.697 | −7.689 | −7.681 | −7.673 | −7.666 | −7.658 | −7.651 | −7.644 |

| | | | | | | | | | | | | | | | | |
|---|---|---|---|---|---|---|---|---|---|---|---|---|---|---|---|---|
| 5.308 | 5.310 | 5.311 | 5.313 | 5.315 | 5.316 | 5.318 | 5.320 | 5.321 | 5.323 | 5.325 | 5.326 | 5.328 | 5.330 | 5.331 | 5.333 | 5.335 |
| | | | | | | | | | | | | | | | | |
| −6.655 | −6.693 | −6.731 | −6.769 | −6.806 | −6.843 | −6.879 | −6.915 | −6.950 | −6.985 | −7.018 | −7.052 | −7.084 | −7.116 | −7.147 | −7.177 | −7.206 |
| −7.310 | −7.318 | −7.326 | −7.334 | −7.343 | −7.350 | −7.357 | −7.364 | −7.370 | −7.374 | −7.379 | −7.382 | −7.384 | −7.385 | −7.385 | −7.384 | −7.381 |
| | | | | | | | | | | | | | | | | |
| −6.948 | −6.988 | −7.028 | −7.068 | −7.107 | −7.146 | −7.184 | −7.221 | −7.258 | −7.294 | −7.329 | −7.364 | −7.397 | −7.430 | −7.462 | −7.493 | −7.522 |
| −7.637 | −7.630 | −7.623 | −7.615 | −7.608 | −7.600 | −7.592 | −7.583 | −7.574 | −7.564 | −7.554 | −7.542 | −7.529 | −7.515 | −7.499 | −7.482 | −7.464 |

| | | | | | | | | | | | | | | | | |
|---|---|---|---|---|---|---|---|---|---|---|---|---|---|---|---|---|
| 5.336 | 5.338 | 5.340 | 5.341 | 5.343 | 5.345 | 5.346 | 5.348 | 5.350 | 5.351 | 5.353 | 5.355 | 5.356 | 5.358 | 5.360 | 5.361 | 5.363 |
| | | | | | | | | | | | | | | | | |
| −7.234 | −7.261 | −7.287 | −7.312 | −7.335 | −7.357 | −7.378 | −7.397 | −7.414 | −7.430 | −7.443 | −7.454 | −7.462 | −7.469 | −7.473 | −7.476 | −7.475 |
| −7.377 | −7.372 | −7.365 | −7.356 | −7.347 | −7.335 | −7.323 | −7.308 | −7.293 | −7.275 | −7.257 | −7.237 | −7.217 | −7.194 | −7.171 | −7.145 | −7.119 |
| | | | | | | | | | | | | | | | | |
| −7.550 | −7.576 | −7.601 | −7.624 | −7.644 | −7.663 | −7.679 | −7.694 | −7.705 | −7.715 | −7.722 | −7.727 | −7.729 | −7.728 | −7.725 | −7.719 | −7.710 |
| −7.445 | −7.425 | −7.403 | −7.381 | −7.357 | −7.332 | −7.306 | −7.278 | −7.250 | −7.219 | −7.189 | −7.156 | −7.124 | −7.089 | −7.055 | −7.019 | −6.983 |

| | | | | | | | | | | | | | | | | |
|---|---|---|---|---|---|---|---|---|---|---|---|---|---|---|---|---|
| 5.365 | 5.366 | 5.368 | 5.369 | 5.371 | 5.373 | 5.374 | 5.376 | 5.378 | 5.379 | 5.381 | 5.383 | 5.384 | 5.386 | 5.388 | 5.389 | 5.391 |
| −7.474 | −7.470 | −7.464 | −7.456 | −7.447 | −7.435 | −7.422 | −7.406 | −7.389 | −7.369 | −7.349 | −7.325 | −7.301 | −7.275 | −7.247 | −7.217 | −7.186 |
| −7.091 | −7.062 | −7.032 | −7.000 | −6.966 | −6.932 | −6.895 | −6.858 | −6.819 | −6.779 | −6.737 | −6.695 | −6.651 | −6.606 | −6.560 | −6.513 | −6.465 |
| −7.698 | −7.683 | −7.666 | −7.646 | −7.624 | −7.598 | −7.571 | −7.541 | −7.509 | −7.475 | −7.439 | −7.401 | −7.361 | −7.319 | −7.275 | −7.229 | −7.182 |
| −6.945 | −6.908 | −6.869 | −6.830 | −6.790 | −6.750 | −6.709 | −6.669 | −6.627 | −6.585 | −6.543 | −6.501 | −6.458 | −6.416 | −6.374 | −6.332 | −6.291 |

| | | | | | | | | | | | | | | | | |
|---|---|---|---|---|---|---|---|---|---|---|---|---|---|---|---|---|
| 5.393 | 5.394 | 5.396 | 5.398 | 5.399 | 5.401 | 5.403 | 5.404 | 5.406 | 5.408 | 5.409 | 5.411 | 5.413 | 5.414 | 5.416 | 5.418 | 5.419 |
| −7.153 | −7.118 | −7.081 | −7.044 | −7.005 | −6.964 | −6.924 | −6.883 | −6.842 | −6.800 | −6.759 | −6.717 | −6.676 | −6.635 | −6.594 | −6.554 | −6.515 |
| −6.417 | −6.367 | −6.317 | −6.266 | −6.215 | −6.164 | −6.112 | −6.061 | −6.010 | −5.959 | −5.909 | −5.859 | −5.810 | −5.761 | −5.714 | −5.667 | −5.622 |
| −7.133 | −7.083 | −7.032 | −6.980 | −6.928 | −6.876 | −6.823 | −6.771 | −6.718 | −6.666 | −6.615 | −6.563 | −6.513 | −6.464 | −6.416 | −6.368 | −6.322 |
| −6.250 | −6.209 | −6.168 | −6.128 | −6.088 | −6.049 | −6.010 | −5.971 | −5.932 | −5.894 | −5.856 | −5.819 | −5.781 | −5.744 | −5.708 | −5.673 | −5.638 |

| | | | | | | | | | | | | | | | | |
|---|---|---|---|---|---|---|---|---|---|---|---|---|---|---|---|---|
| 5.421 | 5.423 | 5.424 | 5.426 | 5.428 | 5.429 | 5.431 | 5.433 | 5.434 | 5.436 | 5.438 | 5.439 | 5.441 | 5.443 | 5.444 | 5.446 | 5.448 |
| | | | | | | | | | | | | | | | | |
| −6.475 | −6.438 | −6.400 | −6.364 | −6.328 | −6.294 | −6.260 | −6.228 | −6.196 | −6.166 | −6.136 | −6.108 | −6.081 | −6.056 | −6.032 | −6.009 | −5.988 |
| −5.578 | −5.535 | −5.493 | −5.453 | −5.414 | −5.377 | −5.341 | −5.307 | −5.274 | −5.243 | −5.214 | −5.186 | −5.160 | −5.135 | −5.112 | −5.091 | −5.071 |
| | | | | | | | | | | | | | | | | |
| −6.278 | −6.235 | −6.193 | −6.154 | −6.115 | −6.078 | −6.043 | −6.009 | −5.976 | −5.945 | −5.916 | −5.888 | −5.863 | −5.838 | −5.816 | −5.796 | −5.778 |
| −5.603 | −5.569 | −5.536 | −5.504 | −5.472 | −5.442 | −5.413 | −5.386 | −5.359 | −5.334 | −5.310 | −5.287 | −5.265 | −5.244 | −5.224 | −5.205 | −5.188 |

| | | | | | | | | | | | | | | | | |
|---|---|---|---|---|---|---|---|---|---|---|---|---|---|---|---|---|
| 5.449 | 5.451 | 5.453 | 5.454 | 5.456 | 5.458 | 5.459 | 5.461 | 5.463 | 5.464 | 5.466 | 5.468 | 5.469 | 5.471 | 5.473 | 5.474 | 5.476 |
| −5.968 | −5.949 | −5.932 | −5.917 | −5.903 | −5.891 | −5.880 | −5.871 | −5.863 | −5.857 | −5.852 | −5.848 | −5.845 | −5.843 | −5.842 | −5.841 | −5.841 |
| −5.052 | −5.035 | −5.019 | −5.004 | −4.991 | −4.978 | −4.967 | −4.957 | −4.948 | −4.940 | −4.933 | −4.926 | −4.921 | −4.915 | −4.911 | −4.907 | −4.904 |
| −5.762 | −5.749 | −5.738 | −5.730 | −5.724 | −5.721 | −5.720 | −5.721 | −5.724 | −5.729 | −5.735 | −5.744 | −5.754 | −5.765 | −5.777 | −5.790 | −5.804 |
| −5.171 | −5.155 | −5.141 | −5.128 | −5.116 | −5.105 | −5.096 | −5.087 | −5.079 | −5.072 | −5.066 | −5.061 | −5.057 | −5.054 | −5.052 | −5.050 | −5.049 |

| | | | | | | | | | | | | | | | | |
|---|---|---|---|---|---|---|---|---|---|---|---|---|---|---|---|---|
| 5.478 | 5.479 | 5.481 | 5.483 | 5.484 | 5.486 | 5.488 | 5.489 | 5.491 | 5.493 | 5.494 | 5.496 | 5.498 | 5.499 | 5.501 | 5.503 | 5.504 |
| | | | | | | | | | | | | | | | | |
| −5.841 | −5.841 | −5.841 | −5.841 | −5.841 | −5.841 | −5.841 | −5.839 | −5.837 | −5.835 | −5.831 | −5.826 | −5.820 | −5.812 | −5.803 | −5.793 | −5.780 |
| −4.901 | −4.899 | −4.896 | −4.894 | −4.892 | −4.890 | −4.887 | −4.885 | −4.882 | −4.879 | −4.876 | −4.871 | −4.866 | −4.860 | −4.853 | −4.845 | −4.836 |
| | | | | | | | | | | | | | | | | |
| −5.819 | −5.834 | −5.850 | −5.867 | −5.884 | −5.901 | −5.918 | −5.936 | −5.953 | −5.970 | −5.986 | −6.002 | −6.017 | −6.031 | −6.043 | −6.055 | −6.065 |
| −5.049 | −5.049 | −5.050 | −5.052 | −5.054 | −5.056 | −5.059 | −5.063 | −5.067 | −5.071 | −5.075 | −5.080 | −5.084 | −5.088 | −5.092 | −5.096 | −5.099 |

| | | | | | | | | | | | | | | | | |
|---|---|---|---|---|---|---|---|---|---|---|---|---|---|---|---|---|
| 5.506 | 5.508 | 5.509 | 5.511 | 5.513 | 5.514 | 5.516 | 5.518 | 5.519 | 5.521 | 5.523 | 5.524 | 5.526 | 5.528 | 5.529 | 5.531 | 5.533 |
| | | | | | | | | | | | | | | | | |
| −5.766 | −5.749 | −5.731 | −5.709 | −5.685 | −5.658 | −5.629 | −5.597 | −5.563 | −5.526 | −5.486 | −5.444 | −5.400 | −5.354 | −5.305 | −5.255 | −5.202 |
| −4.825 | −4.814 | −4.801 | −4.787 | −4.772 | −4.756 | −4.738 | −4.720 | −4.700 | −4.678 | −4.655 | −4.630 | −4.603 | −4.575 | −4.544 | −4.512 | −4.478 |
| | | | | | | | | | | | | | | | | |
| −6.073 | −6.079 | −6.083 | −6.084 | −6.083 | −6.079 | −6.071 | −6.062 | −6.048 | −6.032 | −6.012 | −5.990 | −5.963 | −5.935 | −5.904 | −5.870 | −5.834 |
| −5.101 | −5.102 | −5.103 | −5.103 | −5.102 | −5.100 | −5.098 | −5.095 | −5.092 | −5.087 | −5.082 | −5.077 | −5.071 | −5.064 | −5.056 | −5.047 | −5.037 |

| | | | | | | | | | | | | | | | | |
|---|---|---|---|---|---|---|---|---|---|---|---|---|---|---|---|---|
| 5.534 | 5.536 | 5.538 | 5.539 | 5.541 | 5.543 | 5.544 | 5.546 | 5.548 | 5.549 | 5.551 | 5.553 | 5.554 | 5.556 | 5.558 | 5.559 | 5.561 |
| | | | | | | | | | | | | | | | | |
| −5.148 | −5.092 | −5.034 | −4.975 | −4.915 | −4.854 | −4.792 | −4.728 | −4.664 | −4.599 | −4.534 | −4.469 | −4.404 | −4.339 | −4.274 | −4.209 | −4.145 |
| −4.442 | −4.404 | −4.364 | −4.322 | −4.278 | −4.231 | −4.183 | −4.132 | −4.080 | −4.025 | −3.969 | −3.911 | −3.851 | −3.789 | −3.726 | −3.661 | −3.595 |
| | | | | | | | | | | | | | | | | |
| −5.797 | −5.757 | −5.717 | −5.674 | −5.631 | −5.586 | −5.541 | −5.494 | −5.447 | −5.400 | −5.352 | −5.304 | −5.255 | −5.207 | −5.158 | −5.109 | −5.060 |
| −5.026 | −5.014 | −5.001 | −4.987 | −4.971 | −4.955 | −4.937 | −4.918 | −4.898 | −4.877 | −4.853 | −4.829 | −4.802 | −4.774 | −4.745 | −4.714 | −4.681 |

| | | | | | | | | | | | | | | | | |
|---|---|---|---|---|---|---|---|---|---|---|---|---|---|---|---|---|
| 5.563 | 5.564 | 5.566 | 5.568 | 5.569 | 5.571 | 5.573 | 5.574 | 5.576 | 5.578 | 5.579 | 5.581 | 5.583 | 5.584 | 5.586 | 5.588 | 5.589 |
| | | | | | | | | | | | | | | | | |
| −4.082 | −4.019 | −3.958 | −3.897 | −3.838 | −3.780 | −3.724 | −3.668 | −3.615 | −3.564 | −3.515 | −3.467 | −3.422 | −3.378 | −3.337 | −3.297 | −3.260 |
| −3.528 | −3.459 | −3.390 | −3.319 | −3.247 | −3.174 | −3.100 | −3.026 | −2.951 | −2.875 | −2.799 | −2.723 | −2.647 | −2.571 | −2.494 | −2.418 | −2.343 |
| | | | | | | | | | | | | | | | | |
| −5.012 | −4.963 | −4.915 | −4.867 | −4.820 | −4.773 | −4.727 | −4.682 | −4.639 | −4.596 | −4.555 | −4.515 | −4.476 | −4.439 | −4.404 | −4.371 | −4.339 |
| −4.647 | −4.612 | −4.575 | −4.537 | −4.497 | −4.457 | −4.415 | −4.372 | −4.327 | −4.282 | −4.235 | −4.188 | −4.140 | −4.092 | −4.043 | −3.993 | −3.943 |

| | | | | | | | | | | | | | | | | |
|---|---|---|---|---|---|---|---|---|---|---|---|---|---|---|---|---|
| 5.591 | 5.593 | 5.594 | 5.596 | 5.598 | 5.599 | 5.601 | 5.603 | 5.604 | 5.606 | 5.608 | 5.609 | 5.611 | 5.613 | 5.614 | 5.616 | 5.618 |
| | | | | | | | | | | | | | | | | |
| −3.224 | −3.192 | −3.161 | −3.133 | −3.108 | −3.084 | −3.065 | −3.048 | −3.035 | −3.026 | −3.021 | −3.019 | −3.023 | −3.029 | −3.041 | −3.056 | −3.076 |
| −2.268 | −2.194 | −2.122 | −2.050 | −1.981 | −1.913 | −1.847 | −1.783 | −1.722 | −1.663 | −1.607 | −1.554 | −1.504 | −1.458 | −1.414 | −1.375 | −1.338 |
| | | | | | | | | | | | | | | | | |
| −4.310 | −4.283 | −4.258 | −4.235 | −4.214 | −4.196 | −4.181 | −4.167 | −4.157 | −4.149 | −4.145 | −4.143 | −4.145 | −4.150 | −4.159 | −4.170 | −4.186 |
| −3.893 | −3.843 | −3.794 | −3.745 | −3.697 | −3.649 | −3.603 | −3.558 | −3.515 | −3.473 | −3.433 | −3.396 | −3.361 | −3.329 | −3.299 | −3.272 | −3.248 |

| | | | | | | | | | | | | | | | | |
|---|---|---|---|---|---|---|---|---|---|---|---|---|---|---|---|---|
| 5.619 | 5.621 | 5.623 | 5.624 | 5.626 | 5.628 | 5.629 | 5.631 | 5.633 | 5.634 | 5.636 | 5.638 | 5.639 | 5.641 | 5.643 | 5.644 | 5.646 |
| −3.099 | −3.128 | −3.160 | −3.197 | −3.238 | −3.282 | −3.331 | −3.383 | −3.438 | −3.498 | −3.561 | −3.627 | −3.697 | −3.771 | −3.847 | −3.927 | −4.010 |
| −1.307 | −1.278 | −1.255 | −1.235 | −1.220 | −1.209 | −1.203 | −1.201 | −1.204 | −1.211 | −1.223 | −1.239 | −1.261 | −1.286 | −1.316 | −1.351 | −1.390 |
| −4.204 | −4.226 | −4.251 | −4.281 | −4.313 | −4.349 | −4.388 | −4.431 | −4.477 | −4.526 | −4.578 | −4.634 | −4.693 | −4.755 | −4.820 | −4.889 | −4.960 |
| −3.228 | −3.210 | −3.195 | −3.183 | −3.175 | −3.170 | −3.168 | −3.169 | −3.175 | −3.183 | −3.195 | −3.210 | −3.228 | −3.250 | −3.275 | −3.304 | −3.336 |

| | | | | | | | | | | | | | | | | |
|---|---|---|---|---|---|---|---|---|---|---|---|---|---|---|---|---|
| 5.648 | 5.649 | 5.651 | 5.653 | 5.654 | 5.656 | 5.658 | 5.659 | 5.661 | 5.663 | 5.664 | 5.666 | 5.668 | 5.669 | 5.671 | 5.673 | 5.674 |
| | | | | | | | | | | | | | | | | |
| −4.096 | −4.186 | −4.277 | −4.372 | −4.469 | −4.568 | −4.670 | −4.773 | −4.878 | −4.985 | −5.093 | −5.202 | −5.313 | −5.425 | −5.538 | −5.652 | −5.767 |
| −1.434 | −1.482 | −1.536 | −1.594 | −1.656 | −1.723 | −1.796 | −1.872 | −1.954 | −2.040 | −2.130 | −2.226 | −2.326 | −2.430 | −2.539 | −2.651 | −2.769 |
| | | | | | | | | | | | | | | | | |
| −5.035 | −5.113 | −5.193 | −5.277 | −5.363 | −5.453 | −5.546 | −5.642 | −5.741 | −5.843 | −5.948 | −6.055 | −6.164 | −6.276 | −6.390 | −6.506 | −6.623 |
| −3.371 | −3.409 | −3.451 | −3.496 | −3.544 | −3.595 | −3.650 | −3.707 | −3.768 | −3.832 | −3.899 | −3.970 | −4.044 | −4.121 | −4.201 | −4.285 | −4.372 |

| | | | | | | | | | | | | | | | | |
|---|---|---|---|---|---|---|---|---|---|---|---|---|---|---|---|---|
| 5.676 | 5.678 | 5.679 | 5.681 | 5.683 | 5.684 | 5.686 | 5.688 | 5.689 | 5.691 | 5.692 | 5.694 | 5.696 | 5.697 | 5.699 | 5.701 | 5.702 |
| −5.883 | −5.999 | −6.115 | −6.231 | −6.346 | −6.461 | −6.575 | −6.688 | −6.800 | −6.911 | −7.021 | −7.130 | −7.237 | −7.342 | −7.447 | −7.549 | −7.650 |
| −2.890 | −3.016 | −3.145 | −3.278 | −3.414 | −3.554 | −3.697 | −3.843 | −3.992 | −4.144 | −4.298 | −4.455 | −4.613 | −4.774 | −4.936 | −5.100 | −5.265 |
| −6.742 | −6.862 | −6.982 | −7.102 | −7.223 | −7.344 | −7.465 | −7.586 | −7.707 | −7.827 | −7.946 | −8.064 | −8.182 | −8.298 | −8.413 | −8.527 | −8.640 |
| −4.462 | −4.555 | −4.651 | −4.750 | −4.851 | −4.956 | −5.062 | −5.172 | −5.283 | −5.396 | −5.511 | −5.628 | −5.746 | −5.866 | −5.987 | −6.109 | −6.232 |

| | | | | | | | | | | | | | | | | |
|---|---|---|---|---|---|---|---|---|---|---|---|---|---|---|---|---|
| 5.704 | 5.706 | 5.707 | 5.709 | 5.711 | 5.712 | 5.714 | 5.716 | 5.717 | 5.719 | 5.721 | 5.722 | 5.724 | 5.726 | 5.727 | 5.729 | 5.731 |
| −7.748 | −7.845 | −7.939 | −8.031 | −8.121 | −8.210 | −8.295 | −8.379 | −8.460 | −8.540 | −8.617 | −8.692 | −8.765 | −8.836 | −8.904 | −8.971 | −9.035 |
| −5.431 | −5.598 | −5.766 | −5.933 | −6.101 | −6.268 | −6.435 | −6.601 | −6.765 | −6.928 | −7.089 | −7.248 | −7.405 | −7.560 | −7.713 | −7.864 | −8.011 |
| −8.752 | −8.863 | −8.972 | −9.081 | −9.187 | −9.292 | −9.394 | −9.495 | −9.593 | −9.689 | −9.784 | −9.876 | −9.965 | −10.053 | −10.138 | −10.222 | −10.303 |
| −6.356 | −6.481 | −6.607 | −6.732 | −6.858 | −6.984 | −7.110 | −7.236 | −7.360 | −7.485 | −7.607 | −7.729 | −7.849 | −7.967 | −8.084 | −8.198 | −8.311 |

| | | | | | | | | | | | | | | | | |
|---|---|---|---|---|---|---|---|---|---|---|---|---|---|---|---|---|
| 5.732 | 5.734 | 5.736 | 5.737 | 5.739 | 5.741 | 5.742 | 5.744 | 5.746 | 5.747 | 5.749 | 5.751 | 5.752 | 5.754 | 5.756 | 5.757 | 5.759 |
| | | | | | | | | | | | | | | | | |
| −9.098 | −9.158 | −9.216 | −9.273 | −9.327 | −9.379 | −9.430 | −9.479 | −9.525 | −9.571 | −9.615 | −9.658 | −9.700 | −9.741 | −9.780 | −9.819 | −9.857 |
| −8.157 | −8.299 | −8.439 | −8.576 | −8.711 | −8.842 | −8.971 | −9.097 | −9.220 | −9.339 | −9.457 | −9.570 | −9.681 | −9.788 | −9.893 | −9.995 | −10.094 |
| | | | | | | | | | | | | | | | | |
| −10.381 | −10.458 | −10.533 | −10.606 | −10.676 | −10.745 | −10.812 | −10.878 | −10.941 | −11.003 | −11.062 | −11.121 | −11.177 | −11.233 | −11.287 | −11.340 | −11.392 |
| −8.421 | −8.529 | −8.635 | −8.738 | −8.839 | −8.936 | −9.031 | −9.123 | −9.212 | −9.298 | −9.381 | −9.461 | −9.538 | −9.612 | −9.682 | −9.749 | −9.814 |

| | | | | | | | | | | | | | | | | |
|---|---|---|---|---|---|---|---|---|---|---|---|---|---|---|---|---|
| 5.761 | 5.762 | 5.764 | 5.766 | 5.767 | 5.769 | 5.771 | 5.772 | 5.774 | 5.776 | 5.777 | 5.779 | 5.781 | 5.782 | 5.784 | 5.786 | 5.787 |
| | | | | | | | | | | | | | | | | |
| −9.894 | −9.931 | −9.967 | −10.003 | −10.039 | −10.074 | −10.109 | −10.144 | −10.178 | −10.213 | −10.247 | −10.282 | −10.316 | −10.351 | −10.386 | −10.421 | −10.456 |
| −10.189 | −10.283 | −10.372 | −10.459 | −10.543 | −10.624 | −10.702 | −10.777 | −10.849 | −10.919 | −10.986 | −11.050 | −11.112 | −11.171 | −11.227 | −11.281 | −11.332 |
| | | | | | | | | | | | | | | | | |
| −11.443 | −11.493 | −11.543 | −11.592 | −11.640 | −11.688 | −11.736 | −11.784 | −11.831 | −11.879 | −11.925 | −11.973 | −12.020 | −12.068 | −12.115 | −12.164 | −12.213 |
| −9.875 | −9.934 | −9.989 | −10.042 | −10.092 | −10.139 | −10.183 | −10.225 | −10.264 | −10.300 | −10.334 | −10.365 | −10.393 | −10.419 | −10.442 | −10.463 | −10.482 |

| | | | | | | | | | | | | | | | | |
|---|---|---|---|---|---|---|---|---|---|---|---|---|---|---|---|---|
| 5.789 | 5.791 | 5.792 | 5.794 | 5.796 | 5.797 | 5.799 | 5.801 | 5.802 | 5.804 | 5.806 | 5.807 | 5.809 | 5.811 | 5.812 | 5.814 | 5.816 |
| −10.492 | −10.528 | −10.564 | −10.601 | −10.639 | −10.677 | −10.717 | −10.757 | −10.799 | −10.843 | −10.888 | −10.936 | −10.985 | −11.036 | −11.090 | −11.146 | −11.204 |
| −11.381 | −11.428 | −11.473 | −11.517 | −11.558 | −11.598 | −11.636 | −11.674 | −11.709 | −11.745 | −11.778 | −11.812 | −11.843 | −11.876 | −11.907 | −11.938 | −11.969 |
| −12.264 | −12.315 | −12.367 | −12.420 | −12.474 | −12.529 | −12.585 | −12.642 | −12.701 | −12.760 | −12.820 | −12.882 | −12.945 | −13.010 | −13.076 | −13.144 | −13.213 |
| −10.499 | −10.514 | −10.527 | −10.539 | −10.549 | −10.559 | −10.566 | −10.574 | −10.579 | −10.585 | −10.589 | −10.594 | −10.598 | −10.601 | −10.604 | −10.608 | −10.611 |

| | | | | | | | | | | | | | | | | |
|---|---|---|---|---|---|---|---|---|---|---|---|---|---|---|---|---|
| 5.817 | 5.819 | 5.821 | 5.822 | 5.824 | 5.826 | 5.827 | 5.829 | 5.831 | 5.832 | 5.834 | 5.836 | 5.837 | 5.839 | 5.841 | 5.842 | 5.844 |
| | | | | | | | | | | | | | | | | |
| −11.266 | −11.328 | −11.394 | −11.460 | −11.531 | −11.602 | −11.676 | −11.751 | −11.829 | −11.908 | −11.989 | −12.071 | −12.154 | −12.238 | −12.324 | −12.410 | −12.497 |
| −12.000 | −12.031 | −12.062 | −12.094 | −12.125 | −12.158 | −12.190 | −12.223 | −12.257 | −12.292 | −12.326 | −12.363 | −12.399 | −12.437 | −12.475 | −12.515 | −12.555 |
| | | | | | | | | | | | | | | | | |
| −13.284 | −13.357 | −13.431 | −13.507 | −13.585 | −13.663 | −13.744 | −13.825 | −13.909 | −13.993 | −14.079 | −14.165 | −14.253 | −14.341 | −14.430 | −14.519 | −14.609 |
| −10.614 | −10.618 | −10.622 | −10.627 | −10.633 | −10.640 | −10.648 | −10.657 | −10.667 | −10.678 | −10.691 | −10.705 | −10.721 | −10.738 | −10.756 | −10.775 | −10.795 |

| | | | | | | | | | | | | | | | | |
|---|---|---|---|---|---|---|---|---|---|---|---|---|---|---|---|---|
| 5.846 | 5.847 | 5.849 | 5.851 | 5.852 | 5.854 | 5.856 | 5.857 | 5.859 | 5.861 | 5.862 | 5.864 | 5.866 | 5.867 | 5.869 | 5.871 | 5.872 |
| | | | | | | | | | | | | | | | | |
| −12.584 | −12.671 | −12.758 | −12.845 | −12.931 | −13.017 | −13.103 | −13.188 | −13.273 | −13.357 | −13.442 | −13.525 | −13.609 | −13.692 | −13.775 | −13.857 | −13.940 |
| −12.596 | −12.637 | −12.680 | −12.723 | −12.768 | −12.813 | −12.860 | −12.907 | −12.957 | −13.006 | −13.058 | −13.109 | −13.163 | −13.217 | −13.272 | −13.328 | −13.385 |
| | | | | | | | | | | | | | | | | |
| −14.698 | −14.787 | −14.876 | −14.965 | −15.054 | −15.142 | −15.231 | −15.318 | −15.406 | −15.492 | −15.579 | −15.664 | −15.749 | −15.832 | −15.916 | −15.998 | −16.080 |
| −10.818 | −10.841 | −10.866 | −10.892 | −10.920 | −10.949 | −10.981 | −11.014 | −11.048 | −11.084 | −11.122 | −11.162 | −11.203 | −11.246 | −11.291 | −11.338 | −11.387 |

| | | | | | | | | | | | | | | | | |
|---|---|---|---|---|---|---|---|---|---|---|---|---|---|---|---|---|
| 5.874 | 5.876 | 5.877 | 5.879 | 5.881 | 5.882 | 5.884 | 5.886 | 5.887 | 5.889 | 5.891 | 5.892 | 5.894 | 5.896 | 5.897 | 5.899 | 5.901 |
| −14.021 | −14.103 | −14.184 | −14.265 | −14.344 | −14.424 | −14.502 | −14.579 | −14.655 | −14.730 | −14.803 | −14.875 | −14.946 | −15.015 | −15.083 | −15.150 | −15.215 |
| −13.443 | −13.502 | −13.562 | −13.623 | −13.685 | −13.748 | −13.811 | −13.875 | −13.940 | −14.004 | −14.070 | −14.135 | −14.200 | −14.265 | −14.330 | −14.395 | −14.460 |
| −16.160 | −16.240 | −16.319 | −16.397 | −16.473 | −16.549 | −16.622 | −16.694 | −16.764 | −16.832 | −16.898 | −16.962 | −17.023 | −17.083 | −17.140 | −17.194 | −17.246 |
| −11.437 | −11.489 | −11.542 | −11.597 | −11.653 | −11.710 | −11.768 | −11.828 | −11.889 | −11.951 | −12.014 | −12.078 | −12.143 | −12.209 | −12.275 | −12.342 | −12.410 |

| | | | | | | | | | | | | | | | | |
|---|---|---|---|---|---|---|---|---|---|---|---|---|---|---|---|---|
| 5.902 | 5.904 | 5.906 | 5.907 | 5.909 | 5.911 | 5.912 | 5.914 | 5.916 | 5.917 | 5.919 | 5.921 | 5.922 | 5.924 | 5.926 | 5.927 | 5.929 |
| | | | | | | | | | | | | | | | | |
| −15.278 | −15.339 | −15.398 | −15.457 | −15.512 | −15.567 | −15.618 | −15.668 | −15.716 | −15.761 | −15.804 | −15.845 | −15.883 | −15.919 | −15.952 | −15.984 | −16.013 |
| −14.525 | −14.589 | −14.652 | −14.715 | −14.778 | −14.840 | −14.902 | −14.962 | −15.022 | −15.081 | −15.139 | −15.197 | −15.253 | −15.308 | −15.362 | −15.415 | −15.467 |
| | | | | | | | | | | | | | | | | |
| −17.296 | −17.344 | −17.389 | −17.432 | −17.472 | −17.510 | −17.544 | −17.576 | −17.605 | −17.631 | −17.654 | −17.676 | −17.695 | −17.712 | −17.726 | −17.740 | −17.750 |
| −12.478 | −12.546 | −12.615 | −12.685 | −12.755 | −12.825 | −12.895 | −12.965 | −13.036 | −13.107 | −13.179 | −13.250 | −13.322 | −13.394 | −13.466 | −13.538 | −13.610 |

| | | | | | | | | | | | | | | | | |
|---|---|---|---|---|---|---|---|---|---|---|---|---|---|---|---|---|
| 5.931 | 5.932 | 5.934 | 5.936 | 5.937 | 5.939 | 5.941 | 5.942 | 5.944 | 5.946 | 5.947 | 5.949 | 5.951 | 5.952 | 5.954 | 5.956 | 5.957 |
| −16.040 | −16.065 | −16.088 | −16.109 | −16.127 | −16.144 | −16.158 | −16.170 | −16.181 | −16.190 | −16.197 | −16.204 | −16.209 | −16.213 | −16.216 | −16.218 | −16.218 |
| −15.518 | −15.568 | −15.617 | −15.665 | −15.712 | −15.758 | −15.803 | −15.846 | −15.889 | −15.931 | −15.972 | −16.011 | −16.050 | −16.087 | −16.123 | −16.158 | −16.192 |
| −17.759 | −17.766 | −17.771 | −17.774 | −17.776 | −17.775 | −17.772 | −17.768 | −17.762 | −17.754 | −17.744 | −17.733 | −17.719 | −17.704 | −17.687 | −17.669 | −17.649 |
| −13.682 | −13.754 | −13.826 | −13.897 | −13.969 | −14.040 | −14.111 | −14.181 | −14.251 | −14.319 | −14.388 | −14.455 | −14.522 | −14.587 | −14.652 | −14.714 | −14.776 |

| | | | | | | | | | | | | | | | | |
|---|---|---|---|---|---|---|---|---|---|---|---|---|---|---|---|---|
| 5.959 | 5.961 | 5.962 | 5.964 | 5.966 | 5.967 | 5.969 | 5.971 | 5.972 | 5.974 | 5.976 | 5.977 | 5.979 | 5.981 | 5.982 | 5.984 | 5.986 |
| −16.218 | −16.217 | −16.216 | −16.214 | −16.211 | −16.208 | −16.204 | −16.200 | −16.195 | −16.190 | −16.185 | −16.179 | −16.173 | −16.167 | −16.160 | −16.153 | −16.146 |
| −16.225 | −16.257 | −16.288 | −16.318 | −16.347 | −16.375 | −16.403 | −16.430 | −16.457 | −16.484 | −16.511 | −16.537 | −16.564 | −16.590 | −16.617 | −16.644 | −16.671 |
| −17.628 | −17.605 | −17.581 | −17.556 | −17.531 | −17.503 | −17.476 | −17.448 | −17.420 | −17.390 | −17.361 | −17.331 | −17.301 | −17.271 | −17.241 | −17.211 | −17.180 |
| −14.836 | −14.894 | −14.950 | −15.006 | −15.058 | −15.110 | −15.159 | −15.207 | −15.254 | −15.299 | −15.342 | −15.385 | −15.426 | −15.466 | −15.506 | −15.545 | −15.583 |

| | | | | | | | | | | | | | | | | |
|---|---|---|---|---|---|---|---|---|---|---|---|---|---|---|---|---|
| 5.987 | 5.989 | 5.991 | 5.992 | 5.994 | 5.996 | 5.997 | 5.999 | 6.001 | 6.002 | 6.004 | 6.006 | 6.007 | 6.009 | 6.011 | 6.012 | 6.014 |
| | | | | | | | | | | | | | | | | |
| −16.139 | −16.132 | −16.126 | −16.119 | −16.113 | −16.106 | −16.101 | −16.095 | −16.090 | −16.085 | −16.081 | −16.078 | −16.075 | −16.073 | −16.071 | −16.070 | −16.070 |
| −16.698 | −16.726 | −16.754 | −16.783 | −16.812 | −16.843 | −16.873 | −16.904 | −16.935 | −16.968 | −17.001 | −17.035 | −17.069 | −17.104 | −17.138 | −17.174 | −17.209 |
| | | | | | | | | | | | | | | | | |
| −17.151 | −17.121 | −17.093 | −17.064 | −17.037 | −17.010 | −16.985 | −16.961 | −16.939 | −16.917 | −16.897 | −16.878 | −16.862 | −16.846 | −16.832 | −16.820 | −16.810 |
| −15.620 | −15.658 | −15.694 | −15.731 | −15.767 | −15.804 | −15.840 | −15.876 | −15.911 | −15.947 | −15.982 | −16.018 | −16.052 | −16.088 | −16.122 | −16.158 | −16.193 |

| | | | | | | | | | | | | | | | | |
|---|---|---|---|---|---|---|---|---|---|---|---|---|---|---|---|---|
| 6.016 | 6.017 | 6.019 | 6.020 | 6.022 | 6.024 | 6.025 | 6.027 | 6.029 | 6.030 | 6.032 | 6.034 | 6.035 | 6.037 | 6.039 | 6.040 | 6.042 |
| | | | | | | | | | | | | | | | | |
| −16.070 | −16.071 | −16.073 | −16.075 | −16.078 | −16.080 | −16.083 | −16.085 | −16.088 | −16.090 | −16.092 | −16.094 | −16.096 | −16.096 | −16.098 | −16.098 | −16.099 |
| −17.244 | −17.279 | −17.313 | −17.347 | −17.382 | −17.415 | −17.449 | −17.482 | −17.514 | −17.546 | −17.577 | −17.608 | −17.638 | −17.667 | −17.695 | −17.723 | −17.750 |
| | | | | | | | | | | | | | | | | |
| −16.801 | −16.794 | −16.787 | −16.783 | −16.779 | −16.777 | −16.776 | −16.775 | −16.775 | −16.776 | −16.777 | −16.778 | −16.781 | −16.783 | −16.787 | −16.789 | −16.792 |
| −16.228 | −16.262 | −16.297 | −16.331 | −16.365 | −16.398 | −16.432 | −16.464 | −16.496 | −16.528 | −16.559 | −16.589 | −16.619 | −16.648 | −16.676 | −16.703 | −16.729 |

| | | | | | | | | | | | | | | | | |
|---|---|---|---|---|---|---|---|---|---|---|---|---|---|---|---|---|
| 6.044 | 6.045 | 6.047 | 6.049 | 6.050 | 6.052 | 6.054 | 6.055 | 6.057 | 6.059 | 6.060 | 6.062 | 6.064 | 6.065 | 6.067 | 6.069 | 6.070 |
| −16.098 | −16.097 | −16.094 | −16.092 | −16.088 | −16.083 | −16.077 | −16.070 | −16.062 | −16.052 | −16.041 | −16.029 | −16.016 | −16.002 | −15.986 | −15.969 | −15.951 |
| −17.775 | −17.801 | −17.824 | −17.847 | −17.868 | −17.889 | −17.907 | −17.925 | −17.942 | −17.957 | −17.971 | −17.983 | −17.996 | −18.006 | −18.016 | −18.024 | −18.033 |
| −16.795 | −16.798 | −16.800 | −16.803 | −16.804 | −16.806 | −16.806 | −16.807 | −16.807 | −16.807 | −16.806 | −16.805 | −16.803 | −16.800 | −16.798 | −16.794 | −16.791 |
| −16.754 | −16.779 | −16.802 | −16.824 | −16.845 | −16.864 | −16.882 | −16.899 | −16.915 | −16.930 | −16.943 | −16.955 | −16.966 | −16.974 | −16.983 | −16.989 | −16.995 |

| | | | | | | | | | | | | | | | | |
|---|---|---|---|---|---|---|---|---|---|---|---|---|---|---|---|---|
| 6.072 | 6.074 | 6.075 | 6.077 | 6.079 | 6.080 | 6.082 | 6.084 | 6.085 | 6.087 | 6.089 | 6.090 | 6.092 | 6.094 | 6.095 | 6.097 | 6.099 |
| −15.931 | −15.911 | −15.889 | −15.867 | −15.843 | −15.819 | −15.793 | −15.767 | −15.739 | −15.712 | −15.683 | −15.655 | −15.625 | −15.596 | −15.565 | −15.535 | −15.503 |
| −18.038 | −18.045 | −18.048 | −18.053 | −18.054 | −18.056 | −18.056 | −18.056 | −18.054 | −18.053 | −18.049 | −18.046 | −18.041 | −18.036 | −18.029 | −18.022 | −18.013 |
| −16.787 | −16.783 | −16.779 | −16.774 | −16.768 | −16.763 | −16.755 | −16.749 | −16.740 | −16.732 | −16.722 | −16.712 | −16.700 | −16.689 | −16.675 | −16.662 | −16.647 |
| −16.999 | −17.003 | −17.005 | −17.007 | −17.006 | −17.006 | −17.003 | −17.001 | −16.996 | −16.991 | −16.983 | −16.976 | −16.966 | −16.956 | −16.943 | −16.931 | −16.918 |

| | | | | | | | | | | | | | | | | |
|---|---|---|---|---|---|---|---|---|---|---|---|---|---|---|---|---|
| 6.100 | 6.102 | 6.104 | 6.105 | 6.107 | 6.109 | 6.110 | 6.112 | 6.114 | 6.115 | 6.117 | 6.119 | 6.120 | 6.122 | 6.124 | 6.125 | 6.127 |
| −15.472 | −15.439 | −15.406 | −15.371 | −15.336 | −15.299 | −15.263 | −15.225 | −15.187 | −15.149 | −15.110 | −15.072 | −15.033 | −14.994 | −14.955 | −14.916 | −14.876 |
| −18.004 | −17.992 | −17.980 | −17.967 | −17.953 | −17.938 | −17.920 | −17.903 | −17.882 | −17.862 | −17.838 | −17.815 | −17.788 | −17.762 | −17.732 | −17.703 | −17.671 |
| −16.633 | −16.617 | −16.601 | −16.584 | −16.567 | −16.549 | −16.530 | −16.511 | −16.492 | −16.472 | −16.451 | −16.431 | −16.409 | −16.388 | −16.366 | −16.344 | −16.321 |
| −16.904 | −16.889 | −16.873 | −16.857 | −16.840 | −16.823 | −16.805 | −16.787 | −16.767 | −16.747 | −16.725 | −16.705 | −16.681 | −16.659 | −16.633 | −16.609 | −16.581 |

| | | | | | | | | | | | | | | | | |
|---|---|---|---|---|---|---|---|---|---|---|---|---|---|---|---|---|
| 6.129 | 6.130 | 6.132 | 6.134 | 6.135 | 6.137 | 6.139 | 6.140 | 6.142 | 6.144 | 6.145 | 6.147 | 6.149 | 6.150 | 6.152 | 6.154 | 6.155 |
| −14.838 | −14.798 | −14.760 | −14.720 | −14.682 | −14.642 | −14.604 | −14.564 | −14.526 | −14.486 | −14.447 | −14.407 | −14.368 | −14.328 | −14.289 | −14.250 | −14.211 |
| −17.641 | −17.607 | −17.574 | −17.539 | −17.504 | −17.467 | −17.432 | −17.394 | −17.357 | −17.318 | −17.280 | −17.241 | −17.202 | −17.162 | −17.122 | −17.082 | −17.042 |
| −16.299 | −16.276 | −16.253 | −16.230 | −16.206 | −16.181 | −16.156 | −16.130 | −16.103 | −16.075 | −16.047 | −16.018 | −15.987 | −15.956 | −15.924 | −15.892 | −15.858 |
| −16.555 | −16.526 | −16.498 | −16.468 | −16.439 | −16.407 | −16.377 | −16.346 | −16.315 | −16.283 | −16.252 | −16.220 | −16.189 | −16.157 | −16.124 | −16.092 | −16.059 |

| | | | | | | | | | | | | | | | | |
|---|---|---|---|---|---|---|---|---|---|---|---|---|---|---|---|---|
| 6.157 | 6.159 | 6.160 | 6.162 | 6.164 | 6.165 | 6.167 | 6.169 | 6.170 | 6.172 | 6.174 | 6.175 | 6.177 | 6.179 | 6.180 | 6.182 | 6.184 |
| | | | | | | | | | | | | | | | | |
| −14.172 | −14.133 | −14.095 | −14.056 | −14.018 | −13.980 | −13.943 | −13.905 | −13.868 | −13.832 | −13.796 | −13.760 | −13.725 | −13.690 | −13.655 | −13.621 | −13.588 |
| −17.002 | −16.961 | −16.921 | −16.881 | −16.841 | −16.801 | −16.762 | −16.721 | −16.681 | −16.639 | −16.599 | −16.557 | −16.517 | −16.476 | −16.436 | −16.395 | −16.356 |
| | | | | | | | | | | | | | | | | |
| −15.824 | −15.789 | −15.754 | −15.718 | −15.682 | −15.645 | −15.608 | −15.570 | −15.531 | −15.492 | −15.452 | −15.411 | −15.369 | −15.327 | −15.283 | −15.239 | −15.193 |
| −16.027 | −15.994 | −15.962 | −15.929 | −15.897 | −15.864 | −15.833 | −15.800 | −15.769 | −15.738 | −15.707 | −15.676 | −15.646 | −15.615 | −15.585 | −15.555 | −15.525 |

| | | | | | | | | | | | | | | | | |
|---|---|---|---|---|---|---|---|---|---|---|---|---|---|---|---|---|
| 6.185 | 6.187 | 6.189 | 6.190 | 6.192 | 6.194 | 6.195 | 6.197 | 6.199 | 6.200 | 6.202 | 6.204 | 6.205 | 6.207 | 6.209 | 6.210 | 6.212 |
| | | | | | | | | | | | | | | | | |
| −13.555 | −13.523 | −13.491 | −13.460 | −13.431 | −13.401 | −13.374 | −13.346 | −13.321 | −13.296 | −13.273 | −13.250 | −13.229 | −13.209 | −13.190 | −13.173 | −13.156 |
| −16.316 | −16.276 | −16.237 | −16.197 | −16.158 | −16.118 | −16.079 | −16.039 | −16.000 | −15.960 | −15.921 | −15.881 | −15.841 | −15.801 | −15.762 | −15.722 | −15.683 |
| | | | | | | | | | | | | | | | | |
| −15.147 | −15.098 | −15.051 | −15.001 | −14.953 | −14.903 | −14.854 | −14.805 | −14.756 | −14.708 | −14.660 | −14.612 | −14.566 | −14.520 | −14.475 | −14.431 | −14.387 |
| −15.495 | −15.465 | −15.435 | −15.405 | −15.375 | −15.345 | −15.316 | −15.287 | −15.258 | −15.229 | −15.200 | −15.171 | −15.143 | −15.114 | −15.086 | −15.057 | −15.028 |

| | | | | | | | | | | | | | | | | |
|---|---|---|---|---|---|---|---|---|---|---|---|---|---|---|---|---|
| 6.214 | 6.215 | 6.217 | 6.219 | 6.220 | 6.222 | 6.224 | 6.225 | 6.227 | 6.229 | 6.230 | 6.232 | 6.234 | 6.235 | 6.237 | 6.239 | 6.240 |
| −13.140 | −13.126 | −13.113 | −13.100 | −13.089 | −13.078 | −13.070 | −13.061 | −13.054 | −13.045 | −13.039 | −13.032 | −13.027 | −13.020 | −13.015 | −13.009 | −13.005 |
| −15.643 | −15.604 | −15.565 | −15.525 | −15.486 | −15.447 | −15.407 | −15.368 | −15.329 | −15.290 | −15.251 | −15.212 | −15.174 | −15.135 | −15.097 | −15.058 | −15.021 |
| −14.346 | −14.304 | −14.264 | −14.223 | −14.185 | −14.145 | −14.108 | −14.069 | −14.033 | −13.995 | −13.960 | −13.923 | −13.888 | −13.852 | −13.818 | −13.782 | −13.749 |
| −15.000 | −14.972 | −14.944 | −14.915 | −14.888 | −14.859 | −14.832 | −14.804 | −14.778 | −14.750 | −14.724 | −14.696 | −14.670 | −14.642 | −14.615 | −14.587 | −14.560 |

| | | | | | | | | | | | | | | | | |
|---|---|---|---|---|---|---|---|---|---|---|---|---|---|---|---|---|
| 6.242 | 6.244 | 6.245 | 6.247 | 6.249 | 6.250 | 6.252 | 6.254 | 6.255 | 6.257 | 6.259 | 6.260 | 6.262 | 6.264 | 6.265 | 6.267 | 6.269 |
| −12.998 | −12.994 | −12.988 | −12.984 | −12.977 | −12.972 | −12.966 | −12.960 | −12.953 | −12.946 | −12.939 | −12.930 | −12.922 | −12.913 | −12.905 | −12.894 | −12.886 |
| −14.984 | −14.947 | −14.910 | −14.874 | −14.837 | −14.801 | −14.764 | −14.728 | −14.692 | −14.655 | −14.619 | −14.582 | −14.545 | −14.508 | −14.472 | −14.434 | −14.398 |
| −13.714 | −13.681 | −13.647 | −13.615 | −13.581 | −13.548 | −13.514 | −13.481 | −13.447 | −13.414 | −13.382 | −13.348 | −13.317 | −13.284 | −13.254 | −13.223 | −13.194 |
| −14.531 | −14.503 | −14.473 | −14.443 | −14.412 | −14.382 | −14.350 | −14.318 | −14.285 | −14.251 | −14.218 | −14.183 | −14.149 | −14.113 | −14.078 | −14.040 | −14.005 |

| | | | | | | | | | | | | | | | | |
|---|---|---|---|---|---|---|---|---|---|---|---|---|---|---|---|---|
| 6.270 | 6.272 | 6.274 | 6.275 | 6.277 | 6.279 | 6.280 | 6.282 | 6.284 | 6.285 | 6.287 | 6.289 | 6.290 | 6.292 | 6.294 | 6.295 | 6.297 |
| −12.875 | −12.866 | −12.853 | −12.844 | −12.831 | −12.820 | −12.806 | −12.795 | −12.780 | −12.768 | −12.753 | −12.740 | −12.724 | −12.711 | −12.695 | −12.681 | −12.665 |
| −14.360 | −14.323 | −14.285 | −14.248 | −14.209 | −14.172 | −14.133 | −14.095 | −14.056 | −14.018 | −13.979 | −13.942 | −13.903 | −13.866 | −13.828 | −13.792 | −13.756 |
| −13.164 | −13.137 | −13.108 | −13.082 | −13.054 | −13.030 | −13.003 | −12.980 | −12.955 | −12.934 | −12.911 | −12.891 | −12.871 | −12.853 | −12.835 | −12.819 | −12.803 |
| −13.966 | −13.929 | −13.889 | −13.851 | −13.809 | −13.769 | −13.726 | −13.685 | −13.641 | −13.599 | −13.554 | −13.511 | −13.466 | −13.422 | −13.377 | −13.332 | −13.287 |

| | | | | | | | | | | | | | | | | |
|---|---|---|---|---|---|---|---|---|---|---|---|---|---|---|---|---|
| 6.299 | 6.300 | 6.302 | 6.304 | 6.305 | 6.307 | 6.309 | 6.310 | 6.312 | 6.314 | 6.315 | 6.317 | 6.319 | 6.320 | 6.322 | 6.324 | 6.325 |
| | | | | | | | | | | | | | | | | |
| −12.652 | −12.636 | −12.622 | −12.607 | −12.592 | −12.577 | −12.562 | −12.547 | −12.532 | −12.516 | −12.501 | −12.485 | −12.469 | −12.454 | −12.437 | −12.422 | −12.405 |
| −13.721 | −13.687 | −13.654 | −13.621 | −13.590 | −13.559 | −13.529 | −13.501 | −13.473 | −13.447 | −13.420 | −13.396 | −13.372 | −13.350 | −13.327 | −13.307 | −13.287 |
| | | | | | | | | | | | | | | | | |
| −12.788 | −12.774 | −12.760 | −12.747 | −12.735 | −12.722 | −12.710 | −12.698 | −12.685 | −12.673 | −12.660 | −12.649 | −12.635 | −12.623 | −12.609 | −12.597 | −12.582 |
| −13.242 | −13.197 | −13.151 | −13.106 | −13.061 | −13.016 | −12.971 | −12.927 | −12.882 | −12.839 | −12.794 | −12.752 | −12.708 | −12.666 | −12.622 | −12.581 | −12.537 |

| | | | | | | | | | | | | | | | | |
|---|---|---|---|---|---|---|---|---|---|---|---|---|---|---|---|---|
| 6.327 | 6.329 | 6.330 | 6.332 | 6.334 | 6.335 | 6.337 | 6.339 | 6.340 | 6.342 | 6.343 | 6.345 | 6.347 | 6.348 | 6.350 | 6.352 | 6.353 |
| | | | | | | | | | | | | | | | | |
| −12.389 | −12.372 | −12.356 | −12.339 | −12.323 | −12.304 | −12.287 | −12.267 | −12.249 | −12.228 | −12.208 | −12.186 | −12.165 | −12.142 | −12.121 | −12.097 | −12.076 |
| −13.269 | −13.251 | −13.235 | −13.219 | −13.204 | −13.189 | −13.176 | −13.162 | −13.150 | −13.137 | −13.126 | −13.114 | −13.104 | −13.093 | −13.084 | −13.074 | −13.065 |
| | | | | | | | | | | | | | | | | |
| −12.569 | −12.554 | −12.540 | −12.524 | −12.509 | −12.492 | −12.477 | −12.461 | −12.445 | −12.429 | −12.414 | −12.397 | −12.382 | −12.366 | −12.352 | −12.337 | −12.324 |
| −12.496 | −12.453 | −12.412 | −12.369 | −12.328 | −12.285 | −12.244 | −12.202 | −12.161 | −12.119 | −12.078 | −12.037 | −11.996 | −11.955 | −11.915 | −11.874 | −11.834 |

| | | | | | | | | | | | | | | | | |
|---|---|---|---|---|---|---|---|---|---|---|---|---|---|---|---|---|
| 6.355 | 6.357 | 6.358 | 6.360 | 6.362 | 6.363 | 6.365 | 6.367 | 6.368 | 6.370 | 6.372 | 6.373 | 6.375 | 6.377 | 6.378 | 6.380 | 6.382 |
| −12.053 | −12.033 | −12.010 | −11.990 | −11.968 | −11.949 | −11.927 | −11.908 | −11.887 | −11.868 | −11.847 | −11.829 | −11.808 | −11.789 | −11.769 | −11.750 | −11.731 |
| −13.056 | −13.048 | −13.039 | −13.032 | −13.023 | −13.016 | −13.007 | −13.001 | −12.992 | −12.985 | −12.976 | −12.969 | −12.960 | −12.952 | −12.942 | −12.933 | −12.922 |
| −12.309 | −12.298 | −12.284 | −12.273 | −12.261 | −12.251 | −12.240 | −12.232 | −12.222 | −12.216 | −12.207 | −12.202 | −12.194 | −12.189 | −12.183 | −12.178 | −12.172 |
| −11.794 | −11.756 | −11.717 | −11.679 | −11.641 | −11.605 | −11.568 | −11.533 | −11.497 | −11.463 | −11.429 | −11.396 | −11.363 | −11.331 | −11.300 | −11.269 | −11.238 |

| | | | | | | | | | | | | | | | | |
|---|---|---|---|---|---|---|---|---|---|---|---|---|---|---|---|---|
| 6.383 | 6.385 | 6.387 | 6.388 | 6.390 | 6.392 | 6.393 | 6.395 | 6.397 | 6.398 | 6.400 | 6.402 | 6.403 | 6.405 | 6.407 | 6.408 | 6.410 |
| | | | | | | | | | | | | | | | | |
| −11.711 | −11.692 | −11.673 | −11.655 | −11.636 | −11.618 | −11.598 | −11.582 | −11.563 | −11.548 | −11.530 | −11.517 | −11.500 | −11.488 | −11.473 | −11.463 | −11.448 |
| −12.911 | −12.899 | −12.886 | −12.871 | −12.856 | −12.840 | −12.822 | −12.803 | −12.783 | −12.763 | −12.740 | −12.718 | −12.693 | −12.670 | −12.643 | −12.619 | −12.591 |
| | | | | | | | | | | | | | | | | |
| −12.167 | −12.162 | −12.156 | −12.151 | −12.145 | −12.140 | −12.133 | −12.128 | −12.120 | −12.115 | −12.108 | −12.104 | −12.097 | −12.094 | −12.088 | −12.086 | −12.081 |
| −11.209 | −11.179 | −11.151 | −11.122 | −11.095 | −11.068 | −11.041 | −11.016 | −10.991 | −10.968 | −10.943 | −10.922 | −10.899 | −10.880 | −10.858 | −10.841 | −10.822 |

| | | | | | | | | | | | | | | | | |
|---|---|---|---|---|---|---|---|---|---|---|---|---|---|---|---|---|
| 6.412 | 6.413 | 6.415 | 6.417 | 6.418 | 6.420 | 6.422 | 6.423 | 6.425 | 6.427 | 6.428 | 6.430 | 6.432 | 6.433 | 6.435 | 6.437 | 6.438 |
| −11.439 | −11.426 | −11.419 | −11.408 | −11.402 | −11.393 | −11.389 | −11.381 | −11.379 | −11.373 | −11.371 | −11.368 | −11.367 | −11.365 | −11.364 | −11.363 | −11.362 |
| −12.565 | −12.535 | −12.508 | −12.477 | −12.450 | −12.418 | −12.389 | −12.357 | −12.328 | −12.296 | −12.266 | −12.234 | −12.203 | −12.171 | −12.140 | −12.107 | −12.075 |
| −12.081 | −12.077 | −12.078 | −12.076 | −12.078 | −12.078 | −12.081 | −12.082 | −12.087 | −12.090 | −12.095 | −12.100 | −12.106 | −12.111 | −12.117 | −12.124 | −12.130 |
| −10.807 | −10.790 | −10.777 | −10.762 | −10.751 | −10.738 | −10.730 | −10.719 | −10.713 | −10.704 | −10.699 | −10.692 | −10.687 | −10.682 | −10.677 | −10.672 | −10.668 |

| | | | | | | | | | | | | | | | | |
|---|---|---|---|---|---|---|---|---|---|---|---|---|---|---|---|---|
| 6.440 | 6.442 | 6.443 | 6.445 | 6.447 | 6.448 | 6.450 | 6.452 | 6.453 | 6.455 | 6.457 | 6.458 | 6.460 | 6.462 | 6.463 | 6.465 | 6.467 |
| −11.362 | −11.362 | −11.364 | −11.364 | −11.366 | −11.367 | −11.370 | −11.371 | −11.376 | −11.377 | −11.383 | −11.386 | −11.393 | −11.396 | −11.404 | −11.409 | −11.418 |
| −12.043 | −12.010 | −11.977 | −11.944 | −11.911 | −11.877 | −11.845 | −11.810 | −11.779 | −11.744 | −11.712 | −11.678 | −11.647 | −11.613 | −11.582 | −11.547 | −11.516 |
| −12.138 | −12.145 | −12.153 | −12.160 | −12.169 | −12.177 | −12.187 | −12.195 | −12.206 | −12.214 | −12.225 | −12.234 | −12.245 | −12.255 | −12.266 | −12.275 | −12.285 |
| −10.663 | −10.658 | −10.653 | −10.647 | −10.643 | −10.636 | −10.631 | −10.622 | −10.616 | −10.607 | −10.600 | −10.589 | −10.581 | −10.568 | −10.558 | −10.544 | −10.533 |

| | | | | | | | | | | | | | | | | |
|---|---|---|---|---|---|---|---|---|---|---|---|---|---|---|---|---|
| 6.468 | 6.470 | 6.472 | 6.473 | 6.475 | 6.477 | 6.478 | 6.480 | 6.482 | 6.483 | 6.485 | 6.487 | 6.488 | 6.490 | 6.492 | 6.493 | 6.495 |
| | | | | | | | | | | | | | | | | |
| −11.424 | −11.433 | −11.440 | −11.451 | −11.459 | −11.470 | −11.479 | −11.490 | −11.499 | −11.510 | −11.519 | −11.530 | −11.539 | −11.550 | −11.559 | −11.569 | −11.577 |
| −11.483 | −11.452 | −11.418 | −11.387 | −11.354 | −11.323 | −11.291 | −11.260 | −11.227 | −11.196 | −11.164 | −11.133 | −11.100 | −11.068 | −11.035 | −11.003 | −10.969 |
| | | | | | | | | | | | | | | | | |
| −12.294 | −12.304 | −12.313 | −12.322 | −12.332 | −12.341 | −12.350 | −12.359 | −12.368 | −12.377 | −12.386 | −12.394 | −12.403 | −12.412 | −12.419 | −12.428 | −12.434 |
| −10.517 | −10.504 | −10.486 | −10.471 | −10.451 | −10.433 | −10.412 | −10.391 | −10.368 | −10.344 | −10.318 | −10.292 | −10.264 | −10.235 | −10.205 | −10.175 | −10.144 |

| | | |
|---|---|---|
| 6.497 | 6.498 | 6.500 |
| | | |
| −11.587 | −11.595 | −11.604 |
| −10.936 | −10.902 | −10.870 |
| | | |
| −12.442 | −12.447 | −12.454 |
| −10.113 | −10.081 | −10.049 |
