# Supplementary material for: Functional decline of the precuneus associated with mild cognitive impairment: Magnetoencephalographic observations
Source: PLoS One. 2020 Sep 28;15(9):e0239577. doi: 10.1371/journal.pone.0239577 (PMC7521706; doi:10.1371/journal.pone.0239577)
Supplement: S3 Data — (PDF) [file pone.0239577.s003.pdf]

Group	id	MoCA		Begin	Mid	End	Hold		Begin(L)	Begin(R)		Mid(L)	Mid(R)		End(L)	End(R)		Hold(L)	Hold(R)
High	E51	27		−5.238	−10.023	−9.081	−7.657		−4.450	−6.026		−8.935	−11.110		−7.777	−10.385		−6.946	−8.369
High	E52	27		−0.820	−3.104	−2.902	−3.392		−0.557	−1.084		−3.269	−2.940		−2.772	−3.031		−3.402	−3.381
High	E53	26		−6.310	−7.189	−7.660	−10.200		−4.511	−8.110		−5.852	−8.526		−6.216	−9.105		−9.383	−11.018
High	E57	26		−9.483	−13.497	−14.341	−13.545		−8.626	−10.340		−12.605	−14.388		−13.857	−14.826		−12.803	−14.286
High	E63	27		−3.961	−6.559	−4.234	−5.340		−3.410	−4.513		−5.993	−7.125		−3.931	−4.537		−4.708	−5.972
High	E67	30		−11.232	−18.235	−16.827	−16.063		−11.188	−11.275		−18.031	−18.440		−16.675	−16.978		−15.764	−16.362
High	E70	26		−2.482	−2.192	−1.884	−7.038		−2.606	−2.358		−1.871	−2.512		−1.037	−2.732		−6.189	−7.886
Low	E8	25		−2.571	−3.120	−4.511	−7.210		−3.053	−2.088		−3.530	−2.710		−5.215	−3.807		−7.755	−6.664
Low	E11	19		−0.570	−2.446	−3.060	−4.685		−1.222	0.083		−3.140	−1.752		−3.467	−2.653		−5.100	−4.269
Low	E55	23		−0.775	−1.853	−2.269	−1.417		−0.972	−0.578		−1.824	−1.882		−2.295	−2.243		−1.194	−1.641
Low	E56	24		−7.462	−13.538	−16.695	−17.877		−8.280	−6.644		−13.906	−13.171		−17.444	−15.947		−19.501	−16.253
Low	E58	23		−2.019	−1.617	−0.472	−0.217		−2.395	−1.642		−1.826	−1.407		−0.329	−0.616		0.152	−0.586
Low	E59	25		10.998	34.822	41.761	30.418		10.972	11.025		36.113	33.532		44.299	39.222		33.313	27.524
Low	E60	22		2.836	−1.537	−1.429	−5.603		2.629	3.043		−1.283	−1.791		−1.455	−1.404		−4.832	−6.374
Low	E61	23		−3.717	3.701	0.146	0.345		−2.601	−4.833		8.344	−0.943		3.621	−3.328		2.423	−1.734
Low	E62	20		1.778	10.526	8.464	−1.862		0.720	2.836		9.902	11.149		7.665	9.263		−1.924	−1.800
Low	E64	24		−3.334	−4.325	−3.692	−1.842		−3.556	−3.111		−4.333	−4.316		−3.467	−3.917		−1.665	−2.019
Low	E66	25		−12.741	−13.196	−14.095	−16.149		−13.671	−11.811		−12.906	−13.486		−14.400	−13.790		−17.130	−15.168
Low	E68	20		18.819	38.259	18.455	0.607		16.931	20.708		36.209	40.309		17.215	19.696		0.127	1.087
Low	E69	24		−9.638	−10.760	−12.763	−10.761		−9.990	−9.285		−10.571	−10.949		−11.998	−13.529		−10.214	−11.308
